# Developing targeted therapies for neuroblastoma by dissecting the effects of metabolic reprogramming on tumor microenvironments and progression

**DOI:** 10.7150/thno.93962

**Published:** 2024-05-27

**Authors:** Wenyi Jin, Yubiao Zhang, Zhijie Zhao, Mingyong Gao

**Affiliations:** 1 Department of Orthopedics, Wenzhou Third Clinical Institute Affiliated to Wenzhou Medical University, The Third Affiliated Hospital of Shanghai University, Wenzhou People's Hospital, Wenzhou, China, 325041.; 2 Department of Orthopedics, Renmin Hospital of Wuhan University, No. 99 Zhangzhidong Road, Wuchang District, Wuhan, China, 430060.; 3 Department of Biomedical Sciences, City University of Hong Kong, Kowloon Tong, Hong Kong SAR, China, 999077.; 4 Department of Plastic and Reconstructive Surgery, Shanghai 9th People's Hospital, School of Medicine, Shanghai Jiao Tong University, 639 Zhi Zao Ju Road, Shanghai, China, 200011.

**Keywords:** metabolic reprogramming, etoposide, AZD7762, neuroblastoma, immune microenvironment

## Abstract

**Rationale:** Synergic reprogramming of metabolic dominates neuroblastoma (NB) progression. It is of great clinical implications to develop an individualized risk prognostication approach with stratification-guided therapeutic options for NB based on elucidating molecular mechanisms of metabolic reprogramming.

**Methods:** With a machine learning-based multi-step program, the synergic mechanisms of metabolic reprogramming-driven malignant progression of NB were elucidated at single-cell and metabolite flux dimensions. Subsequently, a promising metabolic reprogramming-associated prognostic signature (MPS) and individualized therapeutic approaches based on MPS-stratification were developed and further validated independently using pre-clinical models.

**Results:** MPS-identified MPS-I NB showed significantly higher activity of metabolic reprogramming than MPS-II counterparts. MPS demonstrated improved accuracy compared to current clinical characteristics [AUC: 0.915 vs. 0.657 (*MYCN*), 0.713 (INSS-stage), and 0.808 (INRG-stratification)] in predicting prognosis. AZD7762 and etoposide were identified as potent therapeutics against MPS-I and II NB, respectively. Subsequent biological tests revealed AZD7762 substantially inhibited growth, migration, and invasion of MPS-I NB cells, more effectively than that of MPS-II cells. Conversely, etoposide had better therapeutic effects on MPS-II NB cells. More encouragingly, AZD7762 and etoposide significantly inhibited in-vivo subcutaneous tumorigenesis, proliferation, and pulmonary metastasis in MPS-I and MPS-II samples, respectively; thereby prolonging survival of tumor-bearing mice. Mechanistically, AZD7762 and etoposide-induced apoptosis of the MPS-I and MPS-II cells, respectively, through mitochondria-dependent pathways; and MPS-I NB resisted etoposide-induced apoptosis by addiction of glutamate metabolism and acetyl coenzyme A. MPS-I NB progression was fueled by multiple metabolic reprogramming-driven factors including multidrug resistance, immunosuppressive and tumor-promoting inflammatory microenvironments. Immunologically, MPS-I NB suppressed immune cells via *MIF* and *THBS* signaling pathways. Metabolically, the malignant proliferation of MPS-I NB cells was remarkably supported by reprogrammed glutamate metabolism, tricarboxylic acid cycle, urea cycle, etc. Furthermore, MPS-I NB cells manifested a distinct tumor-promoting developmental lineage and self-communication patterns, as evidenced by enhanced oncogenic signaling pathways activated with development and self-communications.

**Conclusions:** This study provides deep insights into the molecular mechanisms underlying metabolic reprogramming-mediated malignant progression of NB. It also sheds light on developing targeted medications guided by the novel precise risk prognostication approaches, which could contribute to a significantly improved therapeutic strategy for NB.

## Introduction

Neuroblastoma (NB), a malignant tumor that generally affects young children, originates from dysplasia of the sympathetic nervous system and has a dramatic clinical course that ranges from spontaneous regression to metastasis, infinite proliferation, and aggressive progression 1, 2. Despite intensified multimodal treatments currently used including chemotherapy, radiotherapy, surgery, stem cell transplantation, and retinoic acid, the 5-year survival rate in high-risk NB patients remains less than 40%, while the cure rate increases in patients with more benign NB subtypes 2, 3. These treatments currently used for NB are applied empirically, urgently calling for clear biological or molecular markers to guide therapy and achieve more effective outcomes 3. Additionally, side effects caused by the empirical therapies have greatly compromised treatment benefits. Resultantly, those high-risk patients with unfavorable prognoses need be identified in advance with accurate approaches to allow for tailored and precise medication.

The classification of *MYCN* status, either amplified or non-amplified, has been established as the principal diagnostic indicator for assessing relapse and mortality risk in NB patients 4. Despite its strong correlation to poor outcomes, *MYCN* amplification presents in only about 20% of all NB subjects and 45% of those classified as high-risk NB 5. So far, the most important clinical parameter in patient stratification has been the international neuroblastoma staging system (INSS), a postsurgical staging system that may not consistently predict NB behaviors 6, 7. While the international neuroblastoma risk group (INRG) staging system contributes substantially to preoperative risk stratification 8, the pathological and clinical parameters employed in INRG only reflect partially the underlying oncology. Consequently, new strategies for risk evaluation focusing on the biological attributes of malignant tumors hold great potential for enhancing prognostic accuracy and further enabling more targeted and individualized therapies in the clinical treatment of NB 2.

Metabolic reprogramming is fundamental to the onset and progression of various malignant tumors 9-11. Highly heterogeneous metabolic processes, including glucose utilization 12, mitochondrial and lipid metabolism 9, 13, and the pentose phosphate pathway 14, typically involve multiple malignant characterizations, including infinite proliferation potential 15, avoidance of immunosurveillance 16 and anti-cancer drug resistance 17, 18, in cancer cells. Furthermore, cell-autonomous genetic alterations are of great importance in metabolic reprogramming 19, and abnormal metabolism significantly affects the clinical progression of NB 20, 21. Unfortunately, how genetic heterogeneity affects metabolic reprogramming and the association mechanisms between clinical prognosis and drug sensitivity in NB still need to be elucidated.

**Figure 1:**
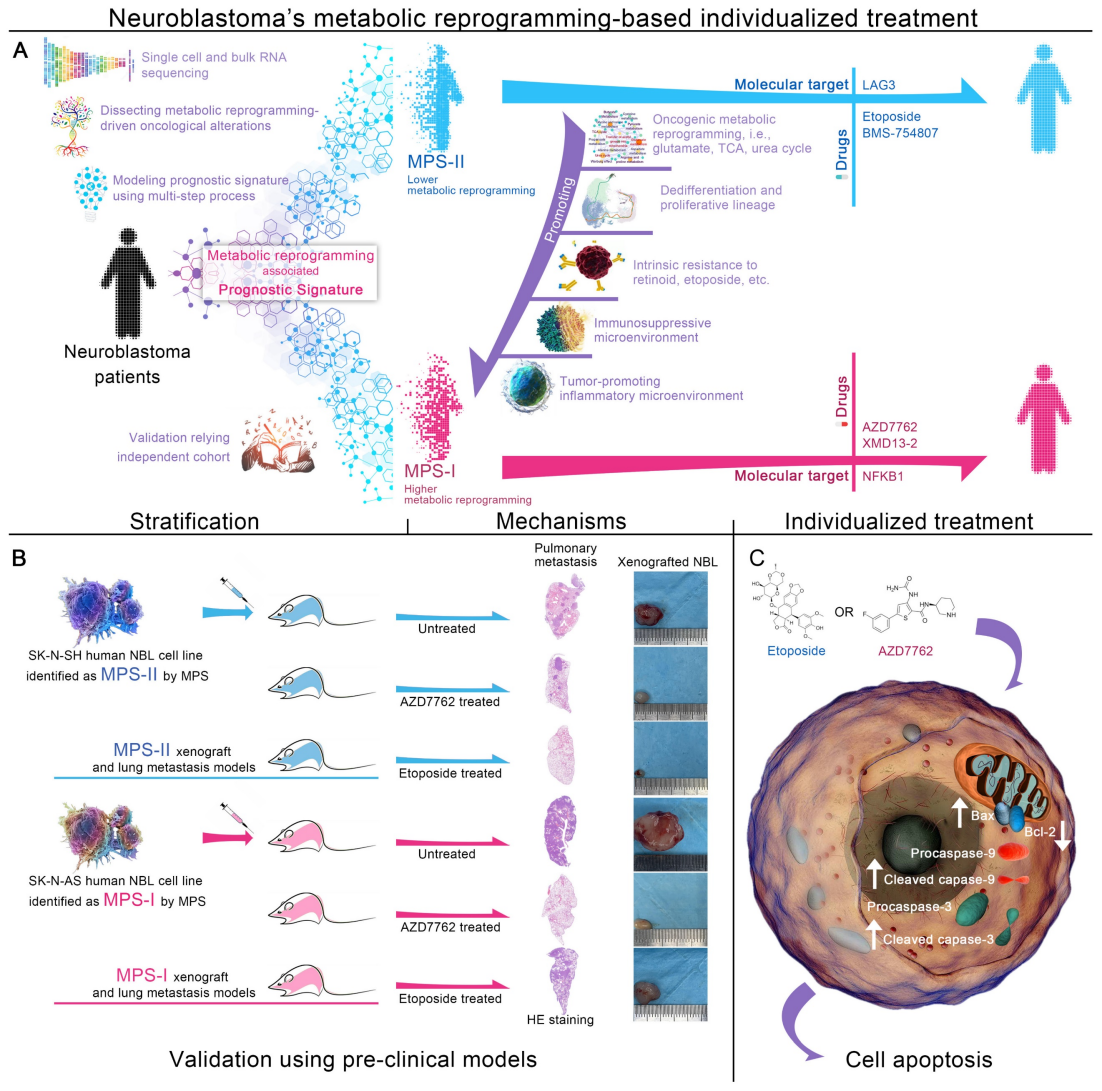
** Graphical abstract. (A)** Overall design of MPS-based individualized treatment. **(B)** Therapeutic effects of MPS-based individualized treatment in the pre-clinical models. **(C)** Molecular mechanism of pro-apoptotic effects of MPS-based individualized treatment.

The present study, adopting a multi-step process including machine learning algorithms for the first time, organized and retrospectively analyzed 946 clinical samples to address the aforementioned issues. Based on the exploration of the molecular mechanism related to metabolic reprogramming and the specific NB microenvironments, a novel metabolic reprogramming-based clinical stratification strategy has been developed and cross-validated to perform more accurate risk stratification and further guide more effective and targeted treatments of NB. The main findings of the present study were shown in Figure 1.

## Methods

### Study design and patients

We retrospectively analyzed RNA-sequencing data from 946 samples acquired from Genotype Tissue Expression (GTEx), Therapeutically Applicable Research To Generate Effective Treatments (TARGET), and Gene Expression Omnibus (GEO) databases 22. The control cohort (n=298) was constructed using normal adrenal gland samples, which were screened from the RNA-sequencing data of 7862 normal frozen samples obtained from the GTEx database; 150 NB samples acquired from the TARGET database and 498 obtained from the GEO database were collected to construct the NB cohort. Additionally, a GEO dataset including 283 patients was included; after the exclusion of patients lacking clinical parameters, a total of 276 patients was employed as an external test cohort. Sources and clinical parameters of all samples included in this study are summarized in Table S1. The gene expression profiles of all samples were standardized to Fragments Per Kilobase per Million (FPKM), normalized to eliminate the batch error, and then log transformed to facilitate subsequent analyses.

Data were collected from October 8 to December 10, 2019. As shown in the previously reported method 23, 24, the NB cohort was randomly split approximately in half into a training cohort and test cohort using the caret package in R; normalization and log transformation were performed using the limma package in R 25. The following formula was used for log transformation:

log_2_ converted expression = log_2_ (FPKM+1)

### Construction and validation of MPS on its prognostic potential compared to the current clinical prognostic approaches

For detailed MPS construction methods, please see Methods S1 to S2.

The prognostic value of the MPS was cross-evaluated in patients with NB in the training and test cohorts. The prognostic prediction was determined by calculating the area under the time-dependent receiver operating characteristic (TROC) curve (AUC). Patients in each cohort were grouped into MPS-I and MPS-II groups according to an optimal cutoff calculated from the TROC curve. The product-limit method (Kaplan-Meier survival curve) was employed to conduct the survival analysis. The concordance of the prognosis predicted using the MPS was measured using the index of concordance (C-index), and calibration was investigated by constructing a calibration plot. Because the MPS and overall survival time are continuous variables, Spearman's correlation coefficient was calculated to assess the correlation between the two parameters. Univariate and multivariate Cox analyses were conducted to determine whether the MPS represented an independent prognostic factor (p-value < 0.05 in both analyses) 26.

The prognostic prediction accuracy and goodness of fit between clinical characteristics in patients with NB and the MPS were also compared using the TROC curve, net reclassification improvement (NRI), and decision curve analysis (DCA) in the training cohort and test cohort. NRI is a method that measures the changes in the number of correct classifications of the research objects of the two models to compare the accuracy of the prediction capabilities of the two models 27. DCA is used to comprehensively evaluate the goodness of fit of the prediction models 28, 29.

The optimal cutoff was determined using a method reported in a previous study 24. The C-index and calibration plot was assessed with cross-validation in 1000 steps of randomization using the bootstrap method. The Kaplan-Meier survival curve, TROC curve, C-index, calibration plot, NRI, and DCA curve were constructed using the rms, survival, survivalROC, survminer, pec, survIDINRI and rma packages in R.

### Exploration of underlying mechanisms in the development of MPS-I NB

Metabolic reprogramming-associated genes (MG) and prognostic MGs (PMG) regulated signaling pathway pathways, biological processes (BP), molecular functions (MF), and cellular components (CC) were investigated using enrichment analyses. Moreover, the specified overall tumor-microenvironment, immune and inflammatory environment of MPS-I NB were elucidated by ESTIMATE algorithm and single-sample Gene Sets Enrichment Analysis (ssGSEA). Molecular docking was conducted using Mcule platform (mucle.com). For detailed methods, please refer the Methods S3 to S5.

### Nomogram and integrated prognostic index by combining the MPS with clinical factors (IPI) generation

The MPS was visualized as a nomogram using the rms package in R. The IPI was modeled with survival tree, an interpretable machine learning model, according to the previous study.

### Potential therapeutic molecular targets and drugs sensitivity

For detailed information on the investigation of potential therapeutic molecular targets, please see Methods S6.

Data of Gene expression profiles of human cancer cell lines (CCLs) and GDSC-archived drug sensitivity data of CCLs were achieved from the Depmap database (depmap.org). The batch effect was removed using limma package. K-nearest neighbor (k-NN) imputation was applied to impute the missing AUC values. Before imputation, compounds with more than 20% of missing data were excluded 30. The antitumor effectiveness of 132 candidate compounds over 17 NB CCLs were analyzed for drug screening (p-value < 0.05 was considered statistically significant).

### Biological validation of MPS-based individualized treatment in the pre-clinical models

MPS-based individualized treatment was validated with *in vitro* and *in vivo* models.

For detailed methods of *in vitro* validation, please see Methods S7 to S11. The pro-apoptotic effects and molecular mechanisms of MPS-based individualized treatment on their respective sensitive NB subtype had been further elucidated, details please see Methods S12 to S15.

For detailed methods of *in vivo* validation please see Methods S16 and S17. The anti-metastatic effects of MPS-based individualized treatment were validated in the established pulmonary metastasis mouse models (Methods S18).

### Single cell RNA sequencing (scRNA) analyses and metabolite flux balance analyses

The scRNA data of 16 NB patients were included and analyzed in the present study. Raw data was pre-processed, clustered, annotated (Methods S19), and the DEGs were identified (Methods S19). The cell cycle, copy number variations, stemness of each cell type were measured (Methods S20). The biological function of each cell type, and the dysregulated signaling pathway within different types were quantified using enrichment analyses, GSEA, and AUCell (Methods S21). Afterward, the differentiation potential and developmental lineages were identified (Methods S22). CellChat were employed to elucidate the cell-cell communication patterns (Methods S23). The metabolite flux balance analyses were employed to unveil the metabolic signaling pathway alterations of NB cells (Methods S24).

### Energy metabolism analyses

The MPS-I NB cell line and the MPS-II NB cell line were respectively prepared as six biological replicates for liquid chromatography tandem mass spectrometry (LC-MS/MS) analysis (Methods S25). Briefly, HPLC-grade acetonitrile and methanol, along with MilliQ water, were utilized as solvents, sourced from Merck and Millipore respectively, while all standards and formic acid were procured from Sigma-Aldrich. Stock solutions of these standards were prepared at 1 mg/mL in methanol, stored at -20°C, and later diluted for analytical use. Sample processing involved thawing on ice, resuspension in ultrapure water, followed by repeated freezing, vortexing, and centrifugation steps to prepare the supernatant for LC-MS analysis. This analysis was conducted using a Waters ACQUITY H-Class system and a QTRAP® 6500+ LC-MS/MS System, employing an amide-based method with a specific solvent gradient and temperature conditions. The mass spectrometer operated in both positive and negative ion modes, using various settings to optimize detection and quantification of metabolites through multiple reaction monitoring, managed by Analyst and Multiquant software.

### Statistical analyses and visualization

R software (version 3.6.1) was employed for statistical analyses (Methods S26). For statistical significances judgment, “*” represented “*p*<0.05”, “**” represented “*p*<0.01”, and “***” represented “*p*<0.001”.

## Results

### Development of metabolic reprogramming-associated prognostic signature (MPS)

**Figure 2:**
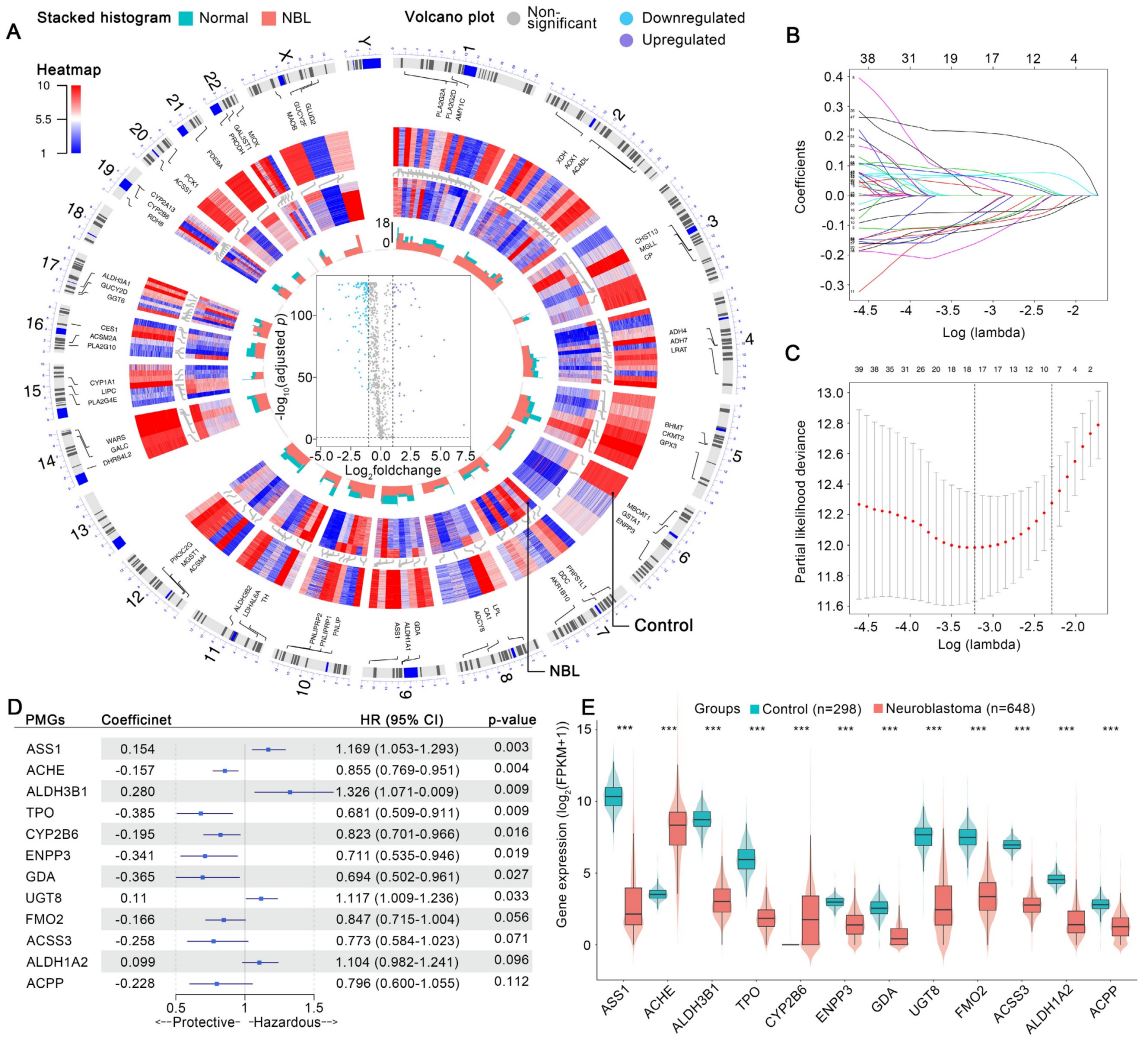
** Features selection and MPS modeling. (A)** Visualized variations in omics data and machine-learning analyzed results by generated circos plot, which showed the illuminating process of the aberrant expression profiles of metabolic genes in NB. From outermost track to innermost track, the following were exhibited in order: Track 1, homo sapiens chromosome coordinates and scales, and the result analyzed using the corresponding method in each gene were plotted, on all subsequent tracks, according to its chromosomal location. Track 2, the top 3 MGs ordered by log_2_foldchange in each chromosome were shown. Track 3, expression profiles of MGs in all normal samples of the control cohort. Track 4, expression profiles of MGs in all samples of the NB cohort. Track 5, stacked histogram exhibited the MGs mean expression in control or NB cohort; green represents the control cohort and red represents the NB cohort. Track 6, volcano plot of MGs expression changes in the NB cohort. **(B)** Expression of 12 selected MGs in neuroblastoma and normal tissue. **(C)** Each line represents the coefficient of a specific MG screened to have significant prognostic potential. **(D)** A plot of partial likelihood deviance. **(E)** A forest plot of MPS coefficients and its subcomponent MGs hazard ratios.

**Figure 3:**
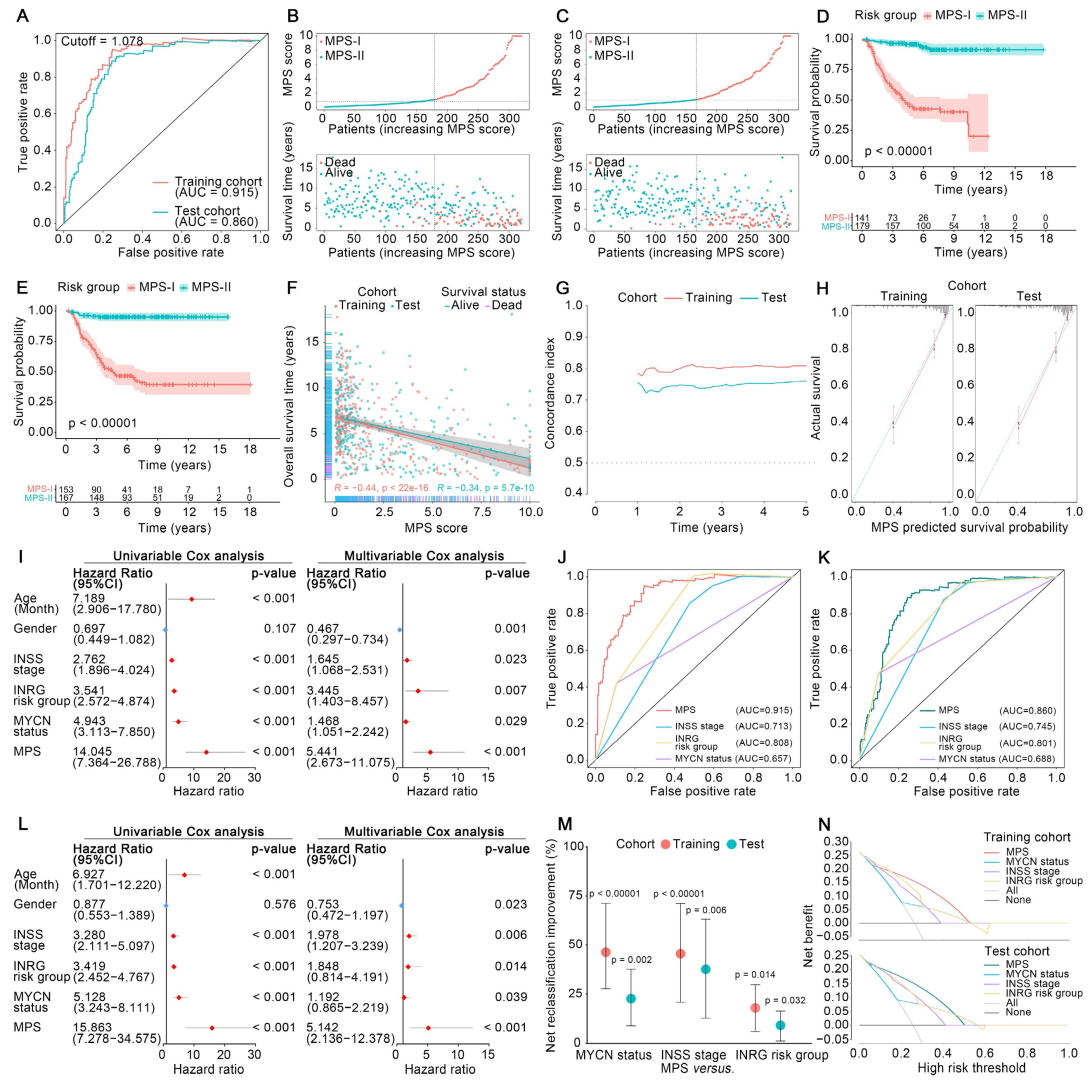
** Validation of MPS, and comparison between MPS and clinical stratification approaches. (A)** TROC curve of the MPS for the training and test cohort, an optimal cut-off value and AUC were marked in the plot. **(B)** Distribution of the MPS, survival time and survival status of NB patients in the training cohort. **(C)** Distribution of the MPS, survival time and survival status of NB patients in the test cohort. **(D)** Kaplan-Meier survival analysis for patients at risk-stratified by MPS in the training cohort. The chart below the Kaplan-Meier survival curve shows the number of NB patients at different time points in the MPS-I group and the MPS-II group. **(E)** Kaplan-Meier survival analysis for patients at risk-stratified by MPS in the test cohort. The chart below the Kaplan-Meier survival curve shows the number of NB patients at different time points in the MPS-I group and the MPS-II group. **(F)** Correlation analysis for MPS and overall survival time in training and test cohort. The survival status of each patient was marked in the plot. **(G)** Distribution of corrected C-index of MPS in training and test cohort. **(H)** Calibration plot of MPS reflects the consistency between the prediction of MPS and the actuality in the training and test cohort, a 45-degree diagonal dotted line represents the 100% ideal fit. **(I)** Independence analysis based on uni/multi-variable cox analysis in the training cohort. **(J)** TROC curve and the corresponding AUC of the MPS and clinical characteristics in the training cohort. **(K)** TROC curve and the corresponding AUC of the MPS and clinical characteristics in the test cohort. **(L)** Independence analysis based on uni/multi-variable cox analysis in the test cohort. **(M)** NRI of MPS compared to other clinical features in the training / test cohort. **(N)** DCA curve of MPS and other clinical characteristics in the training and test cohort.

The aberrant expression profiles of MGs were first investigated. As shown in Figure 2 A, 42 MGs were found to be upregulated in the NB cohort, while 110 MGs were downregulated. In the training cohort, univariate Cox analyses revealed that 12 upregulated and 79 downregulated MGs contributed substantially to the unfavorable prognosis of patients with NB, while 30 upregulated and 31 downregulated MGs were protective factors (Table S2). The MGs affecting the prognosis of patients with NB were defined as prognostic MGs (PMGs). Then, a LASSO regression selected 4 upregulated and 14 downregulated key PMGs for MPS modeling (Figure 2 B and C). The MPS was initially modeled using multivariate Cox regression with a stepwise method, and a final MPS was constructed with 12 key PMGs (Figure 2 D), which also presented differential expressions between the NB and normal tissues (Figure 2 E). Finally, all subjects were identified and assigned the corresponding MPS score, then stratified into MPS-I and MPS-II groups as per the optimal cutoff value of 0.493 (Figure 3 B and C), which was obtained by using the TROC curve in the training cohort (Figure 3 A).

### MPS outperforms current clinical prognostication approaches in predicting prognosis

The MPS was demonstrated to have outstanding prognostic value, which was cross-validated in the training and test cohorts. The AUC for the TROC curve of the MPS of the training cohort was calculated as 0.915, whereas the AUC of the test cohort was 0.860 (Figure 3 A). The MPS was further validated using an external test cohort. Unfortunately, the Gene Expression Omnibus (GEO) database lacked datasets that contained both clinical data and all key PMGs. After considering the sample size and the completeness of the clinical data, GSE85047 was included as an external test cohort. The survival analysis of the key PMGs in the GSE85047 cohort was performed using the Kaplan-Meier method 31, 32. As indicated in Figure S1, significant survival differences were found between high- and low-expression groups of* ACHE*, *ACSS3*, *ALDH1A2*, *ALDH3B1*, and *ENPP3*, which were highly consistent with the aforementioned findings (Table S2). These findings corroborated that the MPS has outstanding discrimination ability (the AUC was much higher than 0.75) 33. Patients in the MPS-I group had significantly lower survival rates in both the training and test cohorts than those in the MPS-II group (Figure 3 D and E), and there was a significant negative correlation between the MPS score and the overall survival time (Figure 3 F). Moreover, the C-index measured in both the training cohort (0.807 at 5 years) and the test cohort (0.759) confirmed the favorable concordance of the MPS (Figure 3 G), and the calibration plots also verified that the MPS presented good calibration (Figure 3 H), indicating that the MPS could accurately estimate absolute risk 33.

Furthermore, MPS was an independent prognostic factor that could further stratify clinical NB subtypes and was shown to be superior to the clinical prognostication approaches currently used for prognostic risk stratification. As indicated in Figure 3 I and L, the MPS demonstrated statistical significance in both multivariate and univariate Cox analyses (all *p* < 0.001) and was further identified as a hazardous factor with higher hazard ratios (up to 15.86) than *MYCN* status, INSS stage, and INRG stratification. The MPS could stratify patients into subgroups with significantly different prognoses when further limited to those with *MYCN* not amplified, INSS stages 1/2/3/4, INRG L1/L2/M, and age > 18-month diseases (*p* < 0.05, Figure S2 A and B). However, the MPS failed to stratify patients in subgroups of *MYCN* amplified, INSS 4S, and INRG MS. A rational explanation might be that patients of INSS 4S and INRG MS are indeed special subgroups with favorable clinical prognosis, and the *MYCN* amplification was corroborated as a direct factor initiating high-risk NB and represented a subgroup with poor prognosis. Only quite a few patients were identified as MPS-II in the *MYCN*-amplified NB of training set due to high-risk nature of *MYCN* amplification, and the limited sample size could also obscure the genuine difference between groups. Nevertheless, direct evidence from the test set indicated that MPS-II patients in the MYCN-amplified group had a significantly higher survival rate than those in MPS-I (p < 0.05), suggesting that MPS stratification may have clinical potential to further differentiate patients from the MYCN-amplified group. Furthermore, the MPS showed superior ability of discrimination (Figure 3 J and K) and presented net reclassification improvement (NRI) of 39.8%, 44.4%, and 18.2% compared with the *MYCN* status, INSS stage, and INRG stratification, respectively (Figure 3 M). Decision curves also demonstrated that the net benefit of the MPS stratification was significantly greater than current clinical prognostication approaches (Figure 3 N). The proportions of each INSS stage and INRG stratification within the MPS subgroups were also investigated, and it was demonstrated that MPS outperformed a genetic prognostic signature based on all genes. The resultant data was presented in Result S1 and S2, Figure S3 and S4, and Table S3.

### MPS-I NB group showed a high degree of metabolic reprogramming

**Figure 4:**
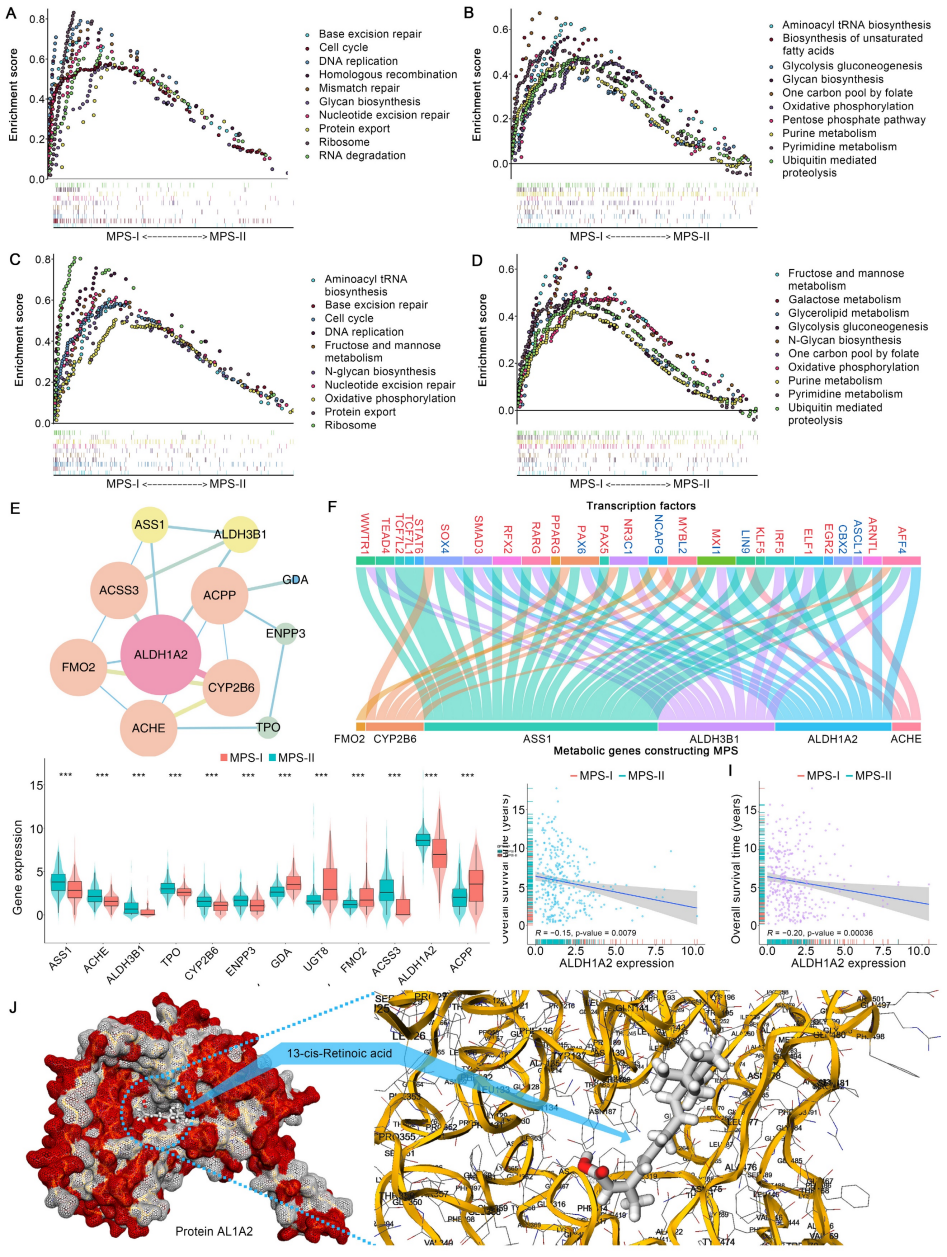
** Metabolic reprogramming characteristics exploration. (A)** GSEA analysis between MPS-I and MPS-II NB patients in the training cohort, top 10 reprogrammed pathways were shown. **(B)** GSEA analysis between MPS-I and MPS-II NB patients in the training cohort, top 10 reprogrammed metabolic pathways were shown. **(C)** GSEA analysis between MPS-I and MPS-II NB patients in the test cohort, top 10 reprogrammed pathways were shown. **(D)** GSEA analysis between MPS-I and MPS-II NB patients in the test cohort, top 10 reprogrammed metabolic pathways were shown. **(E)** PPI network analysis for kPMGs. **(F)** TF-kPMG regulation network. In the legend of transcription factors, red represents the positive regulation and blue indicates the negative regulation. **(G)** In the training cohort, different expressions of kPMGs in MPS-I and MPS-II NB patients.** (H)** Correlation analysis of *ALDH1A2* expression and overall survival time, in the training cohort. **(I)** Correlation analysis of *ALDH1A2* expression and overall survival time, in the test cohort. **(J)** Results of molecular docking.

The essential signaling pathways involved in metabolic reprogramming were significantly upregulated and had a synergistic effect on the malignant progression of MPS-I NB. The Gene Set Enrichment Analysis (GSEA) results revealed that most of the pivotal biological processes (BPs), molecular functions (MFs), and cellular components (CCs) in cancer progression 34, 35, such as cell cycle, DNA replication, glycolysis, and protein synthesis, were significantly upregulated in the MPS-I NB group of the training cohort (Figure 4 A). The test cohort had similar outcomes (Figure 4 C). Notably, it was revealed that the majority of typical metabolic signaling pathways including carbon and glycine metabolism, glycolysis, base and protein metabolism, pentose phosphate pathway, and fatty acid biosynthesis, were upregulated in the MPS-I NB group (Figure 4 B and D). These pathways played critical roles in activating pivotal processes during NB progression including drug resistance 36, *MYCN* transcription 37, cell proliferation 38, etc.

Furthermore, the ssGSEA algorithm was employed to evaluate individualized differences in metabolic reprogramming pathways between the MPS-I and MPS-II NB groups (Figure S5 and Table S4). In the training cohort, the MPS-I NB group had 25 pathways, including the metabolism of carbohydrates (galactose [log_2_Fold-Change: 0.25], fructose and mannose [0.21], glycolysis gluconeogenesis [0.16], etc.), genetic materials (pyrimidine [0.14], purine [0.13], nucleotide sugar [0.17], etc.), lipid (glycerolipid [0.11], fatty acid [0.07], etc.), amino acid (cysteine and methionine [0.16], .etc.), and others (drugs [0.06], ascorbate [0.14], glutathione [0.12], etc.), significantly upregulated and only sulfur metabolism [-0.08] was downregulated. The results in the test cohort were similar to those in the training cohort (Table S4), and they strongly agreed with the aforementioned results of the GSEA. These findings indicated that metabolic reprogramming-associated dysregulations of BPs, CCs, MFs, and pathways were significantly associated with unfavorable prognoses of MPS-I NB.

### Metabolic reprogramming-mediated retinoid resistance promoted MPS-I NB malignant progression

Following MPS validation, there was a focus on elucidating the molecular mechanisms involved in the progression of MPS-I NB to facilitate the design of personalized treatments. Enrichment analyses found that the abnormally expressed MGs were mainly involved in intracellular energy support, membrane integrity, and metabolism of antitumor drugs (Table S5 and S6), and these findings were re-confirmed in the test cohort. These findings suggest that metabolic abnormalities, including energy support, may promote NB development 39, 40. Remarkably, certain functions or pathways associated with drug resistance, including retinoid resistance (cytochrome P450-associated pathway 41, 42, retinoid and retinol metabolism 43), and terpenoid metabolic process 43-45, were overrepresented. Subsequently, these encouraging findings in the key PMGs constructing MPS (kPMGs) were thoroughly validated. A Gene Ontology (GO) enrichment analysis demonstrated that most processes were related to metabolisms, such as drug metabolism and monooxygenase activity, cellular metabolism, purine nucleobase and nucleotide metabolism, and lipid and protein biosynthesis (Table S7). The Kyoto Encyclopedia of Genes and Genomes (KEGG) analysis confirmed that the kPMGs were mainly involved in pathways associated with retinoid resistance, including cytochrome P450-related metabolism and retinol metabolism (Table S8) 43, 45. Further protein-protein interaction (PPI) network analysis identified *ALDH1A2*, a risk factor in the MPS (Figure 2 E), as a hub gene of kPMGs (Figure 4 E). Previous studies showed that *ALDH1A2* upregulates resistance to retinoic acid derivatives 46. Based on these findings, the expression of kPMGs (particularly *ALDH1A2*) in the MPS-I and MPS-II NB groups was further investigated. As expected, *ALDH1A2* was significantly overexpressed in the MPS-I NB group (Figure 4 G and Figure S6) and was not only identified as a hazardous factor by univariate Cox regression (HR: 1.26, 95%CI: 1.15‐1.38, *p* < 0.001; Table S9), but also negatively correlated with the overall survival time of patients with NB (Figure 4 H and I). Due to the widespread use of isotretinoin (13-cis-retinoic acid) for differentiation induction treatments following NB in clinical settings 47, the binding orientation and affinity between 13-cis-retinoic acid and *AL1A2* (a protein coded by *ALDH1A2*) were further investigated. Their docking poses confirmed that *AL1A2* had a high binding affinity for 13-cis-retinoic acid (docking score was -7.6, more negative values indicating higher binding affinity), and indicated that* AL1A2* may have a role in the metabolism of 13-cis-retinoic acid (Figure 4 J). These findings strongly suggested that MPS-I NB had intrinsic drug resistance (IDR) to retinoids and retinoic acid derivatives, which may cause a deterioration of NB and thereby result in unfavorable prognostic outcomes in patients with MPS-I NB.

Additionally, transcription factors (TFs) may significantly affect genetic reprogramming 48. Correlation analysis of differentially expressed TFs (DETFs) involved in tumor progression and kPMGs revealed the TF-kPMG regulatory network (Figure 4 F and Table S10). Forty-three pairs of positive regulatory relationships and 15 pairs of negative regulatory relationships were confirmed.

### The immunosuppressive microenvironment potentiated the poor prognosis of MPS-I NB

**Figure 5:**
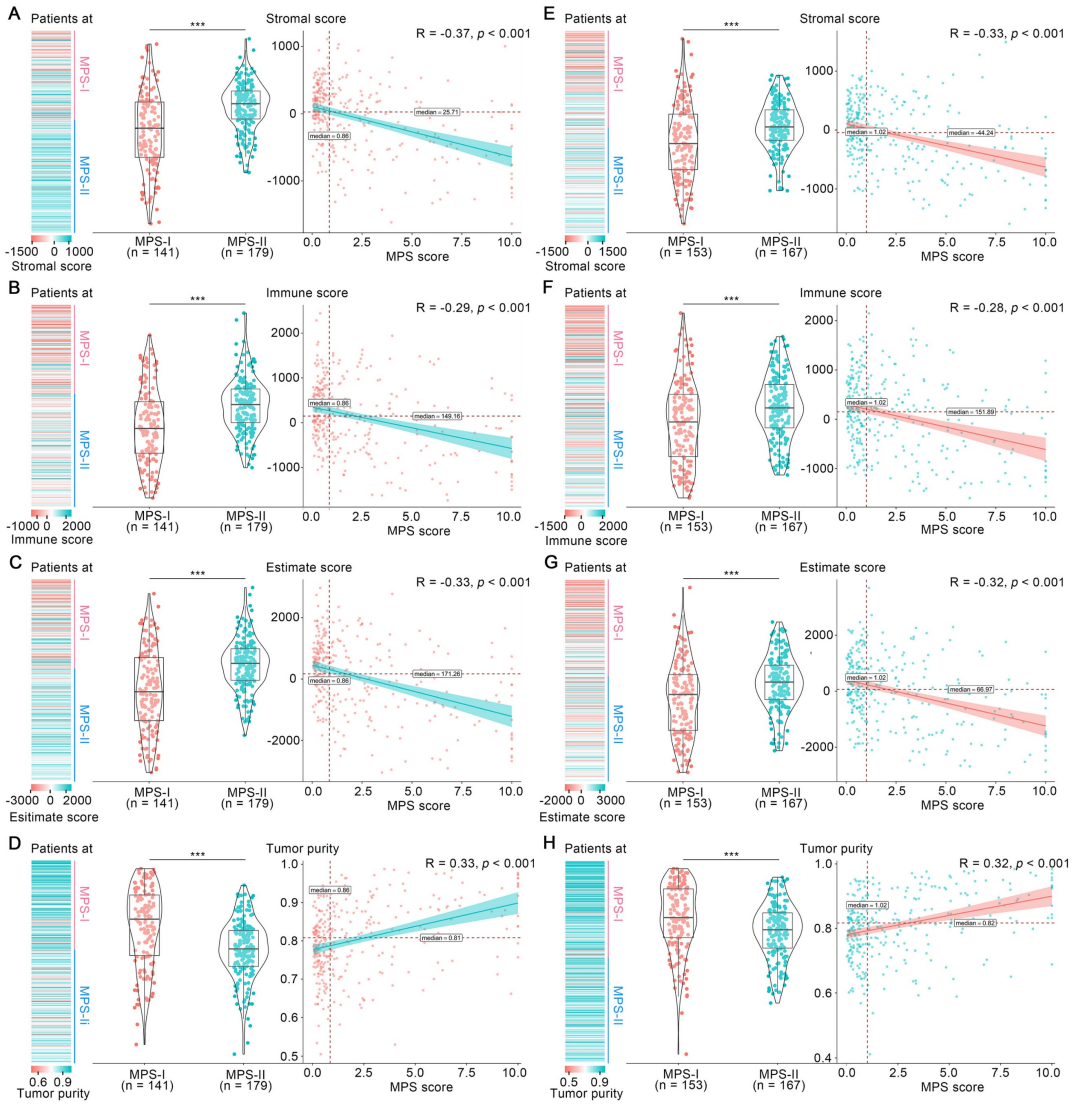
** Overall tumor-microenvironment of MPS-I and MPS-II NB.** In the training cohort, **(A), (B), (C)** and **(D)** represent the distribution, grouped comparison and correlation of Stromal score, Immune score, ESTIMATE score and Tumor purity, respectively. In the test cohort, **(E), (F), (G)** and **(H)** represents the distribution, grouped comparison and correlation of Stromal score, Immune score, ESTIMATE score and Tumor purity, respectively.

Tumor microenvironment has long been established as a significant determinant in NB progression 24, 34. Hence, the overall microenvironment of MPS-identified MPS-I and MPS-II NB was evaluated. As shown in Figure 5 A to C, the mean stromal score, immune score and ESTIMATE score in the MPS-I NB samples were -261.00 ± 581.19, -121.5 ± 861.87, and -382.5 ± 1332.61, respectively, which were significantly lower than those in the MPS-II NB samples with 94.62 ± 365.14, 347.25 ± 604.14, and 441.86 ± 846.45, respectively; and these scores were negatively correlated with the MPS score. In contrast, the tumor purity of MPS-I NB was significantly higher than that of MPS-II NB (0.84 ± 0.10 vs. 0.77 ± 0.08, *p* < 0.001) and was positively correlated with the MPS score (Figure 5 D). These findings were further confirmed in the test cohort (Figure 5 E to H) and the results indicated that MPS-I NB had a remarkable immunosuppressive microenvironment.

**Figure 6:**
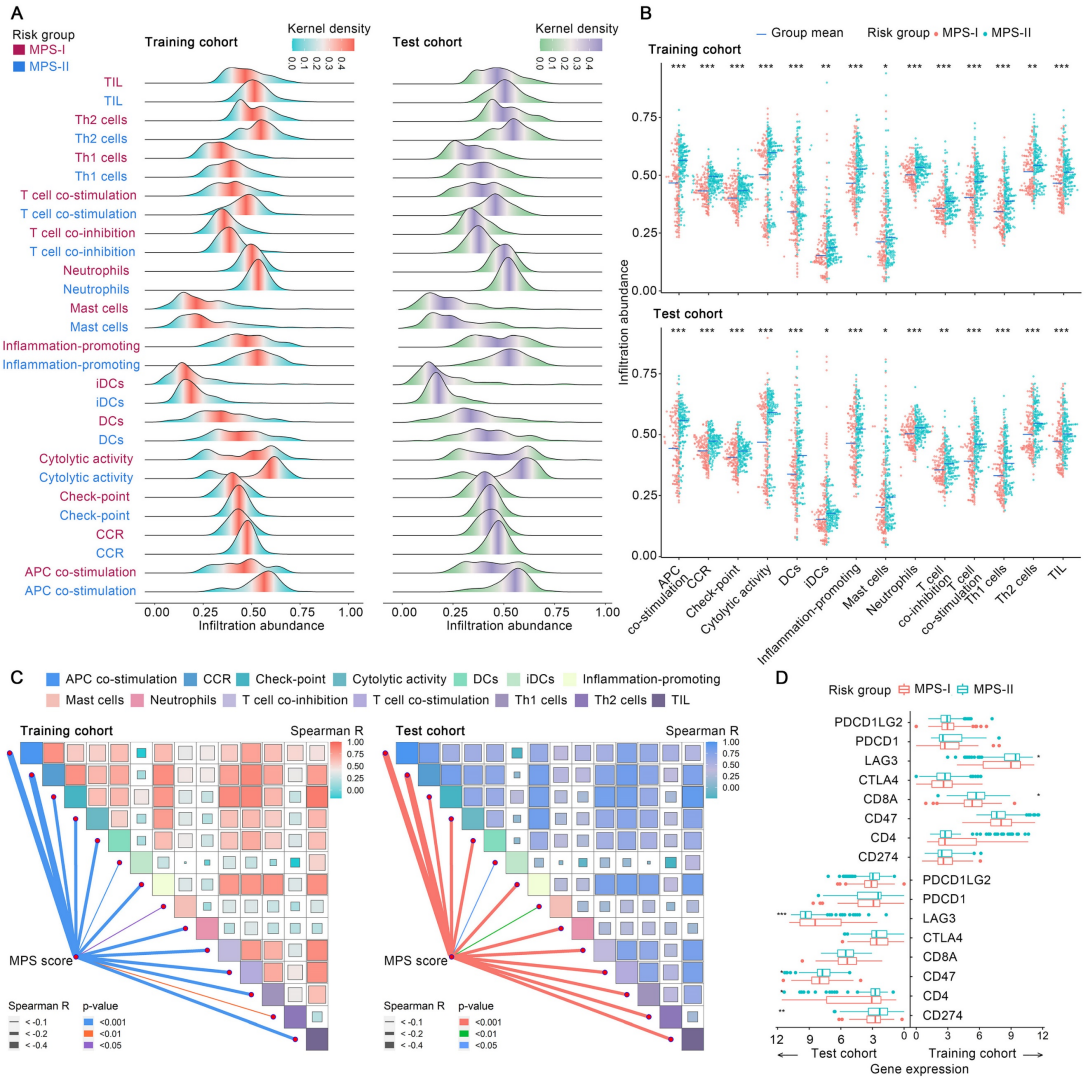
** The difference of immune microenvironment between MPS-I and MPS-II NB. (A)** Ridge plots exhibit the distribution and density of identified dysregulated immune cells' infiltration in the MPS-I and MPS-II NB. **(B)** Grouped comparison of these immune cells' infiltration between MPS-I and MPS-II NB. **(C)** Correlations among these immune cells and between these immune cells and MPS score. **(D)** Grouped comparison of the expression of critical immune molecules involving NB's development between MPS-I and MPS-II NB.

Subsequently, the immunosuppressive microenvironment in MPS-I NB (Figure S7) was further investigated. Among 28 immune cellular components, 14 presented significantly reduced infiltration abundance in the MPS-I NB group (Figure 6 A). These cell types included T helper 2 (Th2) cells (log_2_ Fold-change: -0.08), neutrophils (-0.09), T cell co-inhibition (-0.11), check-point (-0.11), interstitial dendritic cells(iDCs, -0.11), tumor infiltrating lymphocyte (TIL, -0.13), C-C chemokine receptor (CCR, -0.13), inflammation-promoting (-0.15), T helper 1 (Th1) cells (-0.15), cytolytic activity (-0.18), mast cells(-0.21), T cell co-stimulation (-0.21), antigen presenting cells (APC) co-stimulation (-0.27), and dendritic cells (DCs, -0.31), as shown in Figure 6 B and Table S11. Additionally, there was a positive correlation between the infiltration abundance of these 14 cellular components and all of these cellular components had a negative correlation with the MPS score, indicating that these immune cell components interact dynamically during NB progression (Figure 6 C). Collectively, such a significant differential profile in infiltration abundance and dynamic interaction network of key immune cells could indicate the cellular mechanisms underlying the malignant progression of MPS-I NB, eventually forming a specific immunosuppressive microenvironment that highly potentiates the poor prognosis of MPS-I NB.

Moreover, the profile differences of key immune checkpoint genes expressed in the MPS-I and MPS-II NB samples were examined and it was found that the high expression of lymphocyte-activating gene 3 (*LAG3*) in MPS-II NB indicated that immunotherapy targeting *LAG3* could be beneficial for the clinical treatment of patients with MPS-II (Figure 6 D and Result S3)*.*

### Specific inflammatory microenvironment also fueled the progression of MPS-I NB

**Figure 7:**
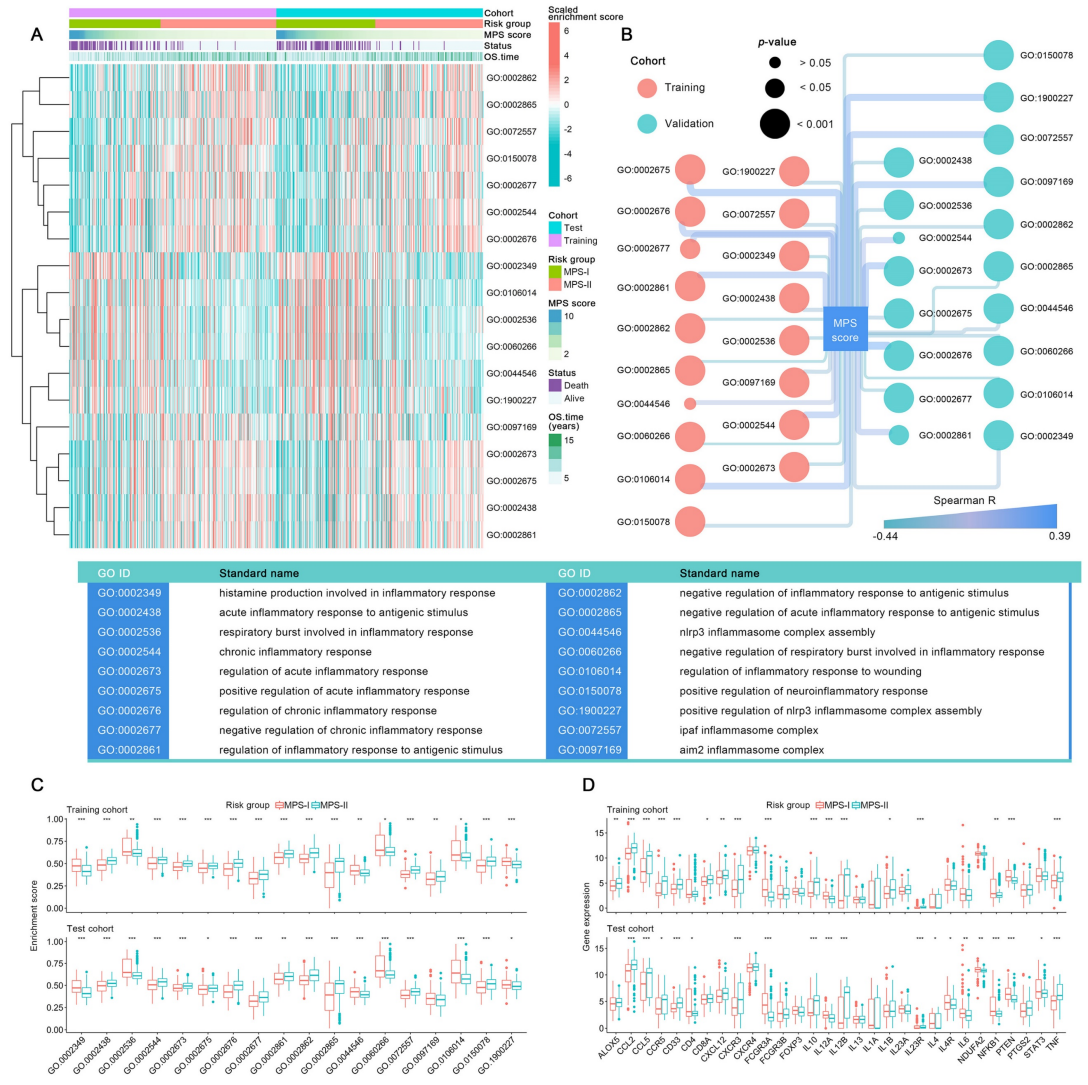
** Systematically analyses of the inflammatory microenvironment in the MPS-I and MPS-II NB. (A)** Comprehensive view of identified dysregulated BP, CC, and MF in the MPS-I and MPS-II NB. **(B)** Correlations between MPS score and these BP, CC, and MF. **(C)** Grouped comparison of these BP, CC, and MF enrichment scores between MPS-I and MPS-II NB. **(D)** Grouped comparison of critical inflammatory molecules' expression involving NB's development between MPS-I and MPS-II NB.

Since the inflammatory responses and molecules symphonize the vital pathophysiologic development of NB 49, the differential profile of the inflammatory microenvironment in MPS-I and MPS-II NB was examined. As shown in Figure 7 A, among 45 sorts of inflammation-associated BPs, CCs, and MFs, 6 were upregulated, and 12 were downregulated in the MPS-I NB group. The upregulated inflammation-associated BPs, CCs, and MFs included histamine production involved in the inflammatory response, regulation of the inflammatory response to wounding, negative regulation of respiratory burst involved in the inflammatory response, respiratory burst involved in the inflammatory response, nod-like receptor protein 3 (NLRP3) inflammasome complex assembly, and positive regulation of NLRP3 inflammasome complex assembly with log_2_Fold-Change of 0.15, 0.1, 0.09, 0.09, 0.08, 0.07, respectively (Figure 7 C and Table S12). The downregulated types included positive regulation of acute inflammatory response, chronic inflammatory response, absent in melanoma 2 (AIM2) inflammasome complex, and regulation of acute inflammatory response (Figure 7 C and Table S12). Further correlation analysis confirmed that the enrichment abundances of these inflammation-associated BPs, CCs, and MFs were significantly correlated with the MPS scores. The changing trend of the correlation coefficient was consistent with their differential trend of enrichment abundances (Figure 7 B). These findings indicated a strong correlation between malignant progression and poor prognosis of patients with MPS-I NB and a remarkable dysregulation of the inflammatory microenvironment.

Additionally, a total of 13 key inflammatory-immune related molecules, including chemokine-related molecules, interleukins, and CD4 T cell markers such as nuclear factor kappa B subunit 1 (*NFKB1*), were differentially expressed between MPS-I and MPS-II NB (Figure 7 D), strongly indicating that these specific genetic targets may have a high potential for future clinical treatments (Result S3)*.*

### MPS stratification guided therapeutic strategy to facilitate apoptosis and remarkable inhibition of malignant cellular behavior of NB cells

**Figure 8:**
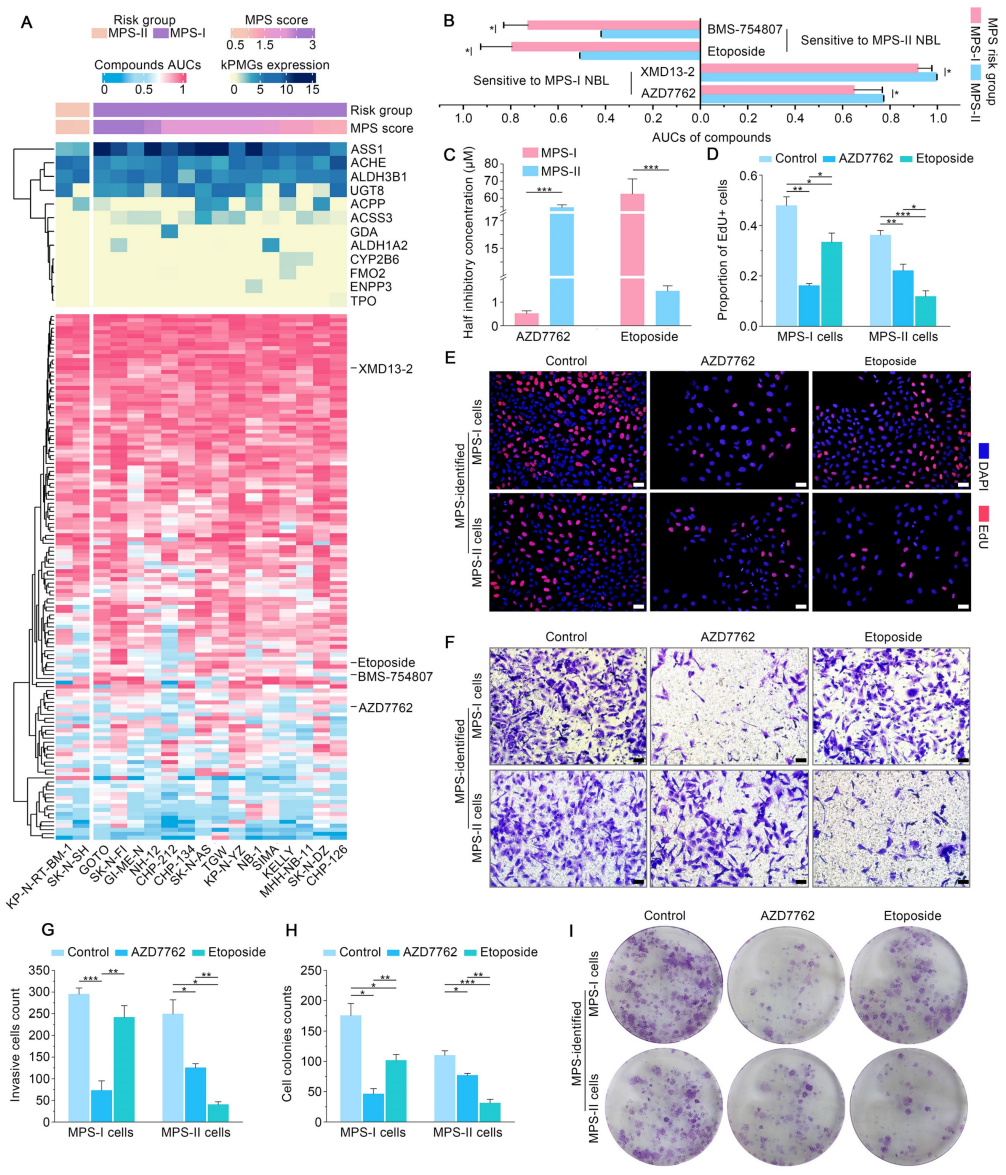
** Individualized treatment prediction and biological validation of MPS-based individualized treatment in the established human NB cells models. (A)** Heatmap exhibited kPMGs expression (these were used for MPS risk-stratification), MPS score and MPS group of each included neuroblastoma CCLs; also shown are the identified therapeutic agents and their AUCs. **(B)** Difference of each identified drug's AUC between MPS-subtyped neuroblastoma. **(C)** IC50 differences of AZD7762 and etoposide between MPS-identified NB subgroups. **(D)** and **(E)**, Results of EdU assays, the scale bar is 50μm. **(F)** and **(H)**, transwell assays measured the invasion capacity of NB cells after treatment. **(G)** and **(I)**, measurement of cell colonies formation capacity of NB cells after treatment.

MPS stratification has a high chance of identifying therapeutic vulnerabilities in MPS-I and MPS-II NB, leading to new diagnostic/prognostic approaches for NB. Resultantly, 17 NB CCLs were stratified with MPS based on kPMG expression, with 2 CCLs (SK-N-SH and KP-N-RT-BM-1) stratified as MPS-II and the remaining as MPS-I (Figure 8 A). These findings were consistent with previously reported studies. For example, in contrast to other NB cell lines, SK-N-SH is an I-type cell that presents an intermediate morphology and mixed properties of N-type (immature) and S-type (non-tumorigenic) cells 50, 51, with a longer doubling time (44 h) and lower tumorigenicity rates after xenotransplantation (32%); KP-N-RT-BM-1 demonstrated a low cloning efficiency of 1% 52. Out of 132 lead candidates, the drug sensitivity results indicated that MPS-II NB cells showed significant sensitivity to etoposide and BMS-754807, whereas MPS-I NB cells were significantly sensitive to AZD7762 and XMD13-2 (Figure 8 A and B). After analyzing the AUC differences and clinical feasibility, the present study strongly suggested AZD7762 as a preferred therapeutic option for treating MPS-I NB (mean AUCs of MPS-I and MPS-II NB: 0.504 vs. 0.795), and etoposide as suitable for treating MPS-II NB (0.771 vs*.* 0.648).

Subsequently, *in vitro* models using SK-N-AS for MPS-I and SK-N-SH for MPS-II NB, were established to validate the MPS-guided therapeutic strategy for NB experimental treatments. Similar to the results of the bioinformatic analysis, AZD7762 and etoposide had significantly lower IC50 values for their corresponding sensitive subtypes, which were 0.52 ± 0.12 μM and 1.46 ± 0.22 μM, respectively (Figure 8 C). These findings align with previously reported studies, demonstrating that the MPS-II counterpart (SK-N-SH) cells were sensitive to etoposide, whereas the MPS-I cells (SK-N-AS) were not 53. Such therapeutic trends were re-confirmed using the EdU incorporation assays which demonstrated that the proportion of proliferating cells (EDU+) was 0.16 ± 0.01 after AZD7762 treatment compared to 0.33 ± 0.04 after etoposide treatment in MPS-I NB cells. In contrast, the proportion of proliferating cells (EDU+) was 0.12 ± 0.02 after MPS-II NB cells were treated with etoposide. This was significantly lower than that after AZD7762 treatment (Figure 8 D and E).

**Figure 9:**
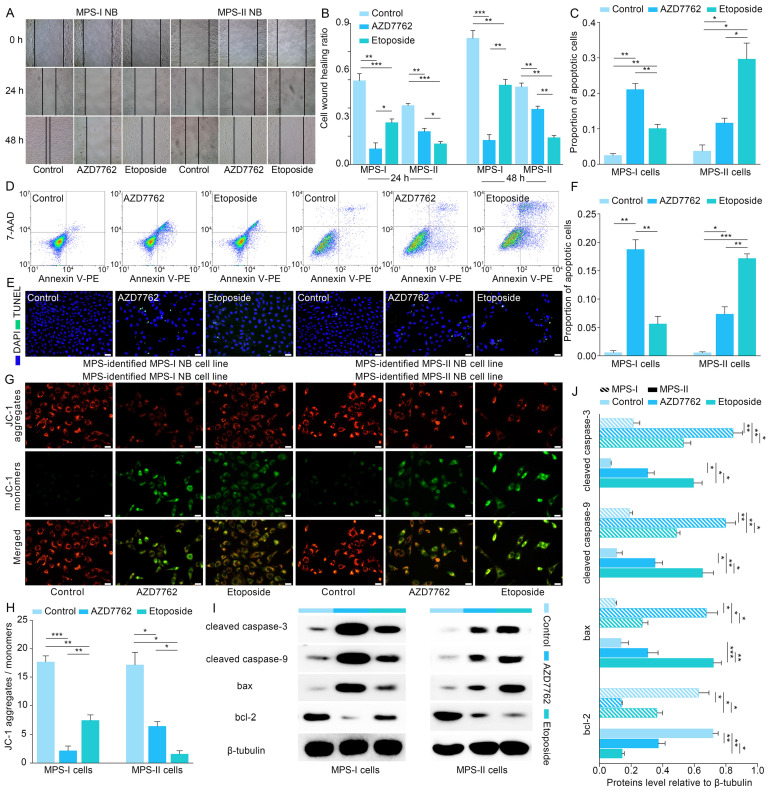
** Therapeutic effects and molecular mechanism of MPS-based individualized treatment in the established human NB cells models. (A)** and **(B)**, cell wound healing assays evaluated the migration ability of NB cells after treatment. **(C)** and **(D)**, flow cytometry revealed the pro-apoptotic effects of MPS-based individualized treatment on their respective sensitive NB subgroup. **(E)** and **(F)**, TUNEL assays confirmed the pro-apoptotic effects of MPS-based individualized treatment against MPS-stratified NB cells. **(G)** and **(H)**, reduction in mitochondrial membrane potential of MPS-I and MPS-II NB cells after individualized treatment. **(I)** and **(J)**, western blotting results demonstrated that the key pro-apoptotic proteins involved in mitochondrial pathway were upregulated, while the anti-apoptotic protein was down-regulated, in the treated MPS-I and MPS-II cells. Scale bars of **(G)** are 25μm, and others are 50μm.

Afterward, the effects of AZD7762 and etoposide on the malignant cellular behavior of NB cells were investigated. As demonstrated in the MPS-I NB group, tumor cell invasion, migration, and the number of colonies formed decreased significantly after AZD7762 treatment, compared to those of both the control and etoposide treatment groups. More specifically, the average number of invasive cells and the number of colonies formed at 14 days after AZD7762 treatment were decreased by 69.83% and 54.42% compared to etoposide treatment, respectively (Figure 8 F to I; Figure 9 A and B). In comparison to the control and AZD7762 groups, the malignant cellular behavior of MPS-II NB cells was significantly inhibited after etoposide treatment. As indicated in Figure 8 F to I and Figure 9 A and B, the average number of invasive cells and the number of colonies formed at 14 days after culture were decreased by 67.85% and 59.31% compared to AZD7762 treatment, respectively. The results of the malignant cellular behavior assays confirmed that the MPS-identified MPS-I and MPS-II NB cells were highly sensitive to AZD7762 and etoposide treatment, respectively.

Furthermore, flow cytometry was used to study the underlying cellular and molecular mechanisms. The results showed that AZD7762 and etoposide treatments significantly induced the apoptosis of MPS-I and MPS-II NB cells, respectively (Figure 9 C and D). The pro-apoptotic effects of AZD7762 and etoposide on their sensitive NB subtypes were verified using the terminal deoxynucleotidyl transferase biotin-dUTP nick end labeling (TUNEL) assay, which demonstrated that the proportion of apoptotic cells was significantly augmented in the AZD7762-treated MPS-I and etoposide-treated MPS-II groups (Figure 9 E and F). Generally, tumor cell apoptosis is mediated by an apoptotic mitochondrial pathway, which is characterized by a decrease or loss of mitochondrial membrane potential. As expected, AZD7762 and etoposide treatments significantly decreased the mitochondrial membrane potential of MPS-I and MPS-II NB cells, respectively (Figure 9 G and H). Since cell necrosis can cause false positive results in the JC-1 test, the DAPI/PI staining was performed to evaluate the necrosis level of the control and treated cells. As shown in Figure S8, although AZD7762 and etoposide increased the necrosis levels of their sensitive NB subtype cells compared to control groups, the necrosis levels of all groups were less than 50%, ensuring the reliability of the JC-1 results. Moreover, key pro-apoptotic proteins involved in the apoptotic mitochondrial pathway, including cleaved CASPASE-9, cleaved CASPASE-3, and BAX, had their expressions significantly upregulated after AZD7762 and etoposide treatments in their respective sensitive NB groups, with a remarkably decreased expression of the anti-apoptotic proteins BCL-2 (Figure 9 I and J). In summary, to achieve more accurate therapeutic effects based on MPS-guided stratification, AZD7762 and etoposide significantly induced the apoptosis of MPS-I and MPS-II NB cells, respectively, through the apoptotic mitochondrial pathway.

### Evaluation of the therapeutic effect of MPS-based therapeutic strategy in the xenografted and metastatic NB mouse models

**Figure 10:**
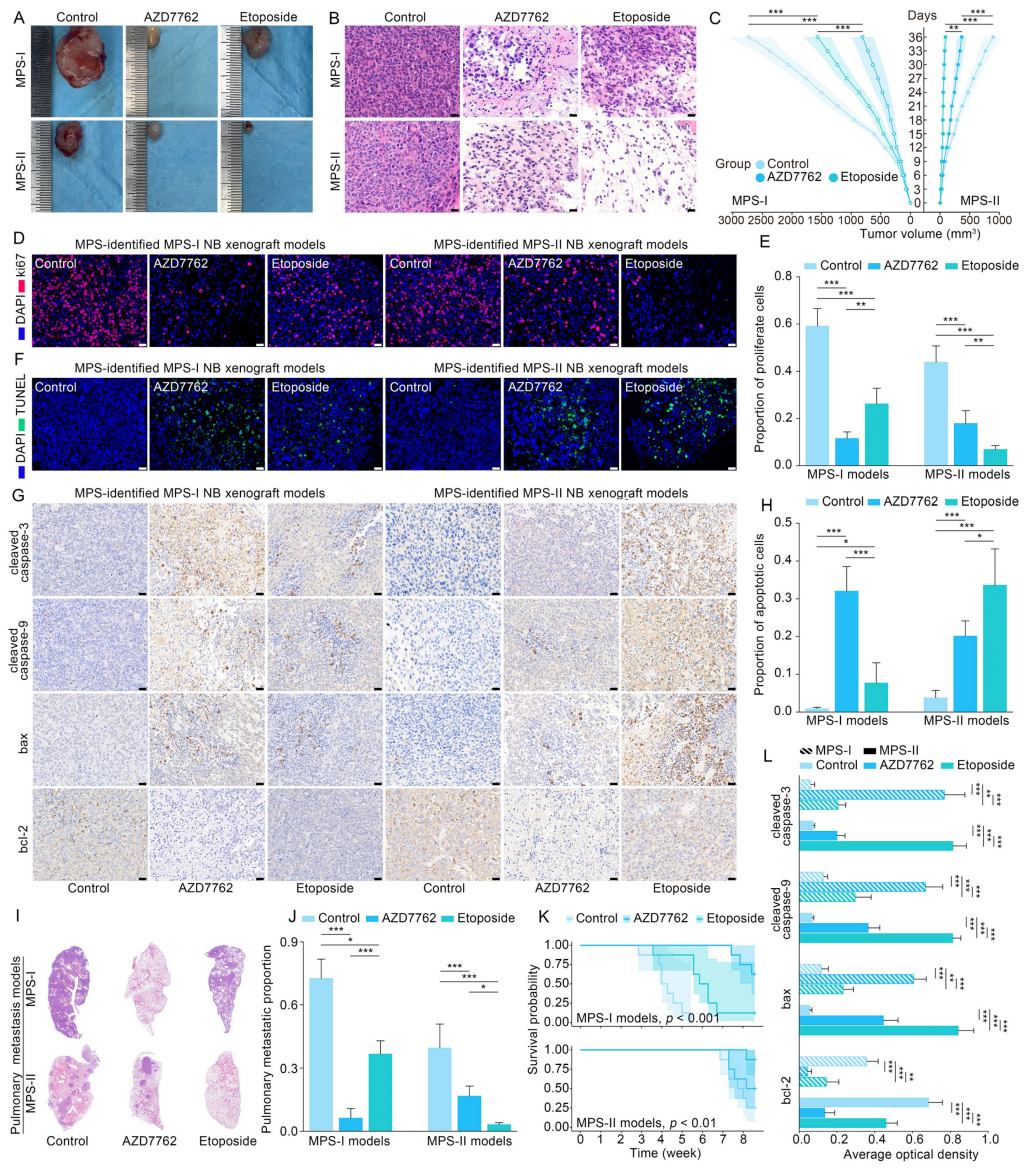
** Validation of therapeutic effects and molecular mechanism of MPS-based individualized treatment in the mouse models. (A)** MPS-I and MPS-II NB xenografts isolated from euthanized nude mouse models exposed to different treatments.** (B)** H&E-staining sections of MPS-I and MPS-II NB xenografts exposed to MPS-based individualized treatment. **(C)** Growth curves of treated MPS-I and MPS-II NB xenografts.** (D)** and **(E)**, results of ki67 staining revealed the *in vivo* proliferation inhibition effects of MPS-based individualized treatment against its sensitive NB subtypes. **(F)** and **(H)**, TUNEL assay confirmed the *in vivo* pro-apoptotic effects of MPS-based individualized treatment against its sensitive NB subtypes. **(G)** and **(L)**, expression of key pro-apoptotic proteins and anti-apoptotic protein involved in mitochondrial pathway in the treated MPS-I and MPS-II NB xenografts. **(I)** and **(J)**, in the established MPS-I and MPS-II NB pulmonary metastasis models, the* in vivo* anti-metastatic effects of MPS-based individualized treatment against its sensitive NB subtypes. **(K)**, overall survival of treated MPS-I and MPS-II NB pulmonary metastasis mouse models. All the scale bars are 50μm.

Based on the promising results of the *in vitro* models, human NB cell lines of SK-N-SH and SK-N-AS were transplanted into nude mice to establish the *in vivo* models to further verify the clinical application potential of MPS-guided risk stratification strategy in the targeted therapy of human NB. Similar to the *in vitro* findings, the proliferation of MPS-I and MPS-II NB xenografts treated with AZD7762 and etoposide, respectively, was remarkably reduced compared to the corresponding control groups (Figure 10 A and C). Furthermore, significant NB xenograft regression was demonstrated in tumor tissues postmortem and confirmed with histopathological examination of hematoxylin and eosin (H&E)-stained sections, which showed reduced tumor growth, more nuclear condensation, and an increased proportion of nuclear fragmentation in the AZD7762-treated MPS-I group, compared to a large number of undifferentiated heteronuclear cells in the corresponding control group (Figure 10 B). Similarly, etoposide treatment significantly induced tumor regression in the MPS-II NB xenograft animals. The antiproliferative activity of AZD7762 and etoposide was re-verified with immunofluorescence staining, which showed that the percentage of proliferative tumor cells (Ki67+) was significantly reduced in the AZD7762-treated MPS-I (0.12 ± 0.03) and etoposide-treated MPS-II (0.07 ± 0.02) groups compared to the other groups (Figure 10 D and E).

Similar to the *in vitro* findings, apoptotic cells were remarkably augmented in the AZD7762-treated MPS-I NB xenografts and etoposide-treated MPS-II tumors (Figure 10 F and H). Accordingly, the immunohistochemistry results also confirmed the significantly higher expression levels of key pro-apoptotic proteins in the mitochondrial pathway, including cleaved caspase-9, cleaved caspase-3, and Bax, with a lower expression level of the key anti-apoptotic protein Bcl-2 after their respective treatments (Figure 10 G and L). These findings indicated that the AZD7762 and etoposide treatments resulted in remarkable suppression of oncogenic proliferation of their respective NB subtypes, with the pro-apoptotic mechanisms of their targeted therapeutic effects mainly involving the mitochondrial pathway.

Pulmonary metastasis remains the end-stage event of NB. To further systematically validate the suppressive effects of the proposed agents on NB metastasis *in vivo*, the pulmonary metastasis models of MPS-I and MPS-II NB were established based on MPS stratification. Consistent with the aforementioned findings, the AZD7762-treated MPS-I NB and etoposide-treated MPS-II NB metastasis samples demonstrated significant suppression of tumor metastasis in full H&E sections, relative to the other groups in which extensive infiltration and metastasis of tumor cells appeared across the lungs (Figure 10 I and J). For example, there were only a few sporadic pulmonary metastases found after AZD7762 treatment in the MPS-I NB metastasis model (0.06 ± 0.05), whereas the control and etoposide groups were up to 0.73 ± 0.09 and 0.37 ± 0.06, respectively. Hence, the overall survival time of AZD7762-treated MPS-I NB and etoposide-treated MPS-II metastasis mice was significantly prolonged, as shown in Figure 10 K. The results of the *in vivo* metastasis preclinical study strongly suggested reliable therapeutic effects of AZD7762 and etoposide in sensitive NB subtypes based on the current MPS stratification strategy. The significant suppression effects on tumor metastasis and elucidated cellular-molecular mechanisms render the present stratification-targeted therapy as one of promising clinical potential.

**Figure 11:**
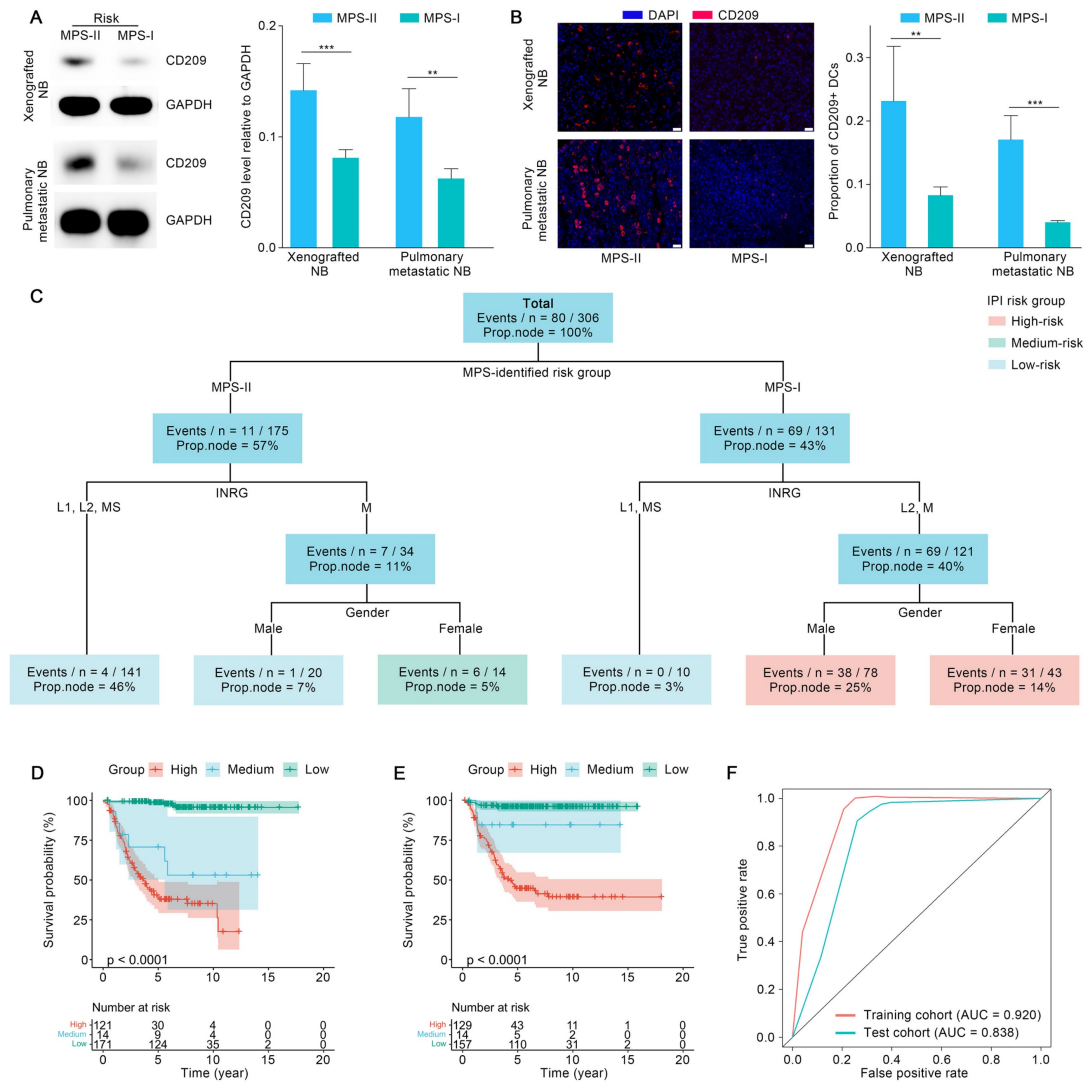
** The biological validation of immunosuppressive microenvironment of MPS-1 samples and the construction and validation of IPI. (A)**, Western blotting quantified the CD209 levels in the MPS-I and MPS-II xenografted and pulmonary metastatic NB mouse models.** (B)**, CD209 staining revealed the infiltration profile of DCs in the xenografted and pulmonary metastatic NB subtypes.** (C)**, The parameters of IPI. **(D)** and **(E)** exhibited the stratification ability of IPI in the training and test cohort. **(F)**, TROC curves confirmed the prediction accuracy of IPI. All the scale bars are 50μm. Abbreviations: Proportion of patients in the node (Prop. Node).

The aforementioned findings showed that infiltration of DCs showed the most significant decrease in MPS-I NB, compared to other immune cells. Thus, infiltration of DCs was re-confirmed using xenografted and metastatic NB mouse models. As shown in Figure 11 A, the protein level of CD209, a biomarker of DCs, was decreased significantly in the samples of mouse models with xenotransplantation and metastatic MPS-I NB. Further confirmed by the immunofluorescence results, the infiltration of CD209+ DCs showed a significantly lower level in MPS-I than in MPS-II samples (Figure 11 B). These findings further confirmed the immunosuppressive microenvironment of MPS-I NB subtypes. Additionally, *ALDH1A2*, a biomarker of retinoic acid resistance, was upregulated in some MPS-I cell lines, including SIMA (FPKM expression: 3.53) and SK-N-FI (1.33), and downregulated in others (mean FPKM expression: 0.001, *p* < 0.001). These findings reaffirmed that some MPS-I NB showed retinoic acid resistance while others did not.

Also, a nomogram was constructed using the MPS to facilitate clinical application (Result S4).

### Integrated prognostic index (IPI) by combining the MPS with clinical parameters

The MPS was combined with key clinical factors to generate an IPI as a practicable tool for potential clinical application after referring to recently published studies 54. The key clinical factors used included the MPS risk group, gender, INRG stratification, and INSS stage. Age and *MYCN* status were waived from the IPI modeling process since these factors have already been included in the INRG stratifications 8. IPI was further validated in the test cohort after being trained using an interpretable machine learning model “survival tree” in the training cohort. The original trained IPI was then pruned to obtain a final precise model, with INSS stages eliminated. The reason might largely be ascribed to the fact that the clinical parameters employed in the INSS stages were mostly identical to those used in the INRG stratification, leading to the multicollinearity of both approaches 8. The final IPI was shown in Figure 11 C. The IPI classified the patients with NB into three risk-level groups according to the survival function values obtained from the training cohort. Survival analyses indicated that the IPI was able to predict the prognosis of NB patients with superior accuracy since the survival probabilities of the IPI-identified high-, medium-, and low-risk groups were extremely significantly different (*p* < 0.001) in the training and test cohorts (Figure 11 D and E). The expected survival rate was highest in the low-risk group, modest in the medium-risk group, and lowest in the high-risk group. As confirmed with the TROC curve, the IPI demonstrated superior robustness and accuracy in terms of 5-year survival prediction. Therefore, the IPI tools integrated key clinical parameters with the MPS, emerging as a novel and reliable prognostic tool for NB risk stratification (Figure 11 F).

### ScRNA-seq validated metabolic reprogramming driving MPS-I NB progression and drug resistance

**Figure 12:**
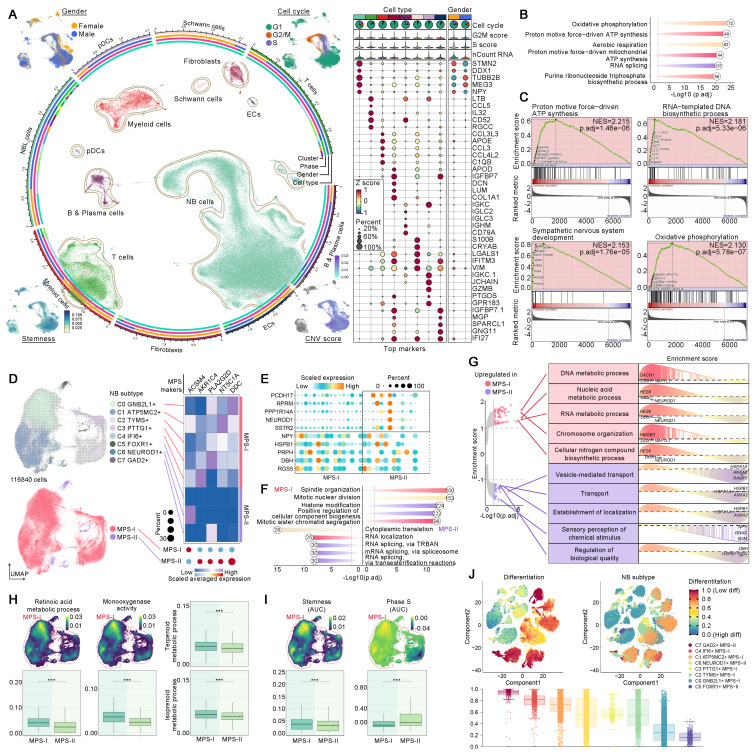
** Metabolic reprogramming-driven alterations of signalling, CNV, stemness, cell cycle, and differentiation. (A)**, The circos plot displayed the annotated cell populations and their clinical feature, cell cycle, CNV, and stemness. The dot plot shown the identified biomarker genes of each cell population.** (B)**, Enrichment analyses exhibited the major biological processes of NB cells.** (C)**, GSEA dissected the upregulated signaling pathways in NB cells. **(D)**, The NB cell subclusters and their MPS stratification. **(E)**, Differential expressed genes between MPS-I and MPS-II NB cells. **(F)**, Results of enrichment analyses upon positive biomarker genes of MPS-I and MPS-II NB cells, they revealed major biological functions of those cells. **(G)**, GSEA revealed dysregulated biological processes within MPS-I and MPS-II NB cells. **(H)**, AUCell algorithm estimated the activities of many resistance pathways within MPS-I and MPS-II NB cells. **(I)**, Quantified stemness and cell cycle phase within MPS-I and MPS-II NB cells. **(J)**, The dissected differentiation potential of each NB subclusters.

ScRNA-seq analyses were conducted to validate aforementioned findings and dissect the essential mechanisms underlying MPS-I NB progression. Quality control and preprocessing of scRNA-seq data were delineated in Result S5, Figure S10 and S11. A total of 153,633 cells from 16 patients with NB were annotated as eight distinct cell populations (Figure 12A and Figure S12A): NB cells, T cells, myeloid cells, fibroblasts, B & Plasma cells, Schwann cells, plasmacytoid dendritic cells (pDCs), and endothelial cells (ECs). The biomarkers of these cell populations exhibited marked divergence (Figure 12A). The gender distribution within the NB scRNA cohort and the proportion of each cell type in individual samples were depicted in Figure 12A and Figure S12B. Predominantly, most NB cells resided in the G1 phase of the cell cycle, with a comparatively short S phase (Figure S12C). Moreover, these NB cells manifested a slightly elevated stemness coupled with significant copy number variations (CNVs). These CNVs extensively spanned the most chromosomal regions, with the regions of chromosome 17 undergoing the most considerable amplifications (Figure S13). Enrichment analysis indicated that the principal biological functions of the NB cells were predominantly associated with metabolic pathways, such as oxidative phosphorylation and ATP synthesis (Figure 12B). More interestingly, GSEA further revealed that, in comparison to other normal cells, NB cells synergistically upregulated these critical metabolic pathways along with DNA synthesis and neurodevelopment (Figure 12C), thereby reaffirming that NB cells underwent remarkable metabolic reprogramming which fueled the progression of NB.

Subsequently, we elucidated that the dysregulated biological processes contributed to unfavorable prognosis of MPS-I NB at a single-cell resolution. Hindered by biases across technological platforms, the MPS model could not be directly applied to the analysis of scRNA-seq data. Thus, these NB cells were further subdivided into eight unique subtypes, with the top five positive biomarkers of MPS-I NB being used to allocate these subtypes to either MPS-I or MPS-II groups. The NB cell subtypes expressing at least two MPS-I biomarkers were identified as MPS-I NB populations (Figure 12D). Interestingly, significant differences in the primary biological functions were observed between MPS-I and MPS-II NB samples, despite all eight subtypes of NB cells upregulating key cell maintainenance and growth-related metabolic pathways (Figure S14). The MPS-I NB cells had remarkable proliferative potential by the overexpression of key genes including *NPY* which is a proliferation-promoting gene, being identified with mitosis as their key biological feature (Figure 12E and F). In contrast, the primary biological functions of MPS-II populations were shown to be relative less directly related to their malignancy, and predominantly involved RNA splicing rather than directly regulating key pathways of proliferation and invasion (Figure 12F). Subsequently, GSEA was utilized to elucidate the dysregulation of BPs of MPS-I and MPS-II NB cells under the whole transcriptomic landscape (Figure 12G). Similarly, signaling pathways of nucleic acid metabolism, biosynthesis of biomolecules, and other BPs closely related to tumor proliferation were significantly upregulated in MPS-I NB cells, while signaling pathways of BPs related to transport and localization showed elevation in MPS-II counterparts. More importantly, scRNA-seq data reaffirmed that many essential metabolic processes associated with drug resistance were upregulated in MPS-I populations. For example, the retinoid metabolic process and monooxygenase activity involved in retinoid resistance, as well as terpenoid metabolism related to terpenoid compound resistance have been shown to support such findings (Figure 12H). These interesting findings significantly deepen the biological insights based on bulk RNA-seq data, indicating that the direct reprogramming of key metabolic pathways fostered malignant progression and drug resistance in MPS-I samples.

### MPS-I NB cells with distinctive developmental lineage showed remarkable potential of proliferation and invasiveness

**Figure 13:**
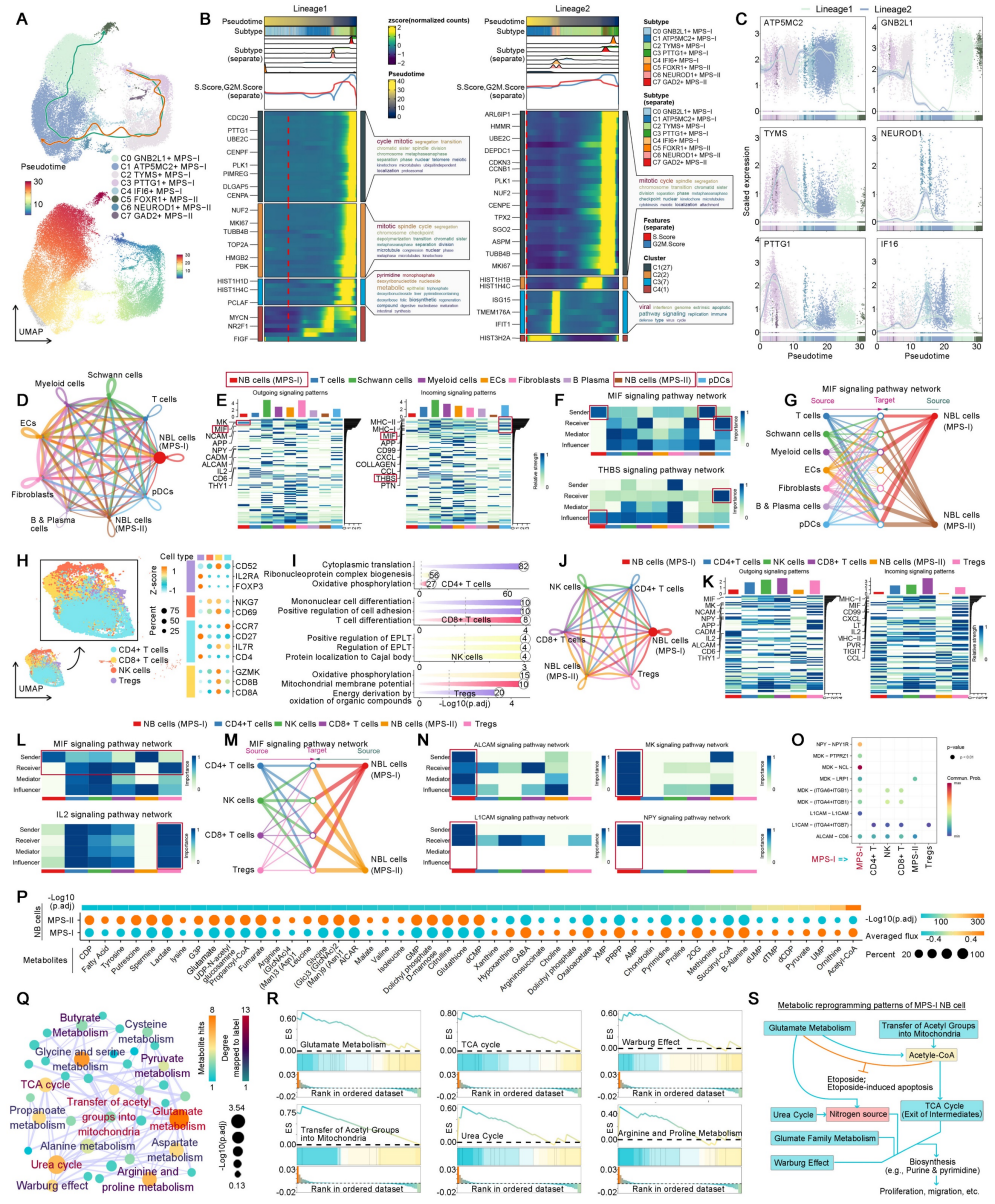
** Developmental lineages tracing, cell-cell communication, and metabolic flux balance analyses of MPS-I and MPS-II NB cells. (A)**, lineage 1 and 2 of NB cells, and their developmental pseudo-time. **(B)**, Developmental modules and their key component genes of lineage 1 and 2. **(C)**, Top genes alternating with NB cell developmental lineage 1 and 2. **(D)**, The global cell communication network and patterns. **(E)**, The incoming and outgoing signaling pathways of each cell population. **(F)**, The sender, receiver, mediator, and influencer of *MIF* and *THBS* signaling pathways. **(G)**, MPS-I and MPS-II NB cells sent *MIF* signaling pathways to other cells. **(H)**, Dimensional reduction and subtypes identification of T cells. The dot plot shown the biomarker to annotate cell subtypes. **(I)**, Enrichment analyses revealed the major biological processes of each cell subtype. **(J)**, The global communication among MPS-I and MPS-II NB cells, CD8+ T cells, CD4+ T cells, NK cells, and Tregs. **(K)**, The incoming and outgoing signaling pathways of each T cell population. **(L)**, The sender, receiver, mediator, and influencer of *MIF* and *IL2* signaling pathways among T cell subtypes. **(M)**, MPS-I and MPS-II NB cells sent *MIF* signaling to CD8+ T cells, CD4+ T cells, NK cells, and Tregs. **(N)**, The top self-communication patterns of MPS-I NB cells. **(O)**, Ligand-receptor analyses of the top self-communication patterns of MPS-I NB cells. **(P)**, Graph Neural Network dissected metabolite flux of MPS-I and MPS-II NB cells. Only those with significant difference between MPS groups were shown. **(Q)**, SMPDB-based metabolic pathways enrichment revealed the reprogrammed metabolic signaling pathways which are upregulated by MPS-I NB. **(R)**, SMPDB-based metabolic pathways GSEA further confirmed the upregulation of identified metabolic signaling pathways. **(S)**, Metabolic reprogramming patterns of MPS-I NB cells, which is derived from metabolic flux balance analyses and enrichment and GSEA analyses. Abbreviations: Establishment of protein localization to telomere (EPLT).

MPS-I NB cells demonstrated augmented stemness with shorter S phase (Figure 12I), which could be mainly ascribed to the high proliferativepotential 55. However, CytoTRACE analysis revealed that various subpopulations within MPS-I NB cells showed significantly divergent potentials of differentiation (Figure 12J), suggesting that MPS-I NB cells might undergo specific developmental trajectories and their malignant characteristics could not be fully attributed to the under-differentiated or undifferentiated state (Figure 12J). Consequently, pseudotemporal analysis was employed to trace the developmental trajectories of NB cells showing two partially overlapping lineages (Figure 13A), both of which originated from C3 MPS-I NB cells and diverged at C1 MPS-II NB cells. Lineage 1 underwent differentiation towards C5 MPS-II NB cells, embodying a classical developmental trajectory from low to high differentiation. Clinically, a minority of NB cells might spontaneously differentiate, mature, and ultimately regress, which highly mirrored the high-differentiation state leading to a less malignant phenotype (MPS-II) as observed in lineage 1 developmental trajectory.

Intriguingly, lineage 2 was exclusive to MPS-I NB cells whose destination turned to be C1 MPS-II NB cells with lower differentiation potential (Figure 12I and 13A), representing a typical dedifferentiation lineage development. Although the major developmental modules of lineages 1 and 2 were functionally similar, encompassing metabolism, mitosis, and cell cycle (Figure 13B), more profound gene-lineage tracking data delineated the unique characteristics of lineage 2 (Figure 13C). Top genes alternating with NB cell development included *ATP5MC2*, *TYMS*, *PTTG1*, *GNB2L1*, *NEUROD1*, and *IF16*. For example, *ATP5MC2* directly regulates ATP synthesis, *TYMS* is involved in DNA synthesis 56, and *PTTG1* accelerates the G1/S phase transition of NB cells 57; these three oncogenes were upregulated during the early to middle stages of development (lineage 2) and downregulated as lineage 1 developed. Notably, low expression of *GNB2L1* has been reported to be associated with chemotherapy resistance in several cancers 58 and moderate expression of *GNB2L1* was found in the developmental origins of NB cells. However, expression of *GNB2L1* was completely suppressed in the destination cells of lineage 2, but significantly increased during the development of lineage 1, thereby indicating that lineage 2 development might potentially confer MPS-I NB cells with chemotherapy resistance. It has been reported that *NEUROD1* promoted tumorigenesis, proliferation and migration of NB cells and *IF16* strongly inhibited cell apoptosis 59, 60. In the present study, both oncogenes were upregulated during the development of lineage 2 but consistently downregulated in lineage 1. Collectively, the unique developmental lineage (lineage 2) of MPS-I NB cells showed more marked potential of drug-resistance, proliferation, migration, and anti-apoptosis, whereas lineage 1 led to the more benign phenotype of MPS-II NB.

### NB-immune cell crosstalk facilitated immune evasion by suppressing DCs and T cells in NB development

We previously evidenced that the immunosuppressive microenvironment would facilitate the malignant progression of MPS-I NB. Thus, the crosstalk between MPS-I/II NB cells and immune cells has been elucidated in the present study using scRNA-seq. The global cell communication network and patterns are depicted in Figure 13 D and E. In MPS-I NB samples, DCs have been primarily focused due to their significant downregulation which has been corroborated in our preclinical models. The principal signals received by pDCs were identified as *MIF* and *THBS* signaling pathways (Figure 13 E to G). *MIF* signaling pathway, predominantly originating from MPS-I/II NB cells, promotes immune evasion of tumor cells by inhibiting infiltration, maturation, and antigen presentation of DCs 61. Moreover, *THBS* signaling pathway acts as a bridge between the tumor and immune cells, promoting tumor progression and immune escape 62. These previous studies aligned with our aforementioned findings in vivo (Figure 11), highlighting *MIF* and *THBS* signaling pathways served as the potential mediators leading to DC suppression in MPS-I NB cells.

T cells, known as the most prominent cytotoxic cells, directly mediate the elimination of tumor cells. However, in the specific tumor-microenvironment, Tregs differentiated from CD4+ T cells markedly promote immunosuppression and immune escape 63. Therefore, the intercellular communication between NB and T cell subpopulations has been focused on. Using various biomarkers, the original T cells were subdivided into CD8+, CD4+, NK cells, and Tregs (Figure 13 H). Interestingly, the primary biological pathway of CD8+ T cells was differentiation (Figure 13 I), indicating significant disruption of their normal biological function in NB samples. The global communication pattern between MPS-I/II NB cells and T cell subgroups was detailed in Figure 13 J and K, and *MIF* and *IL2* signaling pathways were highlighted. Previous studies have attested that *MIF* signaling pathway is essential for the immunosuppressive activity of Tregs by promoting generation and proliferation of Tregs through upregulation of *IL2* expression 61, 64. Tumor-mediated *MIF* signaling pathway also inhibits NK cell activity, aiding in the evasion of NB cells from NK cell-mediated cytolysis 61. Moreover, *MIF* suppresses T cell activation and induces T cell death through INF-alpha signaling pathway 61. Additionally, *IL2* signaling pathway induces Foxp3 expression and supports the immunosuppressive function of Tregs 65. Notably, the present data showed that MPS-I/II NB cells consistently interacted with entire T cell subgroups through *MIF* signaling pathway (Figure 13 L and M), and communicated with Tregs by *IL2* signaling pathway. It indicated that MIF and IL2 signaling pathways may be the potential mediators of downregulated T cell infiltration that we evidenced in clinical NB samples (Figure 6).

### Self-communication promoted the malignancy of MPS-I NB

MPS-I NB cells exhibited a distinctive characteristic of self-communication which markedly accelerated their malignant progression (Figure 13 N). In the previous study, *ALCAM* signaling pathway was found to promote proliferation, migration, and immune evasion of NB cells 66. *L1CAM*, overexpressed in various tumors including NB in the present study, was reported to markedly facilitate cell adhesion, proliferation, and migration 67-69; thus, therapies of CAR T cells targeting *L1CAM* have been investigated in the stage of clinical test NB 69. *MK* signaling pathway, via the Notch pathway, has been shown to be pivotal in promoting tumorigenesis and progression of NB 70. *NPY* has also been shown to stimulate cellular motility and invasiveness, as well as proliferation of NB 71, acting as a chemotactic factor for NB cells, inducing angiogenesis and leading to an adverse prognosis 72. Compared to MPS-II NB, MPS-I NB cells demonstrated significant capabilities of self-sending and self-receiving these four tumorigenic signaling pathways (Figure 13 N), consequently showing more pronounced invasive and proliferative characteristics. Moreover, ligand-receptor analyses further indicated that MPS-I NB cells predominantly regulated signaling pathways of *NPY*, *MK*, *L1CAM*, and *ALCAM* through the interactions of *NPY*-*NPY1R*, *MDK*-*MDKNL*, *L1CAM*-*L1CAM*, and *ALCAM*-*CD6*, respectively (Figure 13 O).

### The etoposide resistance and malignant features of MPS-I NB are directly driven by metabolic reprogramming

Previously, we confirmed that unique metabolic reprogramming in MPS-I NB cells mediated drug resistance, the immunosuppressive microenvironment, and malignant progression. Consequently, the graph neural network was employed to directly decipher the flux of 70 key metabolites at single-cell level, yielding deeper insights into the metabolic reprogramming of MPS-I NB samples. Notably, 24 metabolites were significantly upregulated, and 25 metabolites were downregulated in MPS-I NB cells, which confirmed that approximately 71.43% of the metabolites in the present study were dysregulated while reaffirming that substantial metabolic reprogramming occurred in these cells (Figure 13 P). Intriguingly, hypergeometric enrichment analysis based on Small Molecule Pathway Database (SMPDB) for positive biomarkers of MPS-I NB cells revealed that the most significantly upregulated metabolic activities included glutamate metabolism, urea cycle, transfer of acetyl groups into mitochondria, TCA cycle, purine & pyrimidine metabolism, and related glutamate family metabolic pathways (Figure 13 Q). Our previous findings demonstrated that etoposide-induced mitochondrial damage, such as alterations in membrane potential, led to apoptosis of MPS-II NB cells through a mitochondria-dependent pathway (Figure 9). Besides, etoposide also inhibited mitochondrial respiratory chain complex I and induced ROS production 73. However, MPS-I NB cells exhibited etoposide resistance (Figure 9), most likely due to their addiction to glutamate metabolism and acetyl-CoA. Reported studies have shown that highly active glutamate metabolism aided in the mitochondrial elimination of excess ROS, protected the mitochondrial membrane, and prevented the activation of mitochondria-dependent apoptotic pathways, thereby conferring etoposide resistance on NB cells with glutamate metabolism-addiction 73. Glutamine deprivation could make NB cells re-sensitize to etoposide with recovery of apoptotic efficiency 73. It has been reported that the generation of acetyl CoA is highly dependent on glutamate and directly occurs through reducing carboxylation 74. Of note, Acetyl CoA has been shown to directly resist etoposide induced apoptosis in a previous study 75. Interestingly, in the present study, acetyl-CoA, transfer of acetyl groups into mitochondria, and glutamate metabolism were found to be upregulated in MPS-I NB cells (Figure 13 Q and R), which indicated such synergistic mechanisms led to the etoposide resistance (Figure 13 S).

In the proliferating cells, the tricarboxylic acid (TCA) cycle has divergent patterns wherein intermediate products could exit the cycle to support various biosynthetic pathways 74, 76. Diverging from normal cells, tumor cells could obtain nitrogen sources via urea cycle, to bolster their robust demands on metabolism and biomass synthesis 77. Basically, there are two main pathways for *de novo* synthesis of pyrimidine and the most general pathway is to use glutamate as the raw material. However, in the proliferating cells, an alternative approach is to utilize inorganic ammonia, a byproduct of glutamate metabolism, as materials for pyrimidine production. Herein, mitochondrial Carbamoyl Phosphate Synthetase I (CPS1), an initiatory enzyme of the urea cycle, catalyzes the synthesis of carbamoyl phosphate 77. Urea cycle also essentially supplies building blocks for synthesis of proteins and nucleic acids 77. Additionally, amino acid metabolism within the 'glutamate family' alongside the Warburg effect are indispensable for cellular proliferation and biomass synthesis 78. In the present study, the remarkable upregulation of glutamate metabolism, the urea cycle, the TCA cycle, and related glutamate family metabolic pathways in MPS-I NB cells illustrated that these cells adapt various pathways to match their augmented demands on metabolism, which included the utilization of TCA cycle intermediates and the recycling of inorganic nitrogen (Figure 13 Q and R). Such adaptive patterns underlined the highly malignant characteristics of MPS-I NB cells, such as significantly increased proliferation and metastatic potential (Figure 13 S).

### LC-MS/MS quantified metabolomics analyses of human MPS-I and MPS-I NB cell lines

**Figure 14:**
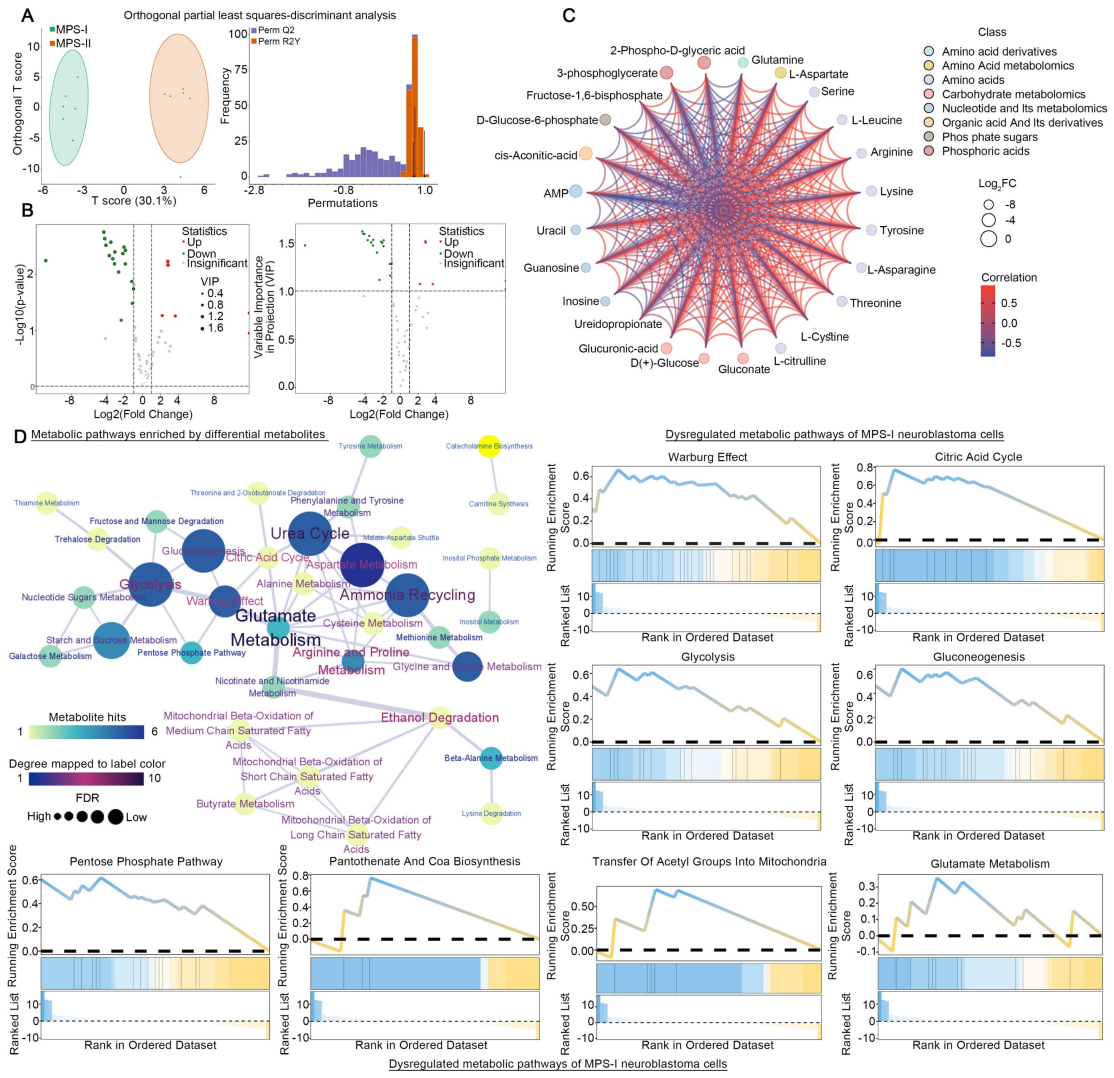
** LC-MS/MS quantified metabolomics analyses of human MPS-I and MPS-I NB cell lines. (A)**, Orthogonal partial least squares-discriminant analysis.** (B)**, Identified differential metabolites between MPS-I and MPS-II NB cells.** (C)**, Correlations among differential metabolites. **(D),** SMPDB-based metabolic pathways enrichment analyses, and GSEA for measuring dysregulated metabolic pathways within different groups.

Following the elucidation of distinct metabolic reprogramming patterns in MPS-I and MPS-II NB cells via scRNA-seq, we directly employed LC-MS/MS quantified metabolomics analysis on previously identified human NB cell lines subtyped as MPS-I and MPS-II, to further validate the scRNA-seq results and investigate their reliance on different fuels. Twelve cell line samples were included in the metabolomics analysis, and MPS-I and MPS-II samples were found to exhibit significantly different energy metabolism patterns (Figure 14 A). Among all metabolites related to energy metabolism, 62 metabolites were detected, with six upregulated and 18 downregulated in MPS-I NB cell lines (FDR < 0.05, VIP >1, and |log2Fold-Change| >1; Figure 14 B and S15). There were significant interactions among these differential metabolites (Kendall p < 0.05; Figure 14 C). Hypergeometric enrichment analysis based on the SMPDB database mirrored the scRNA-seq results and additionally revealed some pivotal metabolic reprogramming patterns (Figure 14 D). Similarly, differential metabolites primarily regulated key metabolic pathways such as glutamate metabolism, the urea cycle, the TCA cycle, glycolysis, and the Warburg effect. Furthermore, pathways including ammonia recycling, aspartate metabolism, starch and sucrose metabolism, glycine and serine metabolism, gluconeogenesis, and the pentose phosphate pathway were also identified to be significantly regulated by differential metabolites. Encouragingly, such findings indicated that there were markedly distinct metabolic reprogramming patterns, particularly involved in processes like glucose utilization and recycling, ammonia utilization, amino acid metabolism, and nucleic acid metabolism, occurred between MPS-I and MPS-II cell groups. GSEA analysis further corroborated the scRNA-seq results (Figure 14 D), showing significant upregulation of pathways including glutamate metabolism, the urea cycle, the TCA cycle, glycolysis, synthesis of acetyl-CoA and its transport to the mitochondria, as well as the Warburg effect in MPS-I NB cell lines.

## Discussion

The present study pioneered a precise oncological strategy based on molecular characteristics of NB metabolic reprogramming to achieve accurate prognostic risk stratification of patients with NB and offered more reasonable individualized treatment different from the empirical therapies applied in the current clinical practice. Thus, the clinical oncologist would be able to pre-stratify the patients of NB, and optimize the potential therapy regime based on their MPS subtype, while maximally avoiding side effects caused by blind or empirical treatments.

Currently, the clinical treatments of NB face huge challenges since the patients cannot fully benefit from empirical chemotherapy currently practised 3, 79. Despite the advancements made by the currently used approaches including *MYCN* status, INSS stage, and INRG stratification, the inherent limitations including insufficient accuracy owing to missing genetic information, hindered these approaches from guiding more precise and effective interventions 2. Herein, based on the exploration of the molecular mechanisms of NB metabolic reprogramming, the MPS was proposed to update the guidance of individualized treatments based on the risk stratification of patients with NB as a promising biomarker. In benchmark comparisons, the MPS demonstrated a more accurate prognosis prediction power than *MYCN* status, INSS stage, and INRG stratification (NRI: 44.4%, 39.8%, and 18.2%, respectively; all *p* < 0.05)); moreover, initial classification of NB patients with certain approach like INRG stratification can be further subdivided by the present MPS. Encouragingly, the IPI obtained by MPS achieved more accurate prediction power after integration with clinical parameters.

Clinically, the lack of robust prognostic markers to identify subgroups of patients who might be sensitive to certain agents has greatly hindered the development of targeted therapies for patients with NB 80. Sequencing technology has fostered new targeted therapeutic strategies for certain solid tumors, such as liver cancer with TP53 mutation 30. However, most of such studies were limited by the lack of further well-designed biological verifications, thereby losing their translational potential. In the present study, a highly promising treatment strategy based on MPS stratification was developed and validated in preclinical models to significantly improve the current targeted therapeutic options in patients with NB. Etoposide, an agent that induces DNA damage after binding and inhibiting topoisomerase II, has been widely employed to treat high-risk NB clinically 81. Unfortunately, many clinical high-risk NBs have shown resistance to etoposide, which greatly hinders its clinical efficiency 81, 82. Besides, some high-risk NB cell lines, such as SK-N-AS, also insensitive to etoposide 53. These observations call for further development of more effective drug candidates and refined identification of etoposide-sensitive NB subtypes. AZD7762, an inhibitor of checkpoint kinase (Chk) 1 and 2, has been reported to inhibit the proliferation and invasion of breast cancer cells partially by pro-apoptosis 83, 84. AZD7762 has also increased sensitivity of certain cancer subjects to DNA-damaging agents in clinical trials 85. In the present study, AZD7762 and etoposide have shown highly curative effectiveness on MPS-I and MPS-II NB, respectively. Notably, corresponding *in vitro* and *in vivo* tests further confirmed the significant inhibition of AZD7762 and etoposide on the proliferation, migration, colony formation, and pulmonary metastasis of MPS-I and MPS-II NB cells, respectively. Both AZD7762 and etoposide significantly induced the apoptosis of NB cells by activating the mitochondria-dependent apoptosis pathway. Such encouraging curative effects significantly prolonged the survival period of tumor-bearing mice. More importantly, etoposide has been evidenced to be effective only in treating MPS-II NB in the present study, despite being recommended as an empirical medicament applied in the current clinical treatment for NB. As the present data showed, MPS-I NB cells are less sensitive to etoposide despite its partial inhibitory efficiency on the proliferation of MPS-I NB cells. The present findings would significantly update the clinical indications of etoposide which should be used as the preferred drug for MPS-II NB rather than an empirical medicament for treating all NB, since the side effects of etoposide applied in treating MPS-I NB may exceed treatment benefits.

In summary, the present evidence confirmed that personalized drug therapy after specialized selection based on MPS stratification could significantly inhibit the progression of certain MPS NB subtypes. Encouragingly, the present study strongly suggested that AZD7762 and etoposide could be applied as drugs of choice for treating MPS-I and MPS-II, respectively.

Reprogramming of the metabolic pathways is required for tumor development and malignant transformation in the stage of tumor progression or relapse. For example, increased lipid metabolism strengthens bioenergy acquisition and signal transduction, particularly during biological membrane construction 86; altered nucleotide and glucose utilization indisputably support excessive cell proliferation 12, 15, 87, pentose phosphate pathway and redox balance 88, 89. Interestingly, all of these aforementioned critical metabolic pathways were remarkably upregulated in the MPS-I NB, in contrast to the MPS-II NB samples. Notably, our study is the first to show that MPS-I NB demonstrates distinct metabolic reprogramming patterns, such a synergistic reprogramming of metabolic signaling pathways facilitates malignant cell behaviors including proliferation and invasion. Contrary to MPS-II NB cells characterized predominantly by RNA splicing, MPS-I NB cells show significant upregulation of pathways closely related to malignant proliferation including mitosis and synthesis of biomacromolecules like DNAs and RNAs 90. Single-cell metabolic flux analysis of MPS-I NB samples further deciphered the metabolic mechanisms underlying the significant cell proliferation activity, which was supported by metabolic reprogramming results. While glutamate is the most important raw material and the principal nitrogen source for the synthesis of purines and pyrimidines 77, MPS-I NB cells exhibit addiction to or metabolic dependency on glutamate metabolism. Glutamate metabolism alone is insufficient to support the hyperactive biosynthetic activities of MPS-I NB cells, indicating additional nitrogen sources are in need. Basically, inorganic nitrogen, a cytotoxic byproduct of glutamate metabolism, cannot be physiologically utilized by normal cells 77. However, the rate-limiting enzyme CPS1 in urea cycles that occurred in the proliferation of glutamate-addicted cells can utilize inorganic nitrogen as a new nitrogen source that serves as building blocks of purine and pyrimidine synthesis 77. Additionally, the TCA cycles that occurred in proliferating cells showed markedly distinct patterns with intermediate products exiting the cycle, to support the branching metabolic pathways including biomass synthesis 74, 76. Our study indicates that significant upregulation of amino acid metabolism occurred in the urea cycle, TCA cycle, glutamate family, and Warburg effect in MPS-I NB samples. Such results are also strongly supported by published evidence mentioned above. These key metabolic pathways, in concert with glutamate metabolism, synergistically match the excessive metabolic demands of MPS-I NB cells, fundamentally contributing to their malignant characteristics and poor prognosis.

Developmental lineage determines the fate of NB cells. For example, NB cells in terminal differentiation stage generally demonstrate relatively benign phenotypes 91, while the dedifferentiated NB counterparts are of poor prognosis. Specifically, highly malignant NB originates in adrenal neuroblastoma at early stages of differentiation trajectory, while relatively benign NB counterparts originate in lineage at late developmental stage 91. Of note, the present study would be pioneering in discovering that such two unique NB developmental lineages determine the MPS stratification. Remaining at the relatively terminal phase of development, MPS-II NB cells exhibit remarkedly reduced differentiation potential, thereby indicating comparatively benign prognosis. In stark contrast, MPS-I NB cells demonstrate a distinctly divergent developmental trajectory with pronounced tendency towards dedifferentiation and progressively upregulated expression of oncogenes including *ATP5MC2*, *TYMS* 56, *PTTG1* 57, *NEUROD1* 59, and *IFI16* 60. Such genetic mechanisms underlie the highly malignant phenotypes of MPS-I NB samples characterized by the excessive proliferation, instinct invasion potentials, and apoptosis resistance.

Spanning the entire lifecycle of tumors, self-communication occurring during intracrine and autocrine is crucial for differentiation, proliferation and morphogenesis of tumor cells 92. For example, NB autonomously secretes *FGF1* to evade p53-dependent apoptosis 93. Compared to MPS-II NB counterparts, the MPS-I NB samples uniquely identified in the present study exhibit a pronounced self-communication pattern which significantly sends and receives key signals including *ALCAM*, *L1CAM*, *MK*, and *NPY*. All of these signaling pathways have demonstrated to directly promote the tumorigenesis, proliferation, migration, and invasion of NB. Notably, acting as a chemoattractant with ability to induce angiogenesis, *NPY* can not only stimulate the motility and invasiveness of NB cells but also contribute to the poor prognosis of MPS-I NB 71, 72. The present data further suggested that the significantly aberrant activities of intracrine and autocrine underlay the malignant behavior of NB cells.

Intrinsic drug resistance (IDR) remains one of the most considerable risk factors contributing to the survival prediction of clinical NB. Recently, a therapeutic regimen of differentiation induction using retinoids or retinoic acid derivatives emerged as a highly promising treatment for clinical NB 7. However, the differentiation induction therapeutics currently using retinoids turn to failure due to IDR, which results in significant tumor relapse and mortality in high-risk NB patients 2, 94. Recent studies revealed that signaling pathways involved in retinoid metabolism and cytochrome P450-associated pathways play key roles in the molecular mechanisms involved in IDR to retinoids and retinoic acid derivatives 43, 45. Moreover, the upregulation of *ALDH1A2* was reported to significantly initiate IDR to retinoids 46. In the present study, both findings at bulk and single cell levels further revealed that core MGs including *ALDH1A2* significantly upregulated multiple signaling pathways involved in retinoid metabolism, and *AL1A2*, which is an *ALDH1A2*-coded protein, had a high binding affinity to retinoic acid derivatives. These findings updated the genetic mechanisms of IDR to retinoids and retinoic acid derivatives in MPS-I NB. Crucially, the present study firstly demonstrates that MPS-I NB exhibited resistance to mitochondrial-dependent apoptosis induced by etoposide (Figure 9). Such underlined mechanisms might be highly ascribed to the glutamate metabolism and acetyl CoA addition, which were supported by the single-cell metabolic flux analysis (Figure 13 Q to S). Previous studies have shown that glutamate metabolism aided in clearance of ROS from mitochondria, preserved the integrity of mitochondrial membrane and prevented the pathways of mitochondrial-dependent apoptosis from activating, thereby conferring NB cells with resistance to etoposide 73. Moreover, it has been reported that acetyl-CoA production was highly dependent on glutamate, and an excess of acetyl-CoA could counteract etoposide-induced apoptosis in tumors addicted to glutamate metabolism 74, 75. Notably, the present data shows that various pathways highly associated with drug resistance, including terpenoid metabolism, have been found to be upregulated in the MPS-I NB cells, whose unique developmental lineage significantly suppresses the expression of GNB2L1, while GNB2L1 has been reported to be the chemotherapy-sensitivity gene 58. These findings collectively substantiate that MPS-I NB acquires drug resistance to multiple agents like retinoids and etoposide through metabolic reprogramming, thereby supporting its malignant phenotype and securing the competitive survival advantage during its malignant development.

The dynamic and mutualistic interactions between cellular and extracellular components form the specific tumor microenvironment which directs tumor progression, chemoresistance, immune escape, and metastasis of solid tumors like NB 32, 95. *MIF* signaling pathway found in tumor metabolism has been substantiated to inhibit various immune-cell activities including cellular infiltration, maturation, and antigen presentation 61. During NB development, *MIF* signaling pathway intimately mediates the suppression of macrophage migration and T-cell responses 96, 97. Our study further discovered that both MPS-I and MPS-II NB inhibited infiltration of DCs through *MIF* signaling pathway, in addition to the inhibition of macrophages and T cells, thereby confirming the broader immunosuppressive effects of *MIF* signaling pathway active in NB metabolism. Such findings strongly indicate that an "immune desert" featured in tumor microenvironment is consistently presented in the NB samples, and such characteristic tendency is more deeply exemplified in MPS-I NB. Compared with MPS-II, the MPS-I NB samples showed a remarkably immunosuppressive tumor microenvironment in which the infiltration abundances of 14 immune effector cells were all downregulated. Among these major immune effector cells, DCs have been previously confirmed to play a vital role in antitumor immunity, which includes promoting immunity or tolerance by sampling and presenting antigens to T cells, as well as exerting immunomodulation via intercellular signals and cytokines 98, 99. Unfortunately, multiple cytokines were found to reduce the infiltration abundance of DCs, inhibiting their antitumor activity in an immunosuppressive microenvironment of malignant tumors 100. Interestingly, this study found that the infiltration abundance of DCs in MPS-I NB was maximally downregulated, which was confirmed by the log_2_FC outcome (Figure 6). Additionally, the *THBS* signaling pathway, acting as a pivotal media facilitating the crosstalk between tumor and immune cells, also plays a critical role in promoting tumor malignancy progression and immune escape 62. The present study shows that *THBS* signaling pathway has been significantly upregulated to regulate the immune activities of DCs in MPS-I NB samples, further strongly indicating that both *MIF* and *THBS* signaling pathways have simultaneously contributed to the suppression of DCs' function and escape of immune surveillance in MPS-I NB samples. In light of such findings, a highly promising anti-tumor immunotherapeutic approach might most likely be developed from the DC-targeted strategies.

Notably, recently reported studies suggest that the inflammatory microenvironment contributes greatly to the malignant development of tumors. Acute inflammation tends to exert obvious antitumor effects, while chronic inflammation evokes long-term infiltration of inflammatory cells and highly contributes to the malignant progression of cancer 101, 102. As revealed in the present study, in contrast to significant upregulation of chronic inflammatory-associated signaling pathways, acute inflammation was significantly downregulated in MPS-I NB. For example, respiratory burst, also known as reactive oxygen species (ROS) burst, involves neutrophil infiltration and has been generally induced significantly by acute inflammation 103. ROS have traditionally been regarded as a tumorigenesis promoter via DNA damage and oncogene activation 104. However, recent studies indicated that ROS play a dual role in the occurrence and progression of tumors because excessive ROS was reported to inhibit the growth of certain tumors. Additionally, elimination of ROS facilitates the survival of tumor cells within the extracellular matrix in pancreatic and breast cancers 105, 106. Furthermore, the nano-radiosensitizer-induced ROS burst exerted significant antitumor effects in breast cancer 107. The present study found that "negative regulation of respiratory burst" was dramatically upregulated among the inflammatory signaling pathways triggered in MPS-I NB, while neutrophil infiltration was remarkably downregulated (Figure 6 A and B). Collectively, the specific inflammatory microenvironment, such as the synergistic effects of downregulated infiltration abundance of neutrophils and reduced expression of ROS burst signaling pathway, greatly contributed to the malignant progression of MPS-I NB. Of note, an immune and inflammatory molecular target that may help develop novel treatments of NB was also identified (Discussion S1).

The present study is limited by the inherent drawbacks of retrospective cohort studies, despite the large sample sizes and careful validations of the present results. According to the proposed MPS strategy, large and well-planned prospective clinical studies are needed to further identify and improve the relevant proposal with personalized precision medication for NB patients, especially those MPS-subtyped NB subjects with drug resistance or inferior drug sensitivity. Some critical metabolic alterations in MPS-I NB, including glutamate metabolism, warrant further exploration with well-designed studies using specific metabolic inhibitors. Additionally, given the lack of MPS-I NB cell lines with *MYCN* amplification, such as the Kelly cell line, larger-scale biological validation experiments need be carried out once such cell lines are available by domestic biotechnology suppliers. Besides, some identified hazardous PMGs like *ALDH3B1* and *ASS1* exhibited seemingly paradoxical expression patterns (lower expression compared to control tissues), which hints an interesting phenomenon that the altered key interaction behind these gene networks may lead to compensatory increases or decreases in expression of certain genes active in neuroblastoma. These genes and their regulatory network will be explored in our future study.

## Conclusions

In light of the elaborated investigation into the molecular mechanisms of NB, the present study pioneered a novel and more accurate metabolic reprogramming-based prognostic signature (MPS) and developed a comprehensive therapeutic strategy to guide targeted medication for corresponding stratified NB subjects. More importantly, the therapeutic effects of the MPS-based personalized treatment were further systematically validated in pre-clinical models. Notably, the adverse prognosis of MPS-I NB was ascribed to multiple factors, including multidrug resistance, immunosuppressive, and tumor-promoting inflammatory microenvironments. Immunologically, MPS-I NB suppressed immune cells via *MIF* and *THBS* signaling pathways. Based on analysis of energy metabolomics, it has been suggested that the malignant proliferation of MPS-I NB cells was remarkably supported by reprogrammed glutamate metabolism, tricarboxylic acid cycle, urea cycle, etc. Furthermore, in contrast to the MPS-II counterpart, MPS-I NB manifested a distinct tumor-promoting developmental lineage and self-communication patterns, as evidenced by enhanced oncogenic signaling pathways activated with development and self-communication. Based on the updated data of transcriptomics and energy metabolomics, such encouraging findings will definitely enable the clinical oncologist to use more effective and personalized medications to counteract the progression of NB while avoiding the adverse consequences caused by blind or empirical intensive treatments, offering significantly promising opportunities for the clinical treatment of NB.

## Supplementary Material

Supplementary methods, figures and tables.

## Abbreviations

AIC: Akaike information criterion
AUC: Area-Under-Curve
BP: describing biological process
CC: cellular component
CCLs: cancer cell lines
C-index: index of concordance
DCA curve: decision curve analysis
DETFs: differentially expressed TFs
ESTIMATE: Estimation of stromal and immune cells in malignant tumor tissues using expression data
FPKM: Fragments Per Kilobase per Million
GEO: Gene Expression Omnibus
GSEA: Gene Set Enrichment Analysis
GTEx: Genotype Tissue Expression
GPSA: genetic prognostic signature based on entire genes
IC50: 50%-inhibition concentration
IPI: Integrated prognostic index by combining the MPS with clinical factors
KEGG: Kyoto Encyclopedia of Genes and Genomes
k-NN: K-nearest neighbor
kPMGs: key PMGs constructing the MPS
LASSO: least absolute shrinkage and selection operator
MF: molecular function
MGs: metabolic reprogramming-associated genes
MPS: metabolic reprogramming-associated prognostic signature
NB: Neuroblastoma
NRI: net reclassification improvement
PMGs: prognostic metabolic reprogramming-associated genes
ssGSEA: single‐sample Gene Sets Enrichment Analysis
TARGET: Therapeutically Applicable Research To Generate Effective Treatments
TF: transcription factor
TROC: time-dependent receiver operating characteristic
