# Supplementary material for: Developing targeted therapies for neuroblastoma by dissecting the effects of metabolic reprogramming on tumor microenvironments and progression
Source: Theranostics. 2024 May 27;14(9):3439–69. doi: 10.7150/thno.93962 (PMC11209723; doi:10.7150/thno.93962)
Supplement: Supplementary file 1 — Supplementary methods, figures and tables. [file thnov14p3439s1.pdf]

Supplementary Materials

Method S1: Identification of prognostic metabolic reprogramming-associated genes (PMGs).

Method S2: Construction of the MPS based on PMGs.

Method S3: Exploration of identified MGs describing NBL metabolism.

Method S4: Elucidation of cellular components of specific immune microenvironment of NBL with MPS-I and MPS-II.

Method S5: Exploration of inflammatory microenvironment of MPS-I.

Method S6: Exploration of potential therapeutic molecular targets for MPS-I and MPS-II NBL.

Method S7 Cell viability measurement

Method S8 Cell proliferation assay

Method S9 Cell migration measurement

Method S10 Cell invasion measurement

Method S11 Cell colony formation measurement

Method S12 Flow cytometry to assess apoptosis

Method S13 TdT mediated dUTP Nick End Labeling (TUNEL) assays

Method S14 Mitochondrial membrane potential measurement

Method S15 Western blotting

Method S16 MPS-I and MPS-II xenograft NBL models

Method S17 Immunofluorescence and immunohistochemistry

Method S18 MPS-I and MPS-II NBL pulmonary metastasis models

Method S19 Pre-processing, clustering, annotation, and differential expressed gene identification of single-cell RNA-seq (scRNA) data

Method S20 Measurement of the cell cycle, copy number variations, stemness of each cell types

Method S21 Enrichment analyses, Gene Set Enrichment Analyses (GSEA), and AUCell

Method S22 Differentiation potential and developmental lineages tracing

Method S23 Cell-cell communication deduction

Method S24 Metabolite flux balance analyses at single-cell level

Method S25 Energy Metabolite flux analyses based on LC/MS-MS

Method S26 Statistical analyses and visualization

Result S1 Proportions of each INSS stages and INRG stratifications within MPS subgroups

Result S2 MPS outperforms a genetic prognostic signature based on all genes

Result S3 Potential molecular targets detection

Result S4 Generation of a nomogram for clinical application

Result S5 Pre-processing, clustering, and annotation of scRNA-seq data

Discussion S1

Figure S1: Validation of MPS in an external test cohort.

Figure S2: For the subgroups of patients that classified by clinical classification approaches, MPS allowed further accurate risk-stratification.

Figure S3: Distribution of INSS stages and INRG stratifications in the MPS-identified NBL subtypes.

Figure S4: Construction and validation of genetic prognostic signature based on all genes.

Figure S5: Comprehensive view of dysregulated pathways between MPS-I and MPS-II NBL

Figure S6: In the test cohort, different expressions of kPMGs in MPS-I and MPS-II NBL patients.

Figure S7: The infiltration atlas of immune cells upon NBL samples in the training and test cohort.

Figure S8: Necrosis levels of cells in control and treated groups.

Figure S9: Nomogram of MPS.

Figure S10 Quality control of scRNA-seq data

Figure S11 Clustering of scRNA-seq data

Figure S12 Annotation and proportion of cell types

Figure S13 CNV inference

Figure S14 Major biological processes of each NBL cell type

Figure S15 Differential metabolites between MPS subtyped cells

Table S1: Clinical parameters and sources for samples.

Table S2: Resultant data of iterative univariable cox regression for metabolic genes.

Table S3: Coefficients of genetic prognostic signature based on all genes.

Table S4: Dysregulated pathways between MPS-I and MPS-II NBL

Table S5: Biological processes, cell components and molecular functions overrepresented by aberrantly expressed MGs.

Table S6: Pathways overrepresented by aberrantly expressed MGs.

Table S7: Biological processes and molecular functions overrepresented by kPMGs.

Table S8: KEGG pathways analyses for kPMGs.

Table S9: Univariable Cox regression for ALDH1A2.

Table.S10: Regulation network of transcription factors and kPMGs.

Table S11: Dysregulated immune cellular components between MPS-I and MPS-II NBL.

Table S12: Dysregulated inflammatory components between MPS-I and MPS-II NBL.

Method S1: Identification of prognostic metabolic reprogramming-associated genes (PMGs)

A list of molecules involved in metabolic reprogramming-associated pathways was obtained from curated gene sets of Kyoto Encyclopedia of Genes and Genomes (KEGG) in the Gene Set Enrichment Analysis (GSEA) database[1]. With this extracted gene list, the expression profiles of metabolic reprogramming-associated genes (MGs) in the control and NBL cohorts were extracted. A differential expression analysis was subsequently conducted to identify aberrantly expressed MGs in patients with NBL using the limma package in R. The chromosome locations of MGs were obtained from annotations supported by the Ensembl database[2].

A univariate Cox proportion hazard regression analysis was performed using the survival package in R to further screen the PMGs in MG expression profiles and evaluate the association between each MG and the overall survival time of each patient in the NBL cohort; MGs with p-values < 0.05 were considered PMGs.

Method S2 Construction of the MPS based on PMGs

In the training cohort, least absolute shrinkage and selection operator (LASSO) was first applied to further narrow the group of candidate PMGs, and thereby the risk of overfitting was accessible to minimization. A multivariable Cox hazard regression analysis was conducted using the stepwise method using Akaike information criterion (AIC) to eliminate the PMGs with multicollinearity[3], thereby the MPS was identified for modeling.

LASSO and Cox regression analyses were performed using the glmnet, rms and survival packages, and the MPS was modeled with the following formula:

$$\textbf{MPS score} = \sum_{i=1}^{n} \beta_i \times E_i$$

where n represents the total number of PMGs included in the **Metabolic prognostic signature**, β_i represents the regression coefficient of gene i, and E_i represents the log transformed expression of gene i.

Method S3 Exploration of identified MGs describing NBL metabolism

MGs associated with metabolic reprogramming in NBL were investigated using Gene Ontology (GO) annotation and KEGG enrichment analysis with the clusterProfiler package in R[4]. A biological understanding of the MPS in risk groups was attained using a Gene Set Enrichment Analysis (GSEA, version 4.0.3), and the reference list of genes was a curated gene set from KEGG that was provided by GSEA[1, 5]. GO and KEGG analyses were also conducted in STRING (string-db.org version 11.0) to obtain an understanding of the biological functions of key PMGs constructing the MPS (kPMGs).

Protein-protein interactions (PPIs) among kPMGs were analyzed using STRING. The transcription factor (TF) and kPMG regulatory networks were identified using the protocol described below. A list of TFs involved in tumor progression was collected from the Cistrome database[6], and the differentially expressed TFs (DETFs) in NBL were extracted by referring to this list and based on a differential expression analysis using limma in R. Thus, Spearman's correlation coefficients were calculated to identify the regulatory relationships between DETFs and kPMGs, and Spearman's correlation coefficients > 0.4 and p-values < 0.01 were considered regulatory relationships.

Method S4 Elucidation of cellular components of specific immune microenvironment of MPS-I and MPS-II NBLs

Estimation of stromal and immune cells in malignant tumor tissues using expression data (ESTIMATE) algorithm is a sophisticated algorithm which designed for measuring the degree of infiltration of cancer cells and different normal cells by exploiting the unique properties of tumor cell transcriptional profiles[7], its robustness is validated in various cancers including NBL[7, 8]. The present study employed ESTIMATE algorithm which provided by ESTIMATE package in R, to quantify the global tumor-microenvironment into four characterized indictors, including Stromal score, Immune score, ESTIMATE score, and Tumor purity. Where, those indictors represent infiltration abundance of stromal cells, immune cells, overall normal cells, and tumor cells, respectively. Since the resultant data met skew distribution, grouped comparison was performed with Wilcoxon test, and Spearman coefficients evaluated their correlation. All p-values were corrected using Benjamini–Hochberg method to avoid false positive results.

To further dissect the detailed atlas of various immune cells' infiltration, single-sample Gene Sets Enrichment Analysis (ssGSEA) was conducted for independently measuring 28 types of immune cellular components that infiltrated in each NBL clinical samples, and outline the comprehensive view of immune cells' infiltration of MPS-I and MPS-II NBL. This typical method was elaborated in our previous work[8]. Briefly, ssGSEA was performed for transcriptome data of each NBL sample by GSVA and GSEABase packages in R, the referred gene sets used in this program was published gene sets of 28 types of immune cellular components[9]. For exhibition of atlas of identified dysregulation of immune cellular components, of which the enrichment scores were scaled using pheatmap packages. Furthermore, the kernel density estimation was performed for each NBL sample by ggridges package, to describe the density distribution of each immune cellular components in the MPS-I and MPS-II NBL. The interactive network of these components was delineated by ggcor package with Spearman coefficient, which also employed for assessing the correlation between these and MPS.

Method S5 Exploration of inflammatory microenvironment of MPS-identified MPS-I NBL

GO gene sets describing biological process (BP), cellular component (CC) and molecular function (MF) were obtained from Gene Set Enrichment Analysis (GSEA) dataset, and then 45 gene sets associated with inflammation were extracted for independently estimating the inflammatory microenvironment of each NBL sample. For exhibition of inflammatory microenvironment atlas, pheatmap package was also employed to scale the enrichment score of corresponding inflammatory components of each sample. Correlation analyses were conducted using Spearman coefficient, and further interactive network analyses were performed with Cytoscape software.

Method S6 Exploration of potential therapeutic molecular targets for MPS-I and MPS-II NBL

Molecules with promising potential for targeted therapies, including immune microenvironment-associated molecules and inflammation-associated molecules[8, 10], were included for potential therapeutic targets' detection. As pervious reported, molecules with

significantly different expression level between MPS-I and MPS-II NBL were deemed the promising therapeutic targets.

Method S7 Cell viability measurement

Human NBL cell lines of SKNSH and SKNAS were purchased from Procell Life Science&Technology Co.,Ltd. (Wuhan, China). SKNSH cells were grown in Minimum Essential Medium (Gibco®, USA) supplemented with 10% FBS (Gibco®, USA), 1% penicillin–streptomycin (Beyotime Company, China). SKNAS cells were grown in Dulbecco's Modified Eagle Medium (Gibco®, USA) supplemented with 10% FBS (Gibco®, USA), 1% penicillin–streptomycin (Beyotime Company, China). All cells viability assays were performed according to the CCK-8 Kit (Beyotime Company, China). Compounds were tested at appropriate serial concentrations, with each concentration duplicated five times. Cells were exposed to treatment for 48 h, and the absorbance was measured at 450 nm with a microplate reader (ELX808, BioTek, German). The IC50 values were calculated using GraphPad Prism version 5.0. For the following other assays, cells were treated with etoposide an AZD7762 according to their corresponding IC50.

Method S8 Cell proliferation assay

The 5-ethynyl-20-deoxyuridine (EdU) incorporation assay was designed to accurately quantify the DNA duplication that could direct quantify the cell proliferation ratio. The inhibition of cell proliferation impacted by etoposide an AZD7762 was determined by EdU assay according to our previous publication[11]. Cells were exposed to treatment for 48h, and were stained for analyses in digital microscope system (IX81, Olympus). Each experiment was carried out in triplicate.

Method S9 Cell migration measurement

According to the previous research, the inhibition effect of etoposide an AZD7762 on NBL cells' migration ability were quantified using cell wound healing assay[12]. After cell wounds created in the 6-wells plates, all cells were exposed to treatment for 48h, and digital microscope system (IX81, Olympus) and Image-Pro Plus 6.0 were used to analyzed the cell wound healing ratio at 0, 24, and 48h. Each experiment was carried out in triplicate.

Method S10 Cell invasion measurement

Transwell assay was employed to estimate the inhibition effects of etoposide an AZD7762 on NBL cells invasion. The 24-transwell plates were covered with Matrigel matrix at a 1:8 dilution ratio. 2 × 105 cells in 200 μL of corresponding medium without FBS were placed in the upper chamber, with 800 μL corresponding medium containing 20% FBS being placed in the lower chamber. After incubation for 48 h at 37°C with 5% CO2, the cells that had invaded through the bottom chambers were fixed with 4% paraformaldehyde for 20 min and then stained with 0.05% crystal violet for 30 min. The amount of invaded cells was calculated by observation under a microscope. Each experiment was carried out in triplicate.

Method S11 Cell colony formation measurement

Cells were seeded into 6-well plates at 500 cells per well, separately, and were exposed to

treatment. Two weeks later, the cells were fixed in 75% alcohol for 30 min and stained with 0.05% crystal violet for 30 min. The colonies imaged under a microscope and calculated using the Image-Pro Plus 6.0. The experiments were performed in triplicate to calculate the mean.

Method S12 Flow cytometry to assess apoptosis

The rate of apoptosis was assessed by flow cytometry. Briefly, treated cells were collected by centrifugation at 300 g for 5 min and rinsed twice with pre-chilled PBS. Next, collected cells were carefully resuspended in 100 μl of binding buffer at concentration of 1×106 cells/ml. 10 μl 7-Aminoactinomycin D (7-AAD) and 5 μl phycoerythrin (PE)-labeled Annexin V (Yeason, Shanghai, China) were added, followed by staining at room temperature for 20 min in the dark, and then raised cell suspension to 500 μl with binding buffer for estimating apoptotic rate by flow cytometry (Becton-Dickinson and Company). The experiments were performed in triplicate to calculate the mean.

Method S13 TdT mediated dUTP Nick End Labeling (TUNEL) assays

TUNEL Apoptosis Detection Kit (Yeason, Shanghai, China) was used to detect the breakage of nuclear DNA in cells and tissues during the late stages of apoptosis. For cells slices, the treated cells were fixed with 4% paraformaldehyde for 20 min and rinsed twice with PBS. The fixed cells were incubated with 0.2% Triton X-100 for 5 min for permeabilization, and rinsed twice with PBS. Then, according to the instructions provided by the manufacturer, cells were stained using FITC-12-dUTP Labling Mix and Recombinant TdT Enzyme, and cells nuclei were stained using DAPI. For paraffin-embedded tissue sections, sections were treated with xylene and gradient ethanol, followed by permeabilization with 20 μg/mL Proteinase K solution. Subsequent TUNEL experimental steps were similar to those for cell slices. All stained sections were analyzed in digital microscope system (IX81, Olympus). Each experiment was carried out in triplicate.

Method S14 Mitochondrial membrane potential measurement

Enhanced mitochondrial membrane potential assay kit with JC-1 (Beyotime, Shanghai, China) was employed for measuring the mitochondrial membrane potential. Briefly, treated cells were incubated with JC-1 solution for 20min at 37℃, and rinsed twice using staining buffer for analyses in digital microscope system (IX81, Olympus). Then, DAPI/PI staining was employed to avoid the necrosis-induced false positive results of JC-1 assays. Each experiment was carried out in triplicate.

Method S15 Western blotting

Western blotting was used to quantify the proteins expression of treated cells, and the protocol was according to our previous publications [12]. Primary antibodies: cleaved caspase-3 (cat. no. 9661; Cell Signaling Technology, US); cleaved caspase-9 (cat. no. 20750; Cell Signaling Technology, US); bax (cat. no. 5023; Cell Signaling Technology, US); bcl-2 (cat. no. 4223; Cell Signaling Technology, US); CD209 (cat. no. PHX6604; Abmart, China); β-tubulin (cat. no. PA4302; Abmart, China); GAPDH (cat. no. MA9166; Abmart, China). Secondary antibody: horseradish peroxidase-conjugated goat anti-rabbit (cat. no. GB23303; Servicebio, Inc.); horseradish peroxidase-conjugated goat anti-mouse (cat. no. GB23301; Servicebio, Inc.). A

chemiluminescence luminol reagent (cat. no. G2014, Servicebio, China) was used to visualize the protein bands employing the Image Lab 5.2 quantitative assay system (Bio-Rad Laboratories, US). The relative protein levels were determined by normalizing to β-tubulin or GAPDH.

Method S16 MPS-identified MPS-I and MPS-II xenograft NBL models

The Ethics Committee of Renmin Hospital of Wuhan University approved the study protocol and all the animal research procedures were performed according to the institutional ethical standards and/or those of the national research committee and according to the 1964 Helsinki Declaration and its later amendments or comparable ethical standards. The collected 1.5×10^7 MPS-identified MPS-I cells (SKNAS) and MPS-II cells (SKNSH) were washed in serum-free medium, suspended in 100 μL of PBS, and implanted subcutaneously into the dorsal area of male BALB/c nude mice (5 weeks old), purchased from Beijing Life River Experimental Animal Technology Co. Ltd. (Beijing, China).

When the tumor volume was approximately 150 mm^3, MPS-I and MPS-II nude mice were respectively randomly divided into three groups (n = 8 per group), which were treated via caudal vein injection with normal saline, AZD7762 (5.2μM/g), and etoposide(14.6μM/g). The tumor volume was measured every 3 days. The tumor volume (TV) was calculated using the following formula: TV (mm3) = d2 × D/2, where d and D represent the shortest and longest diameters, respectively. After 36 days, the mice were euthanized their tumors were isolated, and hematoxylin-eosin (H&E) staining were employed for histological assessment

Method S17 Immunofluorescence and immunohistochemistry

Immunofluorescence and immunohistochemistry were performed according to our previous publication with some modifications [12]. Ki67 immunofluorescence was employed for proliferation capacity measurement, and immunohistochemistry of cleaved caspase-3, cleaved caspase-9, bax, and bcl-2 were used to evaluate the activity of mitochondrial pathway. Primary antibodies: ki67 (cat. no. 9129; Cell Signaling Technology, US); cleaved caspase-3 (cat. no. 9661; Cell Signaling Technology, US); cleaved caspase-9 (cat. no. 20750; Cell Signaling Technology, US); bax (cat. no. 5023; Cell Signaling Technology, US); bcl-2 (cat. no. 4223; Cell Signaling Technology, US); CD209 (cat. no. PHX6604; Abmart, China). Secondary antibodies: Alexa Fluor 594 -conjugated goat anti-rabbit (cat. no. 8889; Cell Signaling Technology, US); Alexa Fluor 594 -conjugated goat anti-mouse (cat. no. 8890; Cell Signaling Technology, US); biotinylated goat anti-rabbit secondary antibody (cat. no. ab205718; Abcam). Image Pro Plus 6.0 analysis software (Media Cybernetics, Inc.) was used to analyze the results.

Method S18 MPS-I and MPS-II NBL pulmonary metastasis models

The male BALB/c nude mice (5 weeks old) were respectively randomly divided into six groups (n = 8), in which three groups were used to establish MPS-I NBL lung metastasis models, and other groups were used to construct MPS-II models. The collected MPS-I cells (SKNAS) and MPS-II cells (SKNSH) were washed in serum-free medium, suspended in 120 μL of PBS, and cells were injected via caudal vein into mice. One week after tail vein injection, the mice started to receive treatment of normal saline, AZD7762, and etoposide according their risk groups, respectively. Survived mice were sacrificed after 8 weeks of treatment to evaluate

pulmonary metastasis, and hematoxylin-eosin (H&E) staining were employed for histological assessment. The overall survival time analyses were preformed using K-M curve and log-rank test.

Method S19 Pre-processing, clustering, annotation, and differential expressed gene identification of single-cell RNA-seq (scRNA) data

The scRNA-seq data and clinical characteristics of 16 NBL patients was obtained from GEO (GSE137804). Seurat (version 4.3.0.1) was employed to pre-process scRNA-seq data. The doublets were detected and removed using DoubletFinder (version 2.0.3). Afterwards, low-quality cells were filtered out if 1) RNA counts < 500; 2) recognizable genes < 300 or > 6000; 3) mitochondrial genes > 20%. Gene expression was normalized, and the batch effect among different samples was corrected by Harmony (version 1.0). The top 2000 highly variable genes were distinguished for Principal Component (PC) Analysis (PCA), and the identified PCs were used to cluster cells into different populations. All clusters were annotated as cells with biological identity by well-known biomarkers. Finally, the differential expressed genes and the biomarker genes of each cell type were identified, they should meet: 1) |log2 fold change| > 0.25; 2) adjusted p-value < 0.05.

Method S20 Measurement of the cell cycle, copy number variations, stemness of each cell types

The cell cycle was inferred using built-in functions of Seurat. InferCNV (version 1.12.0) were utilized to calculate the CNV level. The copy-number karyotyping of aneuploid tumors were generated using copy-kat, to distinguish non-malignant and malignant cells. In the present study, the endothelial cells were set as reference to identify NBL cells with significant chromosomal copy number variation. AUCell algorithm was conducted to estimate stemness of each single cell with stemness gene set, which included ABCG2, BMI1, CD34, CD44, CTNNB1. EPAS1, EZH2, HIF1A, KDM5B, KLF4, LGR5, MYC, NANOG, NES, NOTCH1, POU5F1, SOX2, TWIST1, ZFP42, ZSCAN4.

Method S21 Enrichment analyses, Gene Set Enrichment Analyses (GSEA), and AUCell

The enrichment analyses were conducted with GO BP terms as reference. The positively expressed genes within each cell type or subtype were sent into enrichment analyses to detect its major biological functions. GSEA was a ranking-based method to directly quantify the degree of up- or down-regulation of all GO BP terms. With all RNA expression profiling as input, it offered more detailed information other than enrichment analyses. The AUCell algorithm allowed us to quantify the activities of given gene set/pathways/biological processes within each single cells. Its high-resolution nature allowed us to gain more detailed biological insights. The enrichment and GSEA analyses were conducted using ClusterProfiler (version 4.6.0), and AUCell was conducted with AUCell (version 1.24.0).

Method S22 Differentiation potential and developmental lineages tracing

With CytoTRACE (version 0.3.3), the relative differentiation state of each single cell was recovered from RNA expression profiling without any additional data or knowledge. Briefly, a KNN graph with undirected information among cells was deduced to determine the time order of cells. Afterwards, a transfer matrix was generated to predict differentiation potential

of cells.

Slingshot (version 2.6.0) was employed to trace the development lineages of NBL cells. Briefly, Slingshot was a two-steps method, included 1) the inference of the global lineage structure and 2) the inference of pseudotime variables for cells along each lineage. A minimum spanning tree was generated from identified clusters to detect key elements of the global lineage structure, i.e., the number of lineages and where they branch. Afterward, simultaneous principal curves were utilized to fit smooth branching curves to these lineages, and then translated the knowledge of global lineage structure into stable estimates of the underlying cell-level pseudotime variable for each lineage.

Method S23 Cell-cell communication deduction

CellChat package (version 1.4.0), which integrated network analyses, patterns identification, and many learning methods, was employed to detect and quantify the communication among cells. For determine the sender, receiver, mediator, and influencer of each signaling, CellChat utilized various metrics of weighted directed graph, including outgoing and incoming degree, etc.

Method S24 Metabolite flux balance analyses at single-cell level

The scFEA (version 1.1.2) was employed to recover metabolite flux from scRNA-seq data. The scFEA utilizes a meticulously restructured human metabolic map with targeted metabolic modules, alongside an innovative probabilistic model that applies flux balance constraints to scRNA-seq data. Additionally, it incorporates a cutting-edge graph neural network optimization solver. This system effectively captures the complex progression from transcriptome to metabolome by employing multi-layer neural networks, which adeptly handle the non-linear relationship between enzymatic gene expressions and reaction rates.

The metabolic signaling analyses were conducted using MetaboAnalyst (version 6.0), which employed Small Molecule Pathway Database (SMPDB) as its reference. Furthermore, GSEA was conducted to quantify the dysregulation level of metabolic signalings.

Method S25 Energy Metabolite flux analyses based on LC/MS-MS

HPLC grade acetonitrile (ACN) and methanol (MeOH) were purchased from Merck (Darmstadt, Germany). MilliQ water (Millipore, Bradford, USA) was used in all experiments. All standards were purchased from Sigma-Aldrich (St. Louis, MO, USA) and Zhenzhun, etc. Formic acid was purchased from Sigma-Aldrich (St. Louis, MO, USA). Stock solutions of standards were prepared at 1 mg/mL in MeOH and other solutions. All stock solutions were stored at -20°C. Stock solutions were diluted with MeOH to working solutions prior to analysis. The sample was thawed on ice, 100 μL ultrapure water extract was added to resuspend the cell pellet. The 50 μL cell suspension was divided and 200 μL methanol (precooled at -20°C) was added and vortexed for 2 minutes at 2500 rpm. The sample was frozen in liquid nitrogen for 5 min, removed on ice for 5 min, then the sample was vortexed for 2 min and the previous step was repeated 3 times. The sample was centrifuged at 12000 rpm for 10 min at 4°C. Transfer 200 μL of the supernatant into a new centrifuge tube and place the supernatant in a -20°C refrigerator for 30 minutes, then centrifuge the supernatant at 12000 rpm for 10 minutes at 4°C.

After centrifugation, 180 μL of the supernatant was transferred through a protein precipitation plate for further LC-MS analysis. The left 50 μL cell suspension was frozen and thawed three times, centrifuged at 12,000 rpm for 10 minutes, and the supernatant was collected to determine the protein concentration using the BCA Protein Assay Kit.

The sample extracts were analyzed by LC-ESI-MS/MS system (Waters ACQUITY H-ClassD, https://www.waters.com/nextgen/cn/zh.html; MS, QTRAP® 6500+ System, https://sciex.com /). The analytical conditions were as follows

Amide method: HPLC: column, ACQUITY UPLC BEH Amide (i.d.2 .1×100 mm, 1.7 μm); solvent system, water with 10 mM ammonium acetate and 0.3% ammonium hydroxide (A), 90% acetonitrile/water (V/V) (B); the gradient was started at 95% B (0-1.2 min), decreased to 70% B (8 min), 50% B (9-11 min), finally ramped back to 95% B (11.1-15 min); flow rate, 0.4 mL/min; temperature, 40°C; injection volume, 2 μL.

Linear ion trap (LIT) and triple quadrupole (QQQ) scans were acquired on a triple quadrupole linear ion trap (QTRAP) mass spectrometer, QTRAP® 6500+ LC-MS/MS system, equipped with an ESI Turbo Ion Spray interface, operating in both positive and negative ion modes and controlled by Analyst 1.6.3 software (Sciex). The operating parameters of the ESI source were as follows: ion source, ESI+/-; source temperature 550 ℃; ion spray voltage (IS) 5500 V （positive）,-4500 V(negative); curtain gas (CUR) was set at 35 psi, respectively. Energy and its metabolites were analyzed using scheduled multiple reaction monitoring (MRM). Data acquisition was performed using Analyst 1.6.3 software (Sciex). All metabolites were quantified using Multiquant 3.0.3 software (Sciex). Mass spectrometer parameters, including declustering potentials (DP) and collision energies (CE) for individual MRM transitions, were performed with further DP and CE optimization. A specific set of MRM transitions was monitored for each time period according to the metabolites eluted within that time period.

Method S26 Statistical analyses and visualization

For bioinformatical research part. The raw data were collated by Practical Extraction and Report Language (PERL, version 5.30.0) and R software (version 3.61). The statistical analysis and all software packages were applied using R software (version 3.61); the visualization of the statistical results and interactive network data analysis were performed using Cytoscape (version 3.7.1). A detailed description of the statistical analyses is provided below. The differential expression analysis used the Wilcoxon test, and the genes with a $|\log_2 foldchange| > 1$ and an FDR < 0.05 were considered aberrantly expressed MGs, in which the FDR represents the p-value corrected using the false discovery rate method. The correlation analysis conducted according to the types of variables, and Pearson's correlation coefficients were calculated for continuous variables and Spearman's correlation coefficients were calculated for discrete variables. Comparisons of gene expression, and IC50 of candidate drugs between different groups were performed using the Wilcoxon test. For the PPI analysis, we comprehensively considered the degree and combined score to identify a core gene.

For biological validation part. The statistical analysis and all software packages were applied using R software (version 3.61), and the visualization of the statistical results were performed using OriginPro (version 9.8). Grouped comparison (n > 2) were preformed using Fisher's or Welch's one-way ANOVA test (a pairwise comparison using Games-Howell test).

Results S1 Proportions of each INSS stages and INRG stratifications within MPS subgroups

The proportions of each INSS stages and INRG stratifications within MPS-identified different NBL subtypes were investigated. As indicated in Fig. S3, there are much more patients with INSS stage 4 and INRG M in the MPS-identified MPS-I group than those in the MPS-II group. The proportion of each INSS stage and INRG stratification in NBL patients was almost similar in MPS-II group. Specifically, INSS stage 1 and INRG L1, L2 patients were slightly more than other stages and INRGs in the MPS-II group, these findings also validated in the test cohort. The difference of distribution of stages and INRGs between MPS-identified MPS-I and MPS-II groups reached statistical significance, and such findings indicated that the results draw from the analysis of INRGs and stages might incorrectly grouped those patients with favorable prognosis to the high-risk stages and INRGs, Unfortunately, such hazardous classification might most likely makes catastrophic effect on the individualized treatment to these patients.

Results S2 MPS outperforms a genetic prognostic signature based on all genes

We tested whether MPS outperforms a genetic prognostic signature based on all genes. Differential genes analyses identified 2332 upregulated genes and 1559 down-regulated genes (Fig.S4 A), in which a total of 1144 genes were confirmed as prognostic genes by iterative univariable Cox regression. Then, LASSO regression maintained 40 genes for model training (Fig.S4 B and C), and finally multi-variable Cox regression included 25 genes to build genetic prognostic signature based on entire genes (GPSA, Table.S3). However, as shown with TROC results (Fig.S4 D), the AUC value of GPSA was less than that of the present MPS in the test cohort (0.836 vs. 0.860), which indicated that the MPS still presented superior accuracy than GPSA for prognostic estimation. The reason might be that the gene capacity of GPSA was highly greater than that of MPS (25 vs. 12), such greater number of genes used rendered GPSA more noise and made GPSA more likely overfit. Such trend was also evidenced by higher AUC value of GPSA achieved in the training cohort. Alternatively, overfitting in the training cohort compromised the robustness and generalization of GPSA, which finally limited the performance of GPSA applied in the independent test cohort. In conclusion, MPS showed better suitability to the potential clinical application, as compared with GPSA.

Result S3 Potential molecular targets detection

Considering that immunotherapy against immune checkpoint has achieved certain clinical progress, we examined the profile differences of key immune checkpoint genes expressed in MPS-I and MPS-II NBL samples, and found that only LAG3 manifested differential expression between MPS-stratified subgroups (Fig.6 D). High expression of LAG3 in MPS-II NBL indicated that clinical treatment of MPS-II patients could benefit from the immunotherapy targeting LAG3, which could potentially update the current clinical immunotherapy for NBL.

Subsequently, we further examined the expression of inflammatory-immune related molecules that have been reported to be associated with NBL progression in order to explore the potential therapeutic molecular targets [13]. As demonstrated in Fig.7 D, total 13 key inflammatory-immune related molecules were differentially expressed between MPS-I and MPS-II NBL, which include chemokine related molecules, interleukin family, T cell marker CD4, etc. Notably, high expression of NFKB1 oncogene was found in MPS-identified MPS-I NBL, which strongly indicated that the NFKB1-targeted drugs would greatly facilitate the clinical treatment of MPS-I NBL since a NF-κB pathway mainly regulated by NFKB1 has been widely confirmed to play an important role in tumor progression.

Result S4 Generation of a nomogram for clinical application

The MPS was visualized as a nomogram to facilitate its clinical application, and thereby a quantitative approach was generated for clinicians (Fig.S9). The usage of nomogram is described below. The calculated value of the MPS to the "MPS" axis in the nomogram is identified, and then a straight line is drawn vertically downward from the corresponding point on the axis to determine the 1-, 3- and 5-year survival rates of patients with NBL.

Result S5 Pre-processing, clustering, and annotation of scRNA-seq data

Initially, quality control measures were executed on the scRNA-seq data derived from 16 NBL patients. This process involved the exclusion of duplicates and cells of subpar quality, as evidenced in Figure S10. Subsequently, the most variable 2000 genes were discerned (Figure

S11A), facilitating the application of the Harmony algorithm. This algorithm was instrumental in integrating scRNA-seq data from disparate samples and correcting batch effects, as depicted in Figure S11B. Following this, a Principal Component Analysis (PCA) was conducted to isolate the foremost 15 principal components, which were then used to cluster all cells at a resolution of 1.2 (Figures S11C and D). In the final stage, a variety of biomarkers were employed to ascertain the identities of the distinct cell clusters, as shown in Figure S12A.

Discussion S1

Lymphocyte activation gene-3 (LAG3), emerging as a third IR used in the current targeted immune therapy, has achieved promising efficacy in a couple of solid tumors such as non-small cell lung cancer[14]. Recently, LAG3 administration turns to be helpful in offsetting the defects occurred in the CTLA4 and PD1-PDL1-targeted immunotherapy of cancer, thus gains considerable interest and current scrutiny[14]. NFKB1, as one of five subunits of NF -κB, has been widely identified to play an important role in tumorigenesis[15]. For example, the increased expression of NFKB1 contributes to the progress of breast, colorectal and pancreatic cancer[15]. Interestingly, LAG3 and NFKB1 have been revealed in our study to serve as key components in the immune-inflammatory microenvironment of NBL. Briefly, significant up-regulation of LAG3 expression was shown in MPS-identified MPS-II NBL, whereas remarkable increasing expression of NFKB1 exhibited in MPS-identified MPS-I NBL. In view of such significant expression trends in the subtypes of NBL identified with MPS, LAG3 and NFKB1-targeted molecular immunotherapeutic strategy might be highly promising and would greatly promote the clinical treatments of NBL in the near future.

Supplementary references

1. Subramanian A, Tamayo P, Mootha VK, et al. Gene set enrichment analysis: a knowledge-based approach for interpreting genome-wide expression profiles. Proc Natl Acad Sci U S A 2005; 102: 15545-15550. 2005/10/04. DOI: 10.1073/pnas.0506580102.
2. Cunningham F, Achuthan P, Akanni W, et al. Ensembl 2019. Nucleic Acids Res 2019; 47: D745-D751. 2018/11/09. DOI: 10.1093/nar/gky1113.
3. Akaike HJItoac. A new look at the statistical model identification. 1974; 19: 716-723.
4. Yu G, Wang LG, Han Y, et al. clusterProfiler: an R package for comparing biological themes among gene clusters. OMICS 2012; 16: 284-287. 2012/03/30. DOI: 10.1089/omi.2011.0118.
5. Mootha VK, Lindgren CM, Eriksson KF, et al. PGC-1alpha-responsive genes involved in oxidative phosphorylation are coordinately downregulated in human diabetes. Nat Genet 2003; 34: 267-273. 2003/06/17. DOI: 10.1038/ng1180.
6. Liu T, Ortiz JA, Taing L, et al. Cistrome: an integrative platform for transcriptional regulation studies. Genome Biol 2011; 12: R83. 2011/08/24. DOI: 10.1186/gb-2011-12-8-r83.
7. Yoshihara K, Shahmoradgoli M, Martinez E, et al. Inferring tumour purity and stromal and immune cell admixture from expression data. Nat Commun 2013; 4: 2612. 2013/10/12. DOI: 10.1038/ncomms3612.

8. Jin W, Zhang Y, Liu Z, et al. Exploration of the molecular characteristics of the tumor-immune interaction and the development of an individualized immune prognostic signature for neuroblastoma. J Cell Physiol 2021; 236: 294-308. 2020/06/09. DOI: 10.1002/jcp.29842.

9. Charoentong P, Finotello F, Angelova M, et al. Pan-cancer Immunogenomic Analyses Reveal Genotype-Immunophenotype Relationships and Predictors of Response to Checkpoint Blockade. Cell Rep 2017; 18: 248-262. 2017/01/05. DOI: 10.1016/j.celrep.2016.12.019.

10. Hua X, Chen J, Su Y, et al. Identification of an immune-related risk signature for predicting prognosis in clear cell renal cell carcinoma. Aging (Albany NY) 2020; 12: 2302-2332. 2020/02/07. DOI: 10.18632/aging.102746.

11. Deng P, Jin W, Liu Z, Gao M, Zhou J: Novel multifunctional adenine-modified chitosan dressings for promoting wound healing. Carbohydr Polym 2021, 260:117767.

12. Zhang Y, Cai W, Han G, Zhou S, Li J, Chen M, Li H: Panax notoginseng saponins prevent senescence and inhibit apoptosis by regulating the PI3KAKTmTOR pathway in osteoarthritic chondrocytes. Int J Mol Med 2020, 45(4):1225-1236.

13. Jin W, Zhang Y, Liu Z, Che Z, Gao M, Peng H. Exploration of the molecular characteristics of the tumor-immune interaction and the development of an individualized immune prognostic signature for neuroblastoma. J Cell Physiol. 2021; 236: 294-308.

14. Andrews LP, Marciscano AE, Drake CG, Vignali DA. LAG3 (CD223) as a cancer immunotherapy target. Immunol Rev. 2017; 276: 80-96.

15. Concetti J, Wilson CL. NFKB1 and Cancer: Friend or Foe? Cells. 2018; 7.

Figure S1 Validation of MPS in an external test cohort.

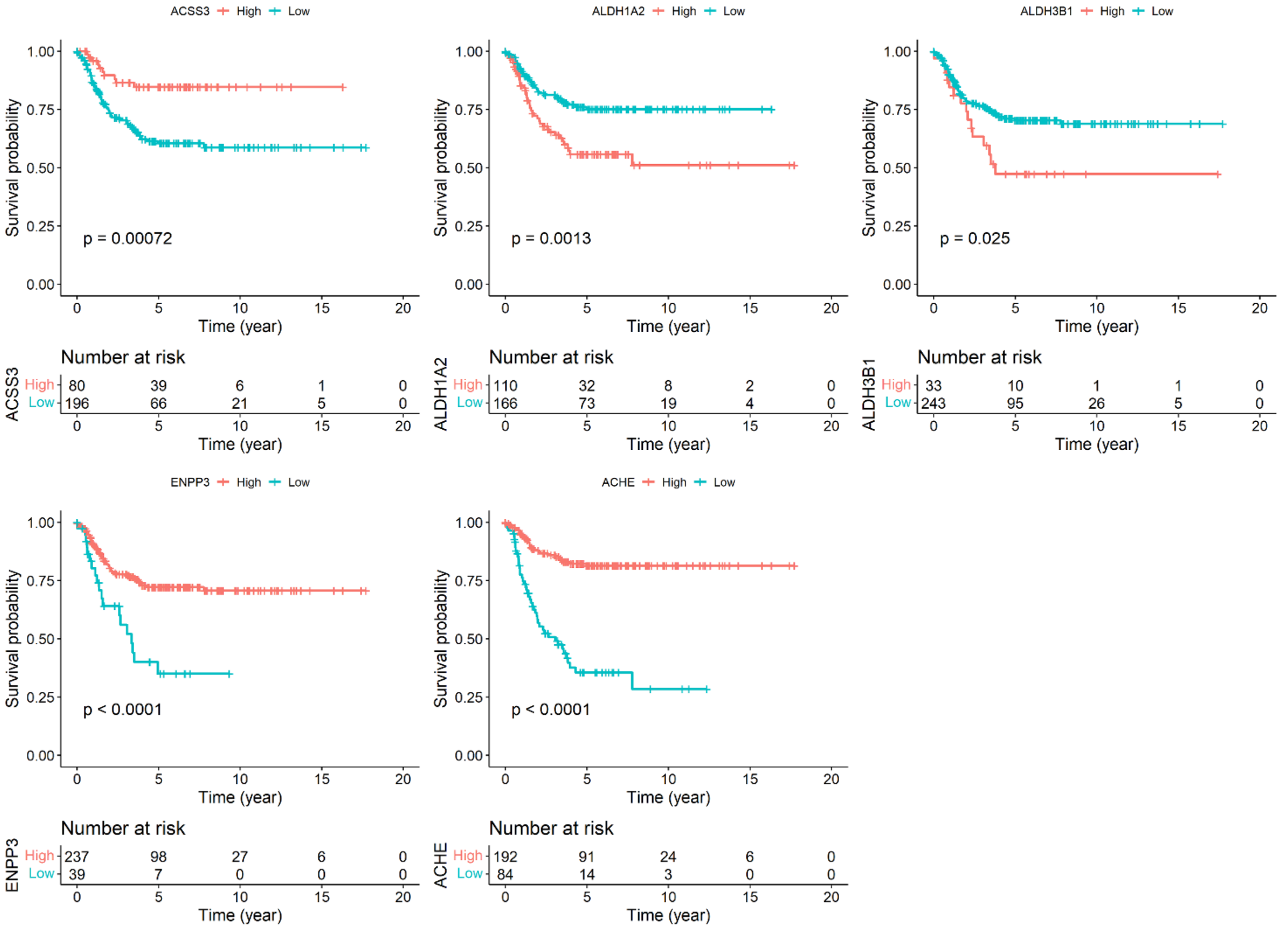

Figure S2 For the subgroups of patients that classified by clinical classification approaches, MPS allowed further accurate risk-stratification.

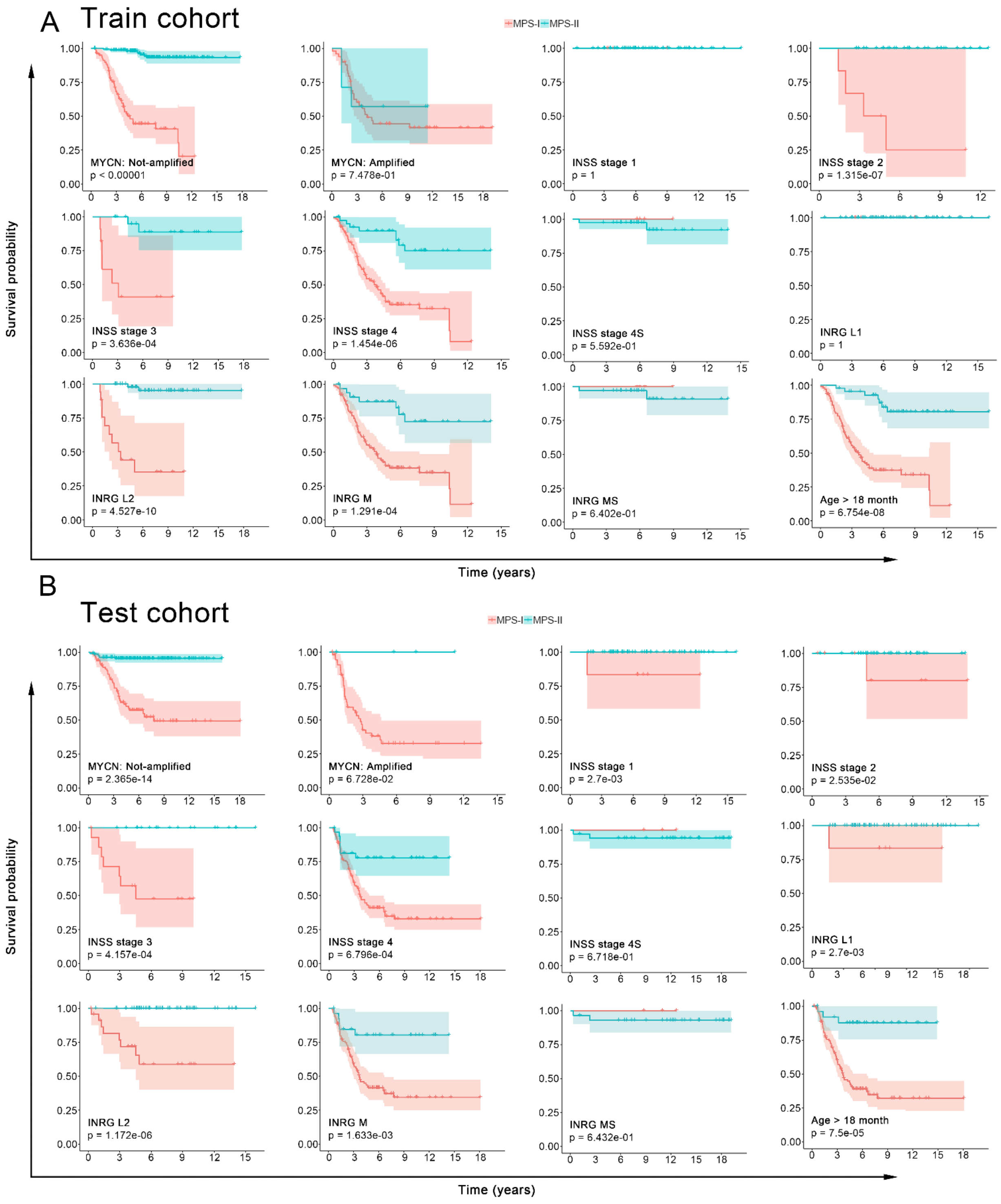

Figure S2 For the subgroups of patients that classified by clinical classification approaches, MPS allowed further accurate risk-stratification.

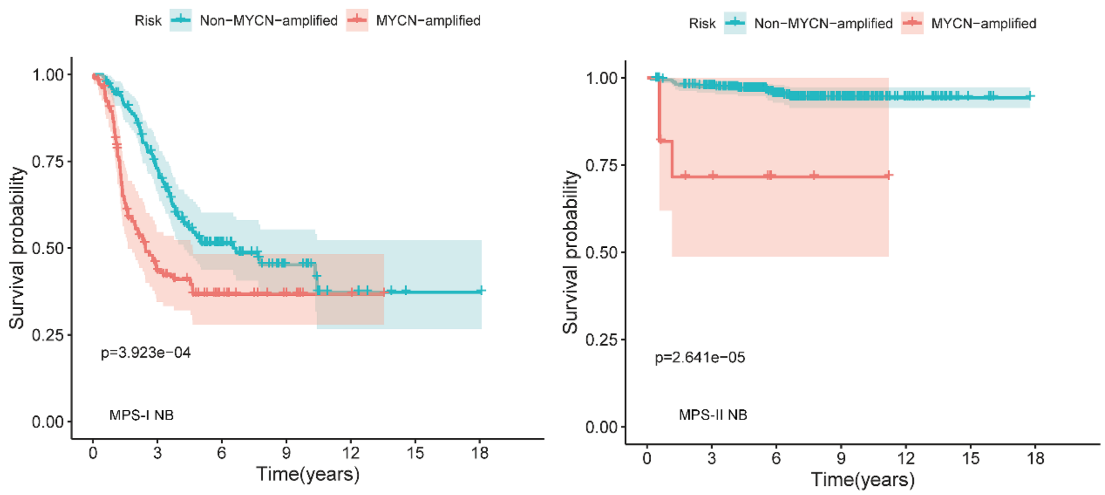

Figure S3 Distribution of INSS stages and INRG stratifications in the MPS-identified NBL subtypes.

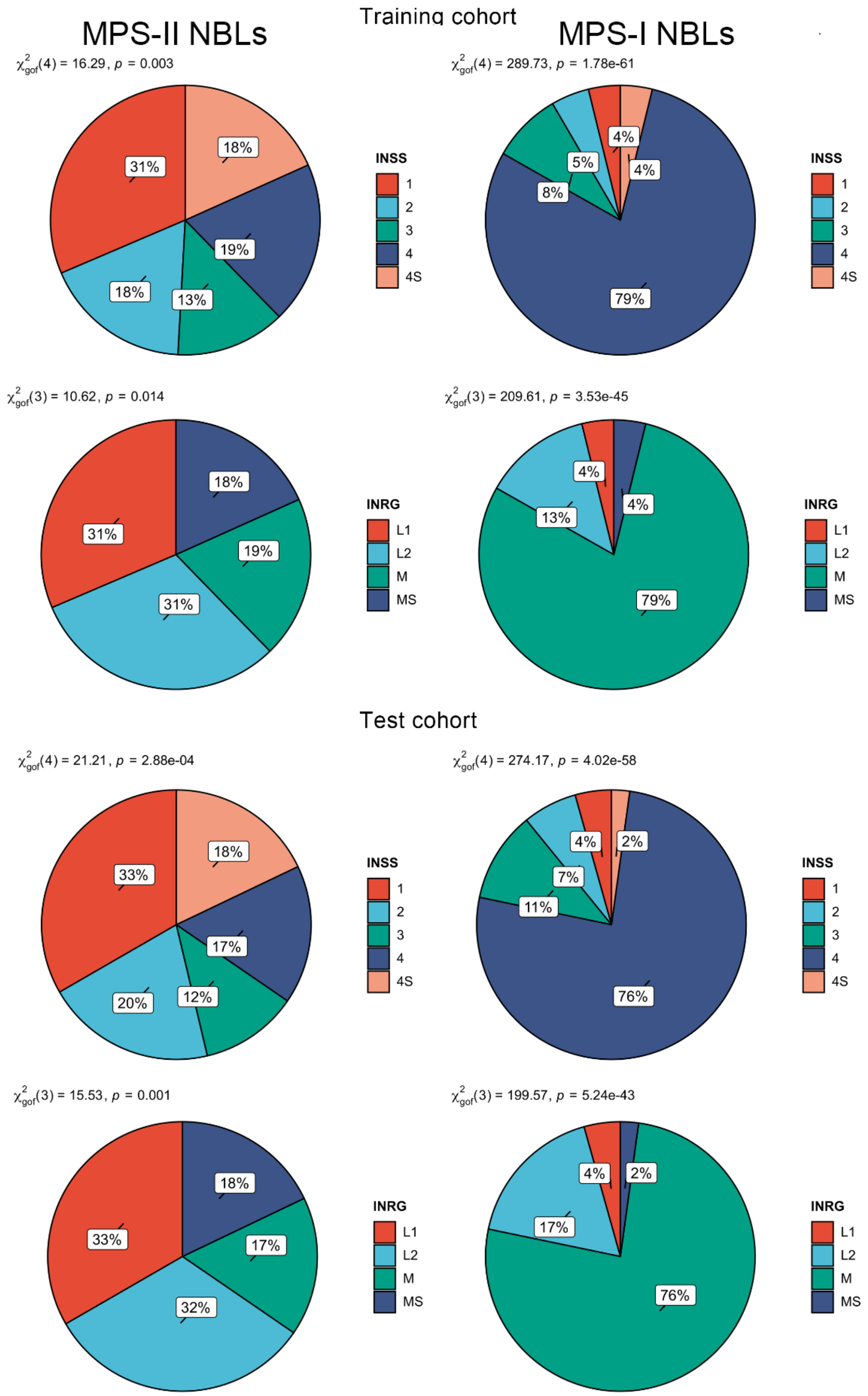

Figure S4 Construction and validation of genetic prognostic signature based on all genes.

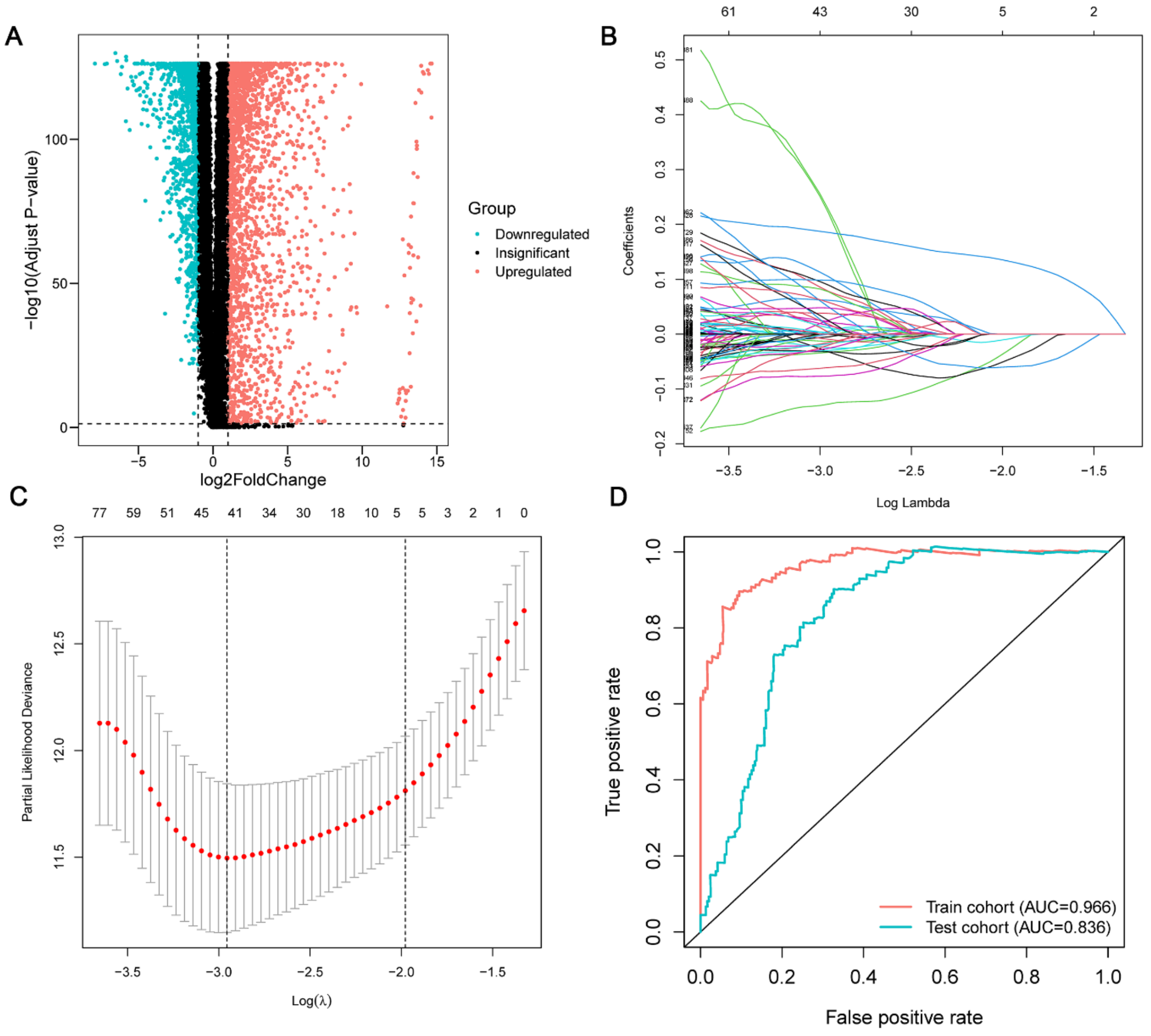

Figure S5 Comprehensive view of dysregulated pathways between MPS-I and MPS-II NBL

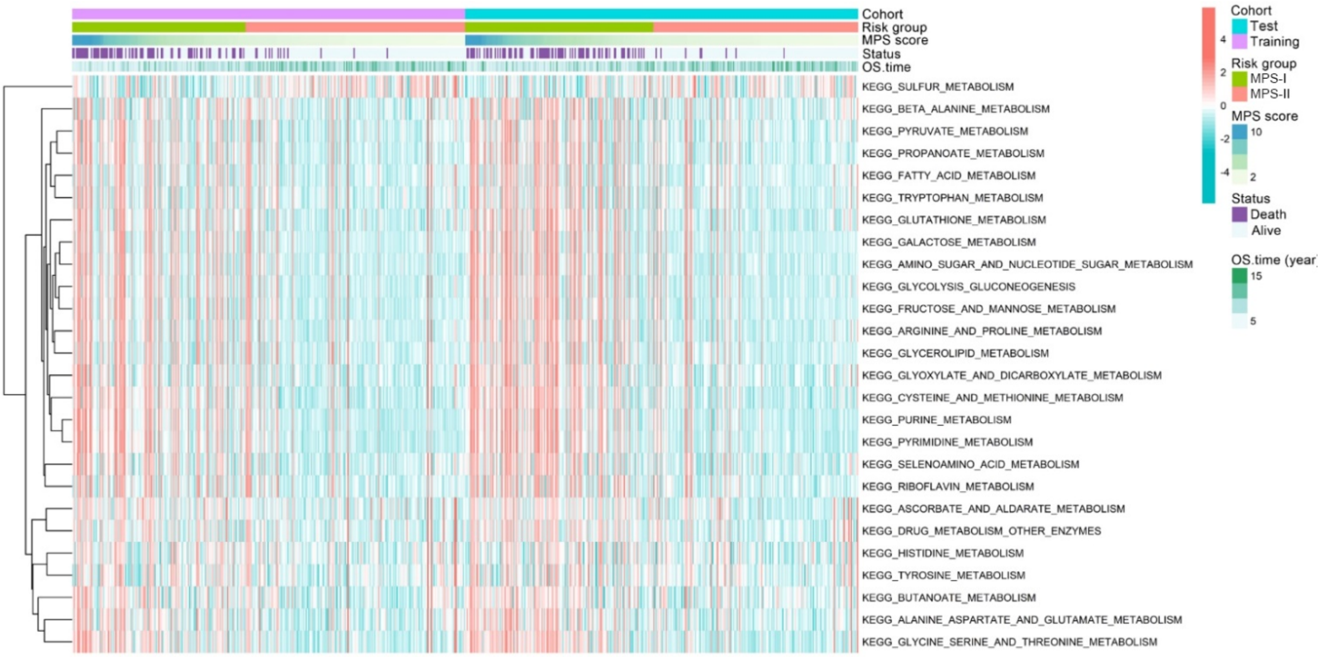

Figure S6 In the test cohort, different expressions of kPMGs in MPS-I and MPS-II NBL patients.

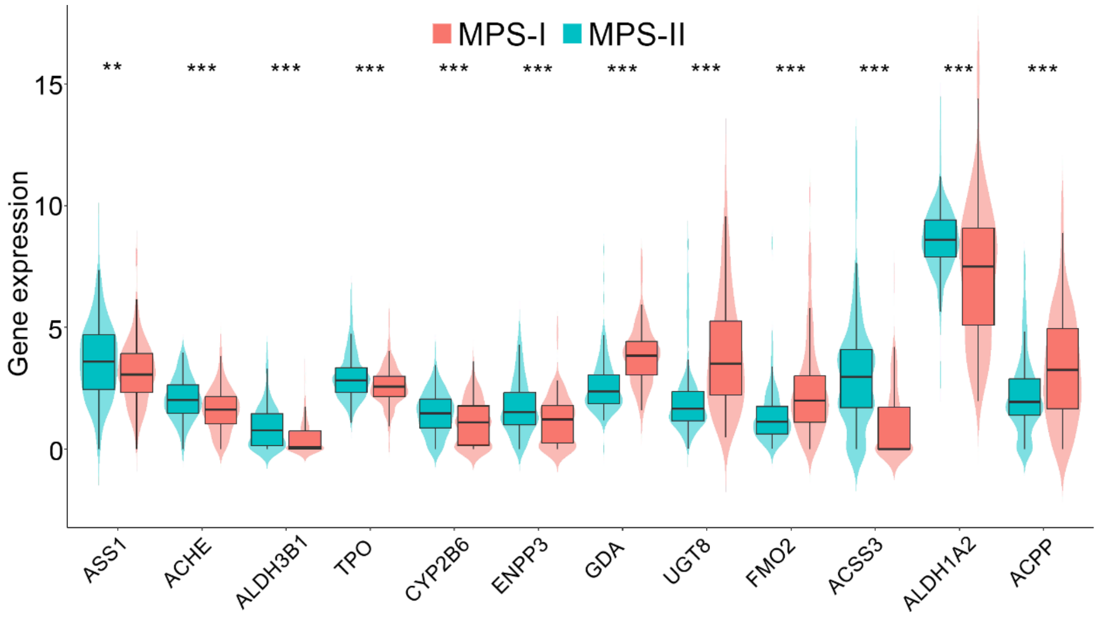

Figure S7 The infiltration atlas of immune cells upon NBL samples in the training and test cohort.

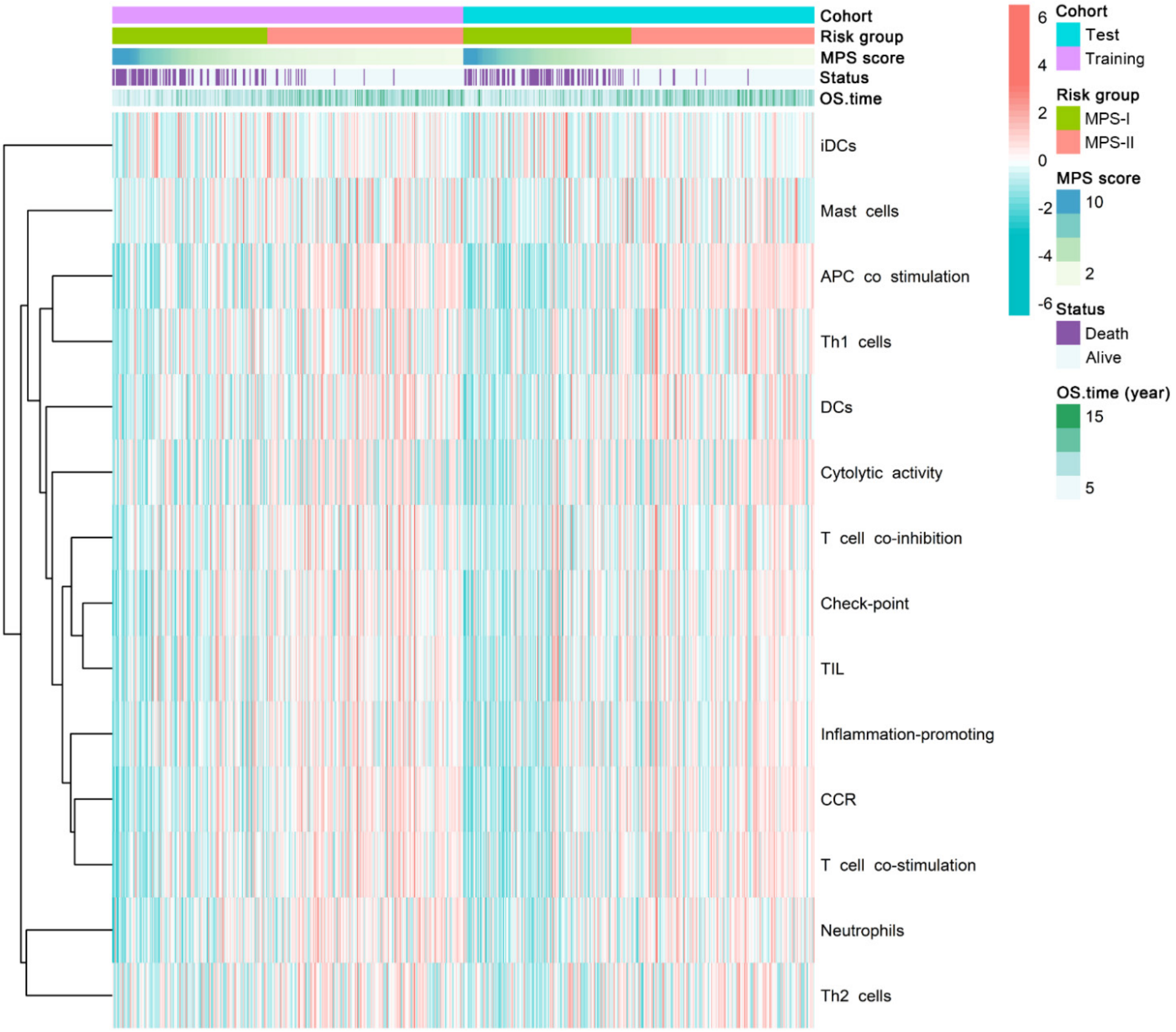

Figure S8 Necrosis levels of cells in control and treated groups.

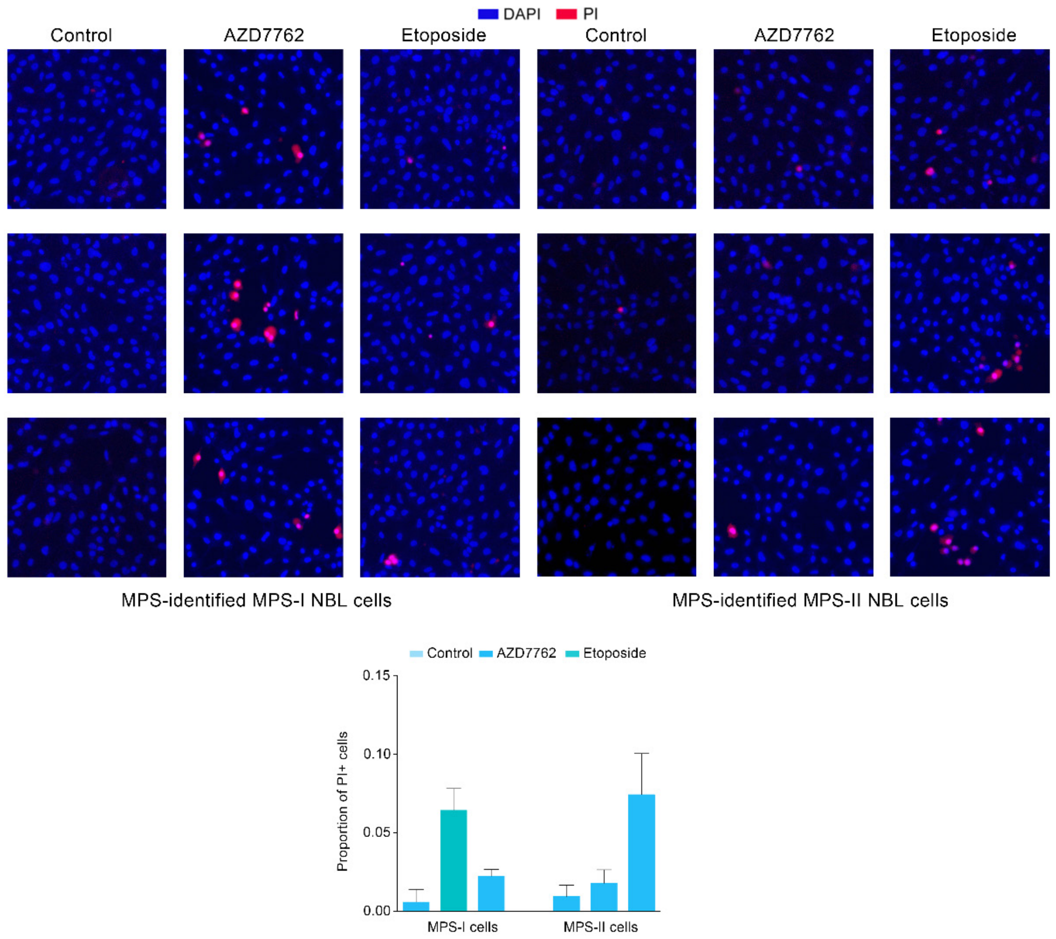

Figure S9 Nomogram of MPS.

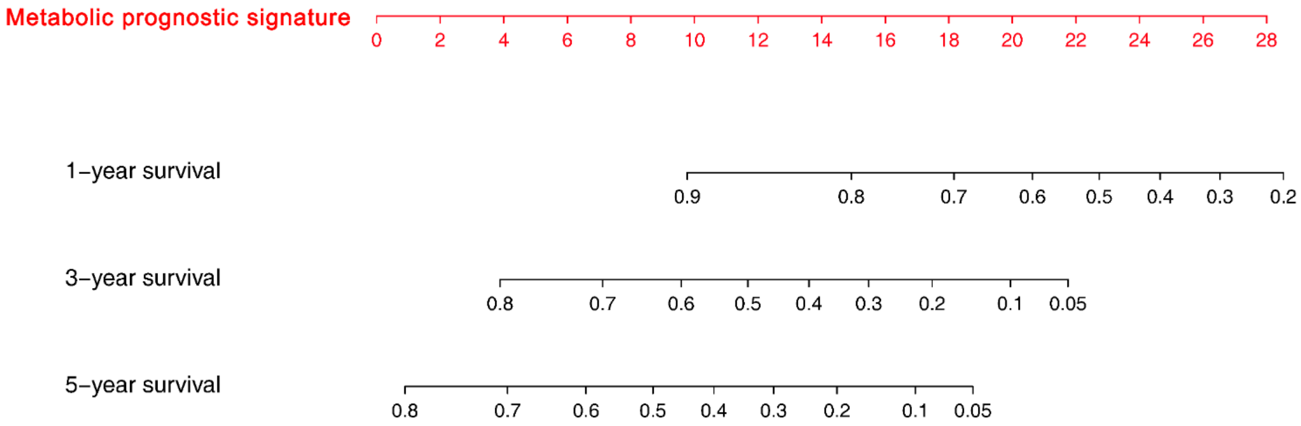

Figure S10 Quality control of scRNA-seq data

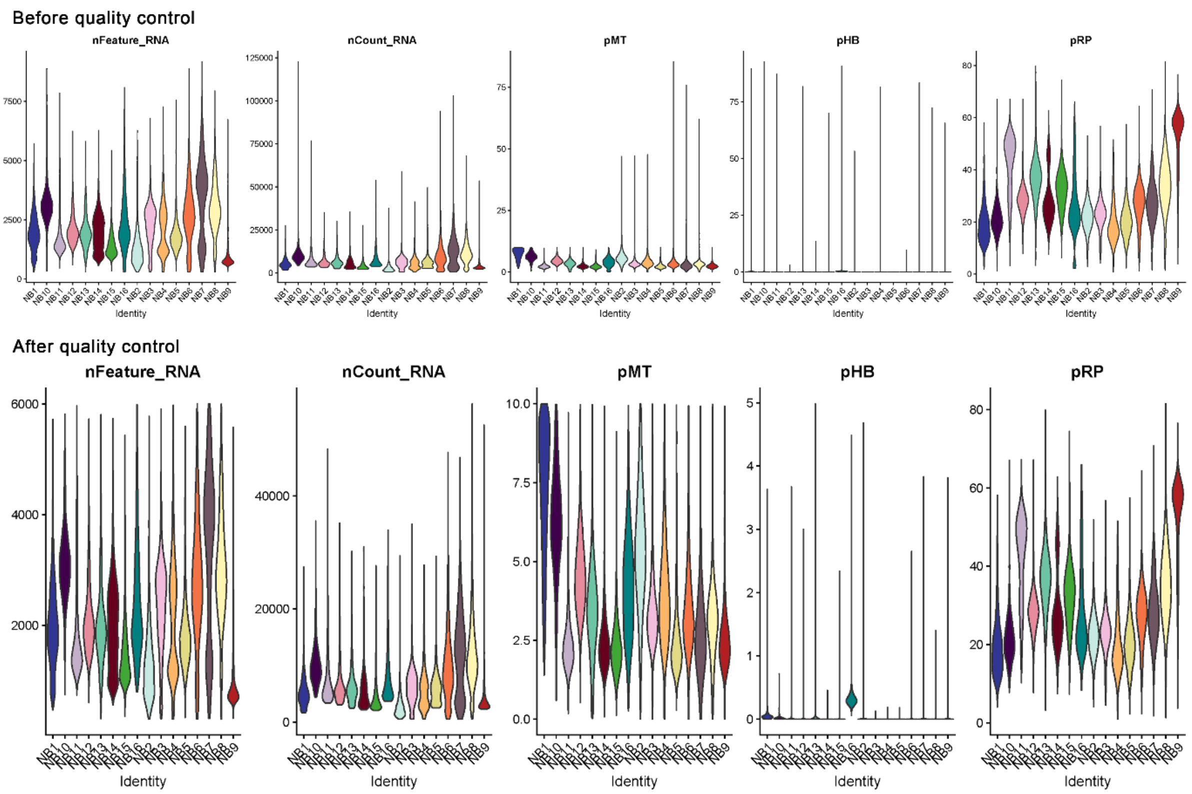

Figure S11 Clustering of scRNA-seq data

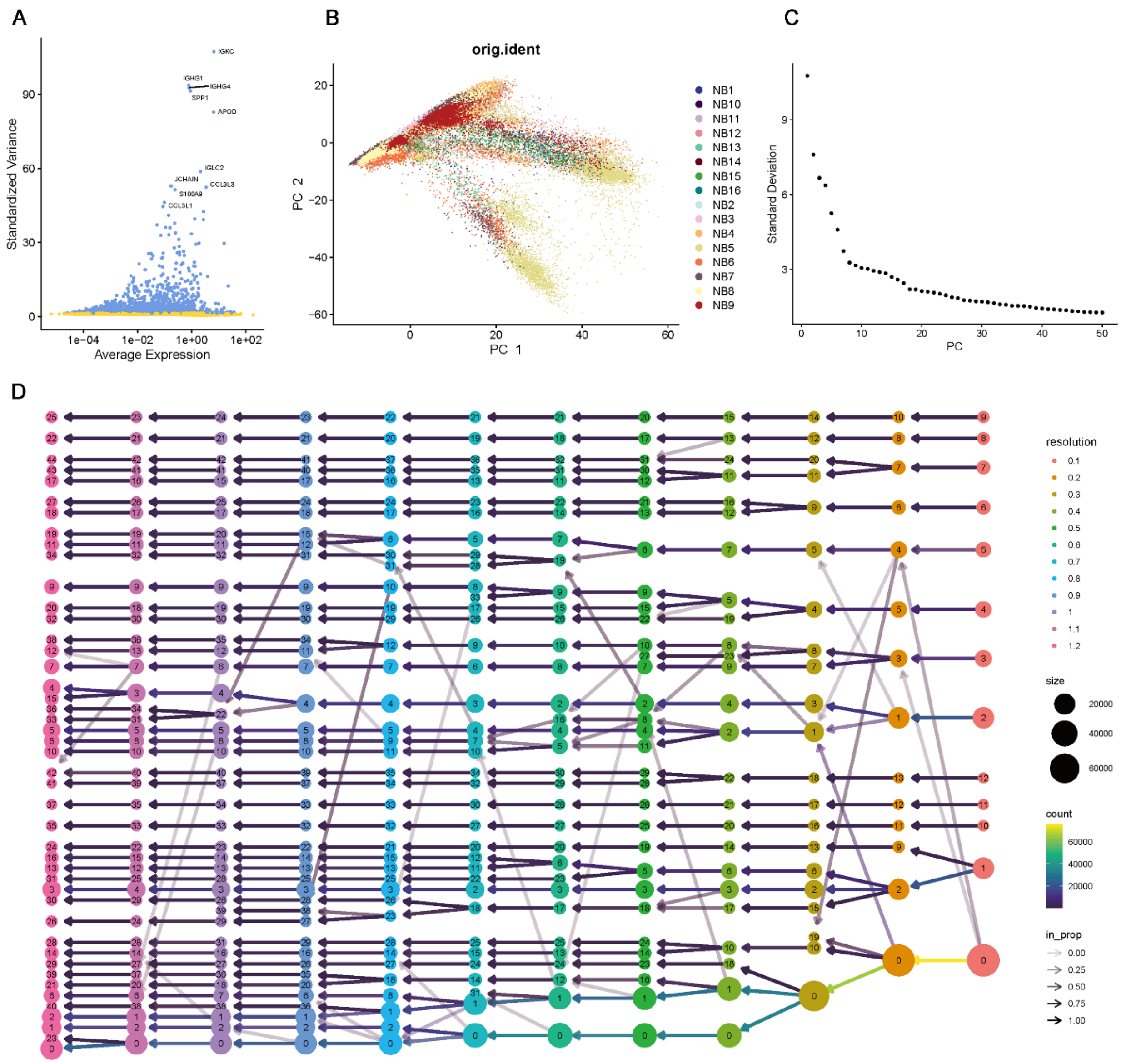

Figure S12 Annotation and proportion of cell types

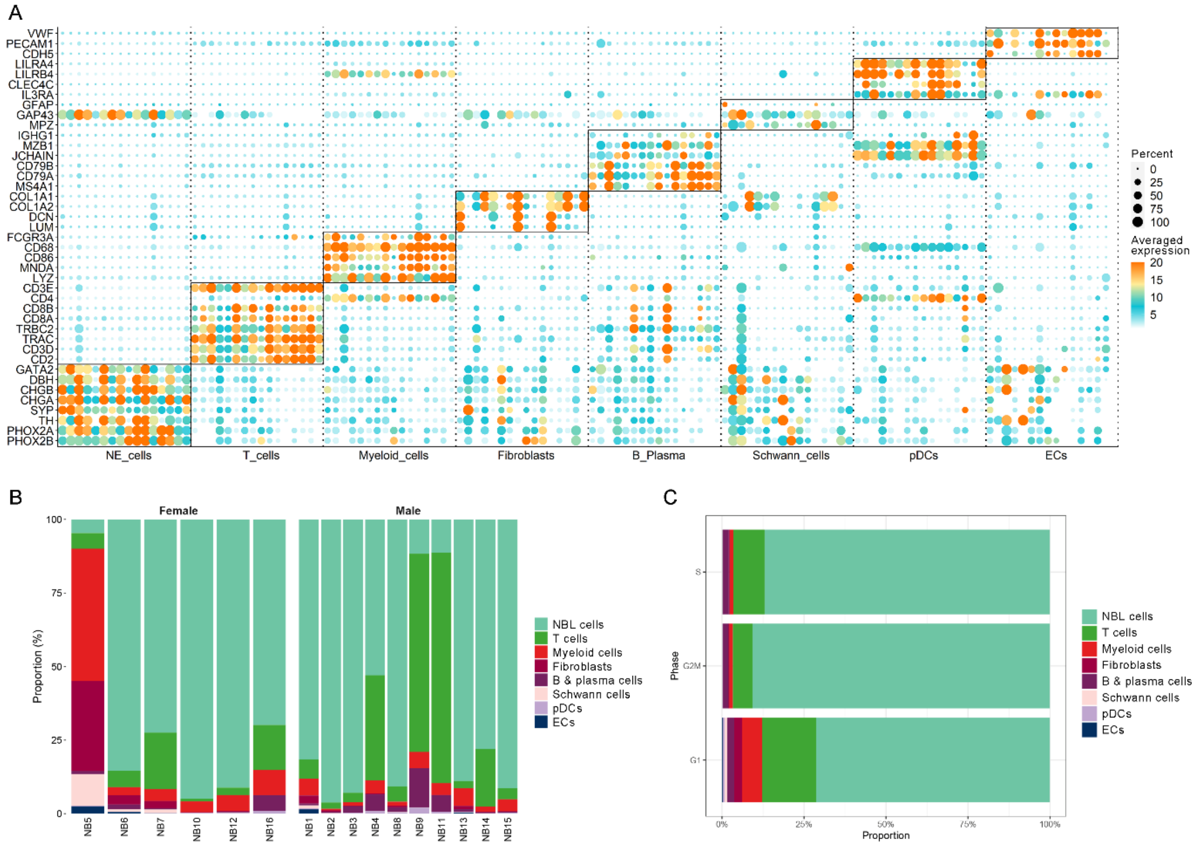

Figure S13 CNV inference

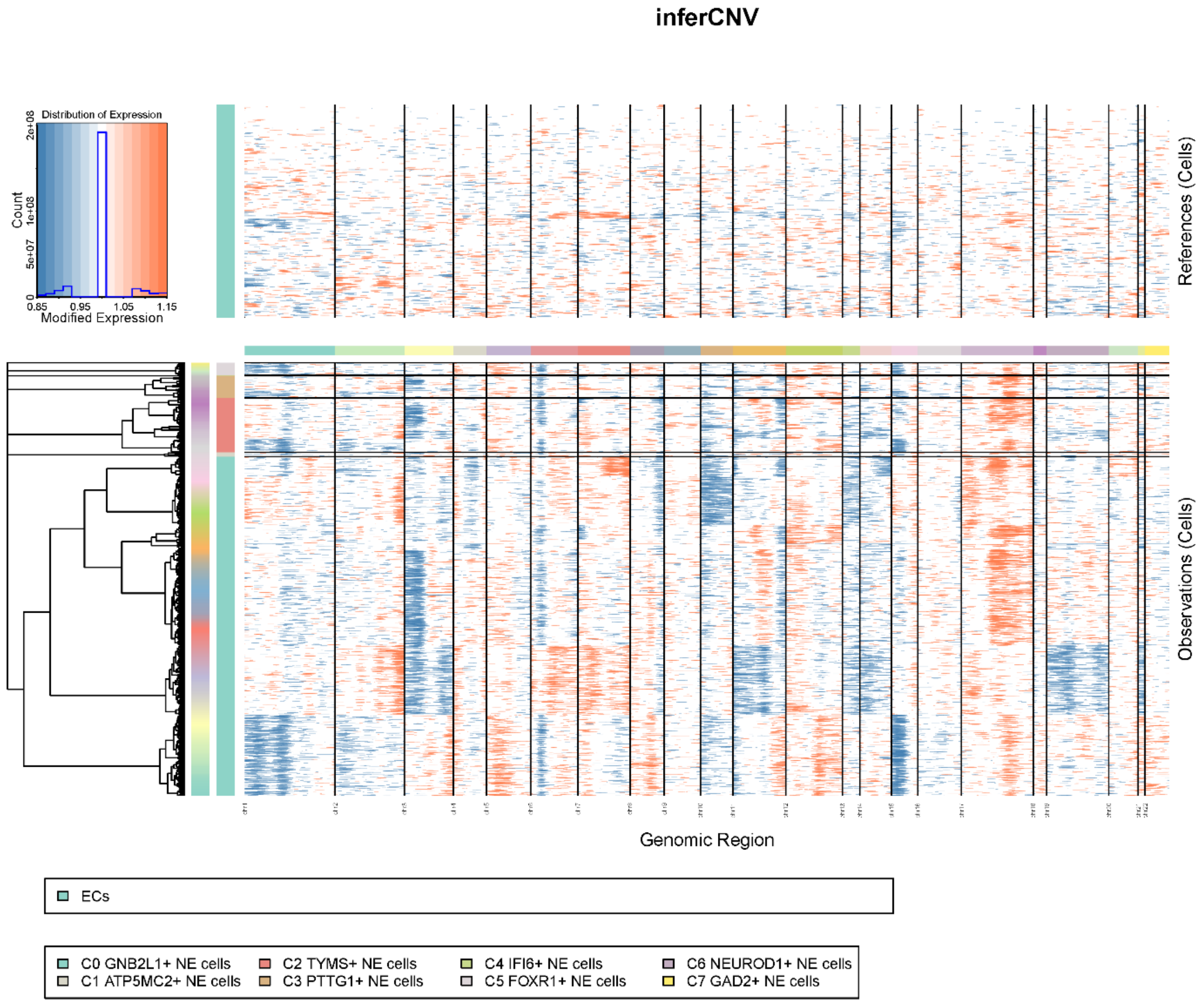

Figure S14 Major biological processes of each NBL cell type

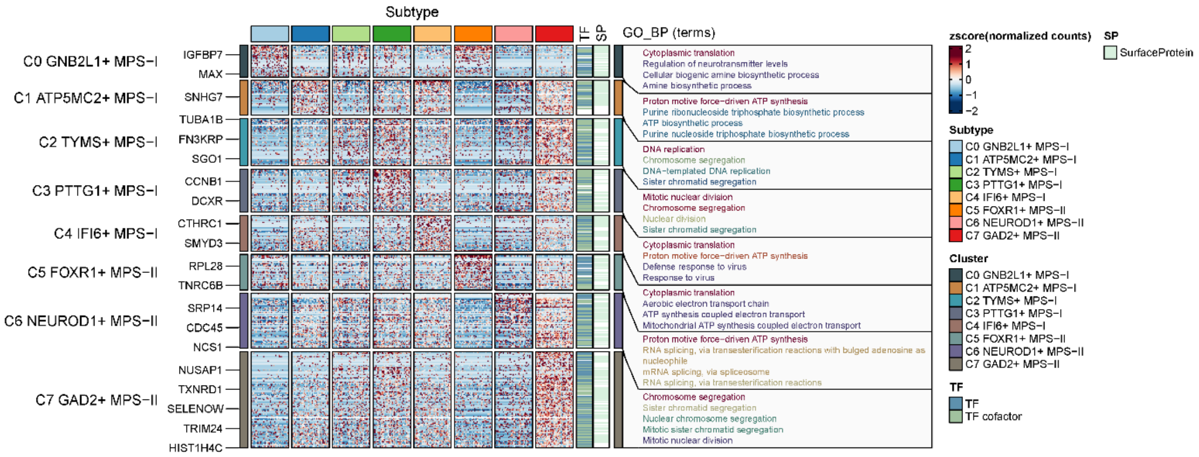

Figure S15 Differential metabolites between MPS subtyped cells

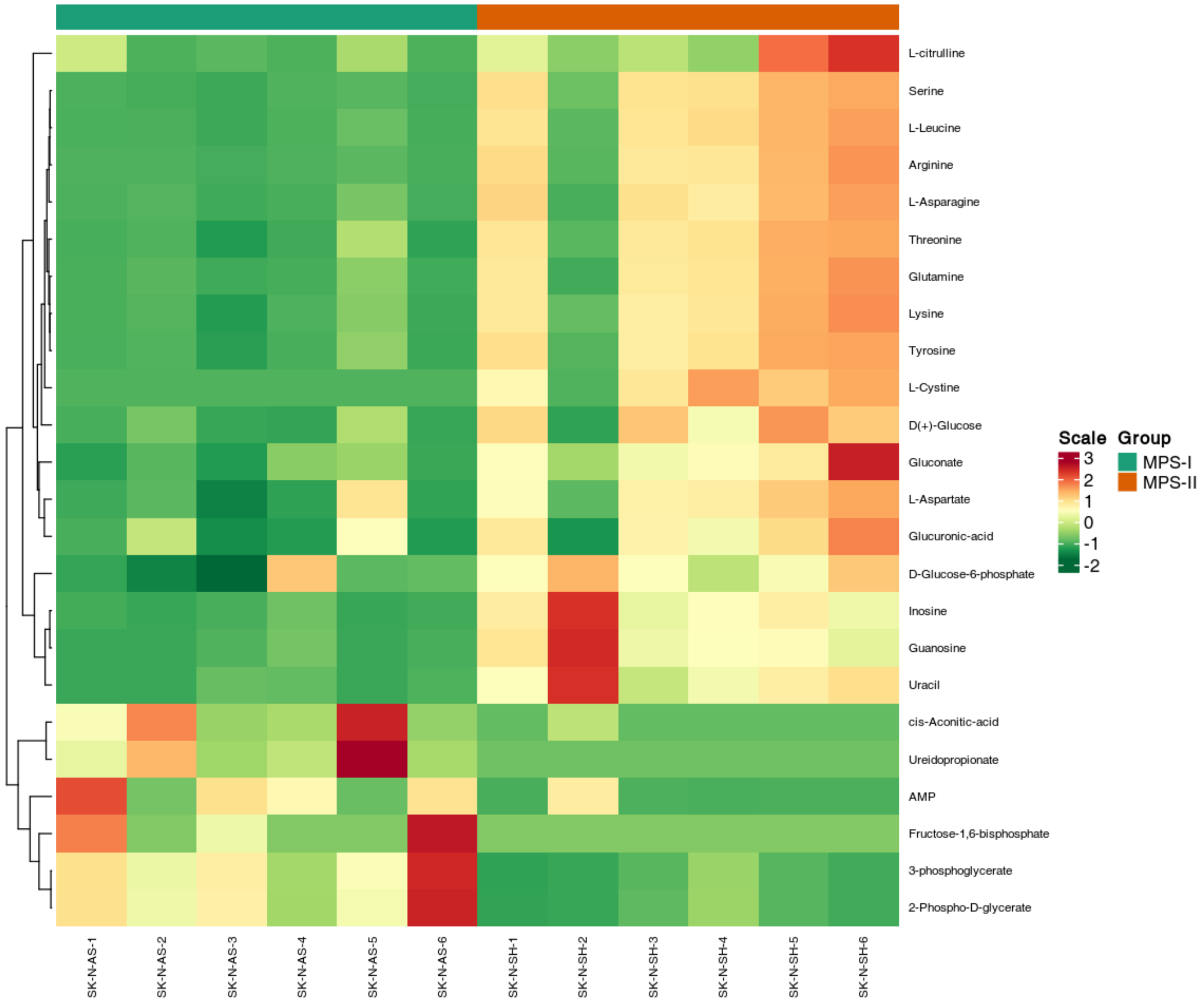

Table S1 Clinical parameters and sources for samples.

Clinical parameters		TARGET	GSE49711	GTEx	GSE85047
**Age (Month)**	≥18	117	192	NA	1
	<18	33	306	NA	275
**Gender**	Female	62	211	3679	NA
	Male	88	287	6104	NA
**MYCN**	Not Amplified	119	401	NA	218
	Amplified	30	92	NA	58
**INSS stage**	1	NA	121	NA	46
	2	1	78	NA	36
	3	9	63	NA	43
	4	120	183	NA	124
	4S	20	53	NA	27
**Survival status**	Alive	75	393	NA	201
	Dead	75	105	NA	75
**Sample size**		150	498	9783	276
**Source**		TARGET datasets collated by UCSC Xena	GEO	GTEx datasets collated by UCSC Xena	GEO
**Databases website**		xenabrowser.net	www.ncbi.nlm.nih.gov	xenabrowser.net	www.ncbi.nlm.nih.gov

Table S2 Resultant data of iterative univariable cox regression for metabolic genes.

Metabolic genes	Hazard Ratio (95% CI)	p-value	Metabolic genes	Hazard Ratio (95% CI)	p-value
FMO2	0.741(0.637-0.863)	1.11E-04	PLA2G2A	1.119(1.042-1.203)	2.05E-03
LDHA	1.124(1.068-1.184)	7.62E-06	CKMT2	1.377(1.141-1.662)	8.47E-04
OGDHL	1.282(1.121-1.466)	2.77E-04	CYP3A43	1.173(0.922-1.493)	1.93E-01
GALC	1.194(1.055-1.352)	5.02E-03	CA5A	1.015(0.906-1.138)	7.95E-01
AKR1C2	0.67(0.55-0.815)	6.35E-05	CPS1	1.079(0.93-1.251)	3.16E-01
TPO	0.636(0.499-0.811)	2.66E-04	ENPP6	1.176(1.056-1.31)	3.20E-03
PDE1C	0.805(0.642-1.008)	5.85E-02	PFKFB2	1.271(1.074-1.504)	5.36E-03
MBOAT1	1.165(0.972-1.397)	9.89E-02	CDA	1.232(1.034-1.467)	1.95E-02
XDH	1.278(1.177-1.388)	5.37E-09	DHRS3	0.994(0.877-1.126)	9.23E-01
AOC2	0.897(0.822-0.979)	1.45E-02	ADH6	1.02(0.878-1.183)	7.99E-01
ALDH3A1	1.828(1.419-2.356)	3.12E-06	BHMT	1.131(1.014-1.263)	2.72E-02
CA4	1.094(0.942-1.271)	2.40E-01	ENTPD2	0.943(0.854-1.041)	2.42E-01
HMGCS2	0.922(0.768-1.106)	3.81E-01	AOX1	1.144(0.958-1.366)	1.37E-01
G6PC2	1.017(0.686-1.508)	9.32E-01	ACPP	0.705(0.547-0.908)	6.88E-03
GUCY2F	1.002(0.697-1.439)	9.93E-01	GLDC	1.287(1.177-1.407)	3.12E-08
PCK1	0.96(0.85-1.084)	5.07E-01	ENPP3	0.624(0.486-0.8)	2.02E-04
ADH4	1.025(0.884-1.188)	7.43E-01	PDE4D	1.376(1.171-1.617)	1.04E-04
IPMK	0.894(0.825-0.97)	6.75E-03	PDE9A	1.137(0.997-1.298)	5.56E-02
ACSM1	0.835(0.644-1.081)	1.70E-01	AGMAT	0.953(0.868-1.046)	3.12E-01
AKR1B10	1.11(0.88-1.401)	3.79E-01	PNLIPRP1	2.023(1.362-3.006)	4.85E-04
BLVRB	1.076(0.985-1.176)	1.05E-01	UGDH	1.233(1.135-1.34)	7.94E-07
DGKI	0.945(0.767-1.164)	5.94E-01	CYP1B1	1.13(1.024-1.248)	1.53E-02
TH	0.977(0.898-1.064)	5.97E-01	GPD1	0.937(0.79-1.111)	4.53E-01
LCT	1.154(1.033-1.289)	1.13E-02	MAOB	1.111(1.019-1.21)	1.67E-02
PNLIP	1.146(0.589-2.231)	6.88E-01	NT5C2	1.139(1.036-1.252)	6.94E-03
GFPT2	1.142(1.005-1.298)	4.19E-02	ITPK1	1.177(1.073-1.292)	5.96E-04
ACSM2A	0.949(0.774-1.164)	6.17E-01	HK1	1.091(1.034-1.15)	1.47E-03
UPP2	0.687(0.601-0.785)	4.04E-08	DHRS4L2	1.308(1.159-1.475)	1.32E-05
GDA	0.57(0.399-0.815)	2.06E-03	INPP4B	1.15(0.953-1.388)	1.45E-01
PLA2G10	0.985(0.905-1.073)	7.35E-01	CP	1.113(1.001-1.237)	4.78E-02
CKM	1.12(0.961-1.304)	1.46E-01	LIPC	0.768(0.611-0.965)	2.33E-02
ADCY8	1.054(0.96-1.157)	2.73E-01	DDC	1.022(0.948-1.101)	5.71E-01
ALDH1A1	1.085(1.022-1.153)	7.65E-03	B4GALT1	1.142(1.053-1.239)	1.34E-03
SULT2B1	1.256(0.994-1.588)	5.64E-02	GAL3ST1	1.285(1.146-1.441)	1.73E-05
CKMT1A	1.226(1.017-1.477)	3.29E-02	LDHAL6A	1.002(0.928-1.082)	9.68E-01
AMY1C	0.727(0.618-0.856)	1.25E-04	ACSM4	0.869(0.791-0.955)	3.54E-03
GATM	0.987(0.827-1.179)	8.87E-01	ALDH3B1	1.658(1.414-1.943)	4.59E-10
NAT1	1.574(1.277-1.939)	2.05E-05	PRODH	1.033(0.824-1.296)	7.78E-01
TDO2	0.923(0.756-1.127)	4.32E-01	AKR1C4	0.887(0.821-0.958)	2.16E-03

RDH8	1.26(0.978-1.622)	7.39E-02	GLUD2	0.93(0.893-0.969)	5.37E-04
SGPP2	0.825(0.699-0.973)	2.24E-02	ASS1	1.208(1.125-1.297)	1.78E-07
NT5C1A	0.87(0.825-0.916)	1.87E-07	PLA2G4E	1.68(1.091-2.586)	1.86E-02
RPE65	0.772(0.656-0.908)	1.83E-03	DHRS9	0.877(0.677-1.136)	3.20E-01
MGST1	1.03(0.9-1.179)	6.67E-01	WARS	1.149(1.078-1.225)	2.11E-05
ADH1B	0.978(0.878-1.09)	6.88E-01	ALDH1A2	1.264(1.153-1.385)	5.07E-07
LPL	1.119(1.02-1.228)	1.69E-02	CHST13	0.879(0.816-0.947)	6.49E-04
PCCB	1.418(1.228-1.638)	2.08E-06	DGAT2	1.434(1.212-1.697)	2.77E-05
PRPS1L1	0.923(0.874-0.975)	4.26E-03	GSTA1	0.875(0.75-1.022)	9.14E-02
CYP4F3	0.934(0.762-1.146)	5.14E-01	SPHK1	1.151(1.034-1.28)	9.76E-03
P4HA2	1(0.83-1.203)	9.97E-01	ACSS1	0.927(0.753-1.141)	4.76E-01
LRAT	1.144(0.993-1.318)	6.28E-02	PIP5K1B	0.894(0.655-1.221)	4.81E-01
CKMT1B	1.134(0.998-1.287)	5.28E-02	ACADL	0.9(0.777-1.044)	1.64E-01
MGLL	1.129(0.973-1.31)	1.09E-01	ASPA	0.998(0.839-1.187)	9.83E-01
POLR1A	1.17(1.075-1.274)	3.02E-04	PLCD3	1.211(0.986-1.487)	6.84E-02
ACSS3	0.607(0.47-0.785)	1.39E-04	GGT6	1.111(0.891-1.386)	3.49E-01
GK2	1.027(0.83-1.27)	8.09E-01	ADCY1	0.741(0.654-0.84)	2.76E-06
PDE1A	1.31(1.092-1.571)	3.64E-03	UROC1	0.934(0.797-1.093)	3.94E-01
UGT2B7	0.856(0.611-1.199)	3.66E-01	CYP2J2	1.136(0.969-1.332)	1.16E-01
ACP6	1.73(1.433-2.089)	1.17E-08	CYP2B6	0.682(0.589-0.79)	3.24E-07
GUCY2D	1.05(0.97-1.136)	2.24E-01	ADH7	2.119(0.676-6.647)	1.98E-01
CYP1A1	1.527(1.289-1.81)	1.02E-06	PLA2G5	1.105(0.916-1.333)	2.98E-01
AMY1B	0.703(0.577-0.856)	4.67E-04	PIK3C2G	0.927(0.638-1.347)	6.90E-01
DBH	1.041(0.976-1.11)	2.21E-01	PNLIPRP2	1.551(1.16-2.073)	3.08E-03
CYP2A13	0.892(0.834-0.953)	6.92E-04	ALOX12	0.804(0.722-0.895)	7.22E-05
KYNU	1.254(0.984-1.598)	6.75E-02	CYP4A11	0.995(0.855-1.157)	9.44E-01
ALDH3B2	1.262(0.942-1.689)	1.18E-01	GSTT2	0.894(0.829-0.963)	3.33E-03
ACSL1	1.123(1.037-1.217)	4.49E-03	AMY1A	0.665(0.542-0.815)	8.76E-05
ACACB	0.875(0.687-1.114)	2.79E-01	SGMS2	1.174(0.976-1.412)	8.94E-02
IDO2	0.791(0.676-0.924)	3.23E-03	ACHE	0.762(0.689-0.842)	1.03E-07
HPD	1.02(0.905-1.15)	7.45E-01	GPX3	1.114(0.982-1.264)	9.46E-02
CA1	1.209(1.055-1.386)	6.34E-03	DHDH	0.902(0.836-0.974)	8.20E-03
PLA2G2D	0.889(0.837-0.944)	1.30E-04	MIOX	1.304(1.127-1.51)	3.61E-04
CES1	1.013(0.884-1.162)	8.51E-01	PLCG2	1.168(0.984-1.387)	7.64E-02
AK5	1.032(0.891-1.194)	6.77E-01	CYP4A22	0.949(0.824-1.092)	4.65E-01
MARS2	0.918(0.853-0.987)	2.08E-02	PLA2G6	1.038(0.871-1.236)	6.80E-01
PLA2G2A	1.119(1.042-1.203)	2.05E-03	ACY3	1.032(0.883-1.207)	6.94E-01
CKMT2	1.377(1.141-1.662)	8.47E-04	UGT8	1.269(1.15-1.4)	2.00E-06

Table S3 Coefficients of genetic prognostic signature based on all genes.

Genes	Coefficients	Hazard ration (95%CI)	*p* value
RNF112	-0.35301	0.703 (0.587-0.841)	0.000125
CHRNA5	0.260173	1.297 (1.062-1.585)	0.010836
PTMAP1	-0.11417	0.892 (0.831-0.958)	0.001596
SKA1	0.123819	1.132 (0.981-1.305)	0.089003
OMP	-0.13861	0.871 (0.807-0.940)	0.000373
ZYG11A	0.120345	1.128 (1.010-1.260)	0.032928
ALX3	0.085981	1.090 (1.003-1.184)	0.041121
GP2	1.314878	3.724 (1.618-8.571)	0.001988
KRT78	1.280171	3.597 (1.354-9.560)	0.01026
EDN2	-0.36395	0.695 (0.493-0.979)	0.037599
TRBVB	0.181696	1.199 (1.001-1.436)	0.04847
TERT	0.270956	1.311 (1.181-1.455)	3.49E-07
SHROOM3	0.284615	1.329 (1.064-1.661)	0.012263
FOLR1	0.246598	1.280 (1.073-1.526)	0.00608
ULBP2	-0.14238	0.867 (0.749-1.004)	0.056971
CCKBR	0.291523	1.338 (1.120-1.599)	0.001339
GPR31	-0.08326	0.920 (0.851-0.995)	0.037634
RAC3	0.38123	1.464 (1.153-1.858)	0.001728
HIST1H2BM	0.073953	1.077 (0.992-1.169)	0.076397
FBXO43	0.356414	1.428 (1.179-1.730)	0.000272
ECEL1	-0.20408	0.815 (0.736-0.903)	9.58E-05
FA2H	0.323697	1.382 (1.026-1.861)	0.033061
PLA2G16	0.356639	1.429 (1.162-1.756)	0.000699
OR13C2	-0.08398	0.919 (0.843-1.003)	0.057964
ARSF	-0.19853	0.820 (0.689-0.975)	0.024772

Table S4 Dysregulated pathways between MPS-I and MPS-II NBL

Pathways	Training cohort		Test cohort	
	log_2FC	*p*-value	log_2FC	*p*-value
KEGG_GLYCOLYSIS_GLUCONEOGENESIS	0.157953	6.06E-08	0.211855	6.88E-10
KEGG_FRUCTOSE_AND_MANNOSE_METABOLISM	0.210444	6.20E-07	0.279816	4.14E-10
KEGG_GALACTOSE_METABOLISM	0.246784	0.000211	0.353769	3.19E-07
KEGG_ASCORBATE_AND_ALDARATE_METABOLISM	0.143018	1.19E-05	0.183875	1.32E-07
KEGG_FATTY_ACID_METABOLISM	0.069631	0.039824	0.108408	0.015817
KEGG_PURINE_METABOLISM	0.131546	6.61E-16	0.151351	4.86E-16
KEGG_PYRIMIDINE_METABOLISM	0.144599	3.67E-17	0.164042	7.88E-18
KEGG_ALANINE_ASPARTATE_AND_GLUTAMATE_METABOLISM	0.07133	3.49E-05	0.106139	8.00E-09
KEGG_GLYCINE_SERINE_AND_THREONINE_METABOLISM	0.182945	2.81E-11	0.24382	1.50E-16
KEGG_CYSTEINE_AND_METHIONINE_METABOLISM	0.157645	2.51E-15	0.184298	3.26E-16
KEGG_ARGININE_AND_PROLINE_METABOLISM	0.109048	4.37E-06	0.16039	5.55E-10
KEGG_HISTIDINE_METABOLISM	0.032918	0.009636	0.044871	0.008952
KEGG_TYROSINE_METABOLISM	0.033974	0.021163	0.038263	0.041415
KEGG_TRYPTOPHAN_METABOLISM	0.067595	0.019518	0.110578	9.07E-05
KEGG_BETA_ALANINE_METABOLISM	0.042425	0.028213	0.067803	0.000915
KEGG_SELENOAMINO_ACID_METABOLISM	0.088657	2.27E-06	0.111369	2.12E-09
KEGG_GLUTATHIONE_METABOLISM	0.115313	1.45E-07	0.16032	1.60E-11
KEGG_AMINO_SUGAR_AND_NUCLEOTIDE_SUGAR_METABOLISM	0.173223	1.09E-05	0.258307	4.70E-11
KEGG_GLYCEROLIPID_METABOLISM	0.107567	0.030849	0.165982	4.60E-05
KEGG_PYRUVATE_METABOLISM	0.060979	0.000278	0.084408	2.24E-05
KEGG_GLYOXYLATE_AND_DICARBOXYLATE_METABOLISM	0.113132	3.43E-06	0.146818	3.00E-09
KEGG_PROPANOATE_METABOLISM	0.117283	2.53E-07	0.150817	2.94E-07
KEGG_BUTANOATE_METABOLISM	0.049278	8.27E-06	0.047147	1.81E-05
KEGG_RIBOFLAVIN_METABOLISM	0.146445	0.002252	0.201661	2.56E-05
KEGG_SULFUR_METABOLISM	-0.08488	5.74E-06	-0.05354	0.001129
KEGG_DRUG_METABOLISM_OTHER_ENZYMES	0.05867	0.0054	0.118372	6.96E-06

Table S5 Biological processes, cell components and molecular functions overrepresented by aberrantly expressed MGs.

Ontology	Description	p-value	FDR	Count
BP	Organic hydroxy compound metabolic process	1.70E-36	3.03E-33	47
	Alcohol metabolic process	1.28E-29	1.14E-26	36
	Small molecule catabolic process	4.27E-19	1.52E-16	29
	Ammonium ion metabolic process	3.30E-20	1.47E-17	23
	Primary alcohol metabolic process	1.02E-24	6.07E-22	20
	Alpha-amino acid metabolic process	3.55E-16	7.04E-14	20
	Isoprenoid metabolic process	2.04E-16	4.55E-14	17
	Retinoid metabolic process	3.93E-17	1.17E-14	16
	Diterpenoid metabolic process	1.15E-16	2.92E-14	16
	Terpenoid metabolic process	5.01E-16	8.93E-14	16
CC	Organelle outer membrane	2.31E-05	2.29E-03	9
	Outer membrane	2.50E-05	2.29E-03	9
	Mitochondrial matrix	6.35E-05	3.87E-03	13
	Mitochondrial outer membrane	4.04E-04	1.85E-02	7
MF	Cofactor binding	1.52E-19	4.08E-17	31
	Oxidoreductase activity, acting on CH-OH group of donors	4.26E-16	5.71E-14	17
	Coenzyme binding	1.23E-10	3.65E-09	17
	Oxidoreductase activity, acting on the CH-OH group of donors, NAD or NADP as acceptor	1.56E-15	1.40E-13	16
	Carboxylic ester hydrolase activity	8.71E-15	5.84E-13	16
	Iron ion binding	2.49E-12	9.52E-11	15
	Monooxygenase activity	5.94E-13	3.19E-11	13
	Oxidoreductase activity, acting on the aldehyde or oxo group of donors	1.10E-12	4.91E-11	10
	Aldo-keto reductase (NADP) activity	1.35E-11	4.54E-10	8
	Oxidoreductase activity, acting on the aldehyde or oxo group of donors, NAD or NADP as acceptor	4.62E-10	1.24E-08	8

FDR represents a p-value corrected by FDR method, only top 10 GO terms in each aspect were shown.

Abbreviations: biological processes (BP); cell components (CC); molecular functions (MF);

Table S6 Pathways overrepresented by aberrantly expressed MGs.

Description	p-value	FDR	Count
Retinol metabolism	1.07E-16	1.02E-14	18
Chemical carcinogenesis	8.66E-14	5.51E-12	17
Metabolism of xenobiotics by cytochrome P450	3.75E-13	1.79E-11	16
Purine metabolism	1.79E-09	4.27E-08	16
Tyrosine metabolism	2.13E-17	4.07E-15	15
Drug metabolism - cytochrome P450	2.50E-12	9.55E-11	15
Arachidonic acid metabolism	8.80E-11	2.80E-09	13
Glycolysis / Gluconeogenesis	2.43E-10	6.64E-09	13
Glycerolipid metabolism	1.19E-08	2.27E-07	11
Phenylalanine metabolism	1.11E-08	2.27E-07	7

FDR represents a p-value corrected by FDR method, only top 10 pathways were shown.

Table S7 Biological processes and molecular functions overrepresented by kPMGs.

	Description	FDR
Biological Process	drug metabolic process	4.62E-09
	small molecule metabolic process	2.38E-05
	drug catabolic process	1.20E-04
	small molecule biosynthetic process	2.30E-03
	midgut development	4.00E-03
	cellular metabolic process	7.40E-03
	purine nucleobase metabolic process	8.00E-03
	cellular response to xenobiotic stimulus	8.70E-03
	organic substance metabolic process	8.70E-03
	oxidation-reduction process	9.10E-03
	cellular lipid metabolic process	9.30E-03
	organophosphate metabolic process	9.80E-03
	hormone metabolic process	9.80E-03
	cellular catabolic process	9.80E-03
	lipid biosynthetic process	1.11E-02
	biosynthetic process	1.11E-02
	nucleotide metabolic process	1.11E-02
	antibiotic catabolic process	1.11E-02
	cellular aldehyde metabolic process	1.79E-02
	organonitrogen compound metabolic process	1.79E-02
	primary alcohol metabolic process	1.93E-02
	sphingolipid biosynthetic process	2.85E-02
	pyrimidine-containing compound metabolic process	3.27E-02
	cellular biosynthetic process	3.62E-02
	organic acid metabolic process	3.84E-02
	xenobiotic metabolic process	3.84E-02
	nucleoside phosphate catabolic process	3.84E-02
	organic substance biosynthetic process	3.84E-02
	organic hydroxy compound metabolic process	3.84E-02
	vitamin metabolic process	4.05E-02
	liver development	4.34E-02
	response to estradiol	4.34E-02
	cellular response to chemical stimulus	4.34E-02
	purine-containing compound metabolic process	4.34E-02
	granulocyte activation	4.42E-02
	myeloid cell activation involved in immune response	4.51E-02
	sphingolipid metabolic process	4.51E-02
	fatty acid derivative metabolic process	4.63E-02
	protein tetramerization	4.90E-02

Molecular Function	catalytic activity	3.51E-05
	3-chloroallyl aldehyde dehydrogenase activity	4.20E-04
	aldehyde dehydrogenase (NAD) activity	1.70E-03
	oxidoreductase activity	1.70E-03
	monooxygenase activity	2.44E-02
	oxidoreductase activity, acting on paired donors, with incorporation or reduction of molecular oxygen	4.04E-02
	ligase activity	4.04E-02
	heme binding	4.04E-02
	cofactor binding	4.04E-02

Table S8 KEGG pathways analyses for kPMGs.

Description	FDR
Metabolic pathways	9.44E-08
Drug metabolism - cytochrome P450	1.30E-04
Tyrosine metabolism	2.20E-03
Retinol metabolism	4.80E-03
Metabolism of xenobiotics by cytochrome P450	4.80E-03
Purine metabolism	2.31E-02

Table S9 Univariable Cox regression for ALDH1A2.

Cohort	Hazard ratio (95% CI)	p-value	Factor
**Train**	1.26 (1.15-1.38)	5.07E-07	Hazardous factor
**Test**	1.25(1.14-1.36)	9.50E-07	Hazardous factor

Abbreviation: confidence interval (CI)

Table S10 Regulation network of transcription factors and kPMGs.

TFs	kPMGs	Correlation coefficient	p-value	Regulation
AFF4	ALDH3B1	0.453367	1.26E-17	positive
AFF4	ASS1	0.727443	5.92E-54	positive
AFF4	ALDH1A2	0.507778	2.24E-22	positive
AFF4	CYP2B6	-0.47562	1.82E-19	negative
ARNTL	ASS1	0.604341	3.04E-33	positive
ARNTL	ALDH1A2	0.407858	2.95E-14	positive
ASCL1	ASS1	-0.49236	6.07E-21	negative
CBX2	ASS1	-0.57458	1.66E-29	negative
CBX2	ACHE	-0.43204	5.53E-16	negative
EGR2	ASS1	0.475754	1.77E-19	positive
ELF1	ALDH3B1	0.433403	4.38E-16	positive
ELF1	ASS1	0.663736	5.26E-42	positive
ELF1	ALDH1A2	0.420051	4.13E-15	positive
IRF5	ALDH3B1	0.486779	1.92E-20	positive
IRF5	ASS1	0.654389	1.70E-40	positive
IRF5	ALDH1A2	0.452395	1.50E-17	positive
KLF5	ASS1	0.401592	7.86E-14	positive
LIN9	ASS1	-0.56789	1.02E-28	negative
LIN9	ACHE	-0.42109	3.48E-15	negative
MXI1	ALDH3B1	0.435142	3.24E-16	positive
MXI1	ASS1	0.698005	4.85E-48	positive
MXI1	ALDH1A2	0.465068	1.41E-18	positive
MXI1	CYP2B6	-0.44673	4.20E-17	negative
MYBL2	ASS1	0.46906	6.55E-19	positive
MYBL2	ALDH1A2	0.463221	2.00E-18	positive
MYBL2	CYP2B6	-0.44217	9.48E-17	negative
NCAPG	FMO2	-0.43324	4.50E-16	negative
NCAPG	ACHE	-0.42182	3.08E-15	negative
NR3C1	ALDH3B1	0.410918	1.82E-14	positive
NR3C1	ASS1	0.695792	1.26E-47	positive
NR3C1	ALDH1A2	0.453272	1.28E-17	positive
NR3C1	CYP2B6	-0.41179	1.58E-14	negative
PAX5	ASS1	0.489496	1.10E-20	positive
PAX6	ALDH3B1	0.465993	1.18E-18	positive
PAX6	ASS1	0.700221	1.84E-48	positive
PAX6	ALDH1A2	0.500515	1.08E-21	positive
PAX6	CYP2B6	-0.49079	8.41E-21	negative
PPARG	ASS1	0.519274	1.71E-23	positive
RARG	ALDH3B1	0.453614	1.20E-17	positive
RARG	ASS1	0.686515	6.34E-46	positive
RARG	ALDH1A2	0.440089	1.37E-16	positive
RFX2	ALDH3B1	0.446457	4.42E-17	positive

RFX2	ASS1	0.660479	1.79E-41	positive
RFX2	ALDH1A2	0.4158	8.28E-15	positive
SMAD3	ALDH3B1	0.48881	1.27E-20	positive
SMAD3	ASS1	0.757038	9.79E-61	positive
SMAD3	ALDH1A2	0.450962	1.96E-17	positive
SOX4	ALDH3B1	-0.50865	1.85E-22	negative
SOX4	ASS1	-0.7115	1.17E-50	negative
SOX4	ALDH1A2	-0.49086	8.30E-21	negative
SOX4	CYP2B6	0.421345	3.34E-15	positive
STAT6	ASS1	0.606905	1.39E-33	positive
TCF7L1	ASS1	0.603849	3.53E-33	positive
TCF7L2	ASS1	0.478731	9.80E-20	positive
TEAD4	ALDH3B1	0.508358	1.97E-22	positive
TEAD4	ASS1	0.695834	1.24E-47	positive
WWTR1	ALDH3B1	0.417539	6.24E-15	positive
WWTR1	ASS1	0.633475	2.65E-37	positive

Table S11 Dysregulated immune cellular components between MPS-I and MPS-II NBL.

Immune cellular components	Training cohort		Test cohort	
	log_2FC	p-value	log_2FC	p-value
APC co-stimulation	-0.274	7.26E-13	-0.27478	4.59E-12
CCR	-0.12972	9.99E-13	-0.11903	3.15E-10
Check-point	-0.10625	5.57E-07	-0.08085	0.000153
Cytolytic activity	-0.17838	1.67E-05	-0.19619	6.63E-05
DCs	-0.30848	1.29E-07	-0.27287	3.19E-06
iDCs	-0.11308	0.002849	0.077968	0.039349
Inflammation-promoting	-0.151	1.95E-05	-0.13196	0.000762
Mast cells	-0.21276	0.04006	-0.19652	0.014358
Neutrophils	-0.08698	2.80E-08	-0.07581	2.84E-06
T cell co-inhibition	-0.1052	9.94E-05	-0.08029	0.001504
T cell co-stimulation	-0.21422	2.97E-09	-0.22057	9.77E-09
Th1 cells	-0.15116	7.08E-05	-0.19037	7.67E-06
Th2 cells	-0.08322	0.001607	-0.0868	0.000411
TIL	-0.12699	2.48E-06	-0.09884	0.000173

Table S12 Dysregulated inflammatory components between MPS-I and MPS-II NBL.

GO Accession	Training cohort		Test cohort	
	log_2FC	*p*-value	log_2FC	*p*-value
GO:0002349	0.150354	3.84E-06	0.1927	1.65E-10
GO:0002438	-0.12744	5.45E-09	-0.07909	0.000294
GO:0002536	0.09041	0.000115	0.138653	7E-09
GO:0002544	-0.08713	6.23E-05	-0.08006	0.000247
GO:0002673	-0.0965	6.15E-08	-0.07251	8.65E-05
GO:0002675	-0.0824	0.000212	-0.05503	0.017238
GO:0002676	-0.20808	3.38E-12	-0.20587	4.93E-11
GO:0002677	-0.19176	5.92E-06	-0.1862	1.5E-05
GO:0002861	-0.10478	1.59E-07	-0.06625	0.001252
GO:0002862	-0.16528	6.41E-13	-0.13284	2.67E-08
GO:0002865	-0.3498	1.06E-09	-0.29473	4.66E-06
GO:0044546	0.081589	0.00057	0.093742	0.000145
GO:0060266	0.093534	0.000416	0.148498	6.71E-08
GO:0106014	0.095634	0.001428	0.175741	5.71E-08
GO:0150078	-0.13232	2.06E-06	-0.0945	0.000846
GO:1900227	0.073134	0.000399	0.05206	0.016377
GO:0072557	-0.16204	9.33E-12	-0.10831	8.45E-06
GO:0097169	-0.09003	0.032236	0.09094	0.039518
